# Room temperature C(sp^2^)–H oxidative chlorination *via* photoredox catalysis†
†Electronic supplementary information (ESI) available. See DOI: 10.1039/c7sc03010j



**DOI:** 10.1039/c7sc03010j

**Published:** 2017-08-15

**Authors:** Lei Zhang, Xile Hu

**Affiliations:** a Laboratory of Inorganic Synthesis and Catalysis , Institute of Chemical Sciences and Engineering , Ecole Polytechnique Fédérale de Lausanne (EPFL) , ISCI-LSCI , BCH 3305 , 1015 Lausanne , Switzerland . Email: xile.hu@epfl.ch ; http://lsci.epfl.ch

## Abstract

Photoredox catalysis has been developed to achieve oxidative C–H chlorination of aromatic compounds using NaCl as the chlorine source and Na_2_S_2_O_8_ as the oxidant. The reactions occur at room temperature and exhibit exclusive selectivity for C(sp^2^)–H bonds over C(sp^3^)–H bonds. The method has been used for the chlorination of a diverse set of substrates, including the expedited synthesis of key intermediates to bioactive compounds and a drug.

## Abstract 2

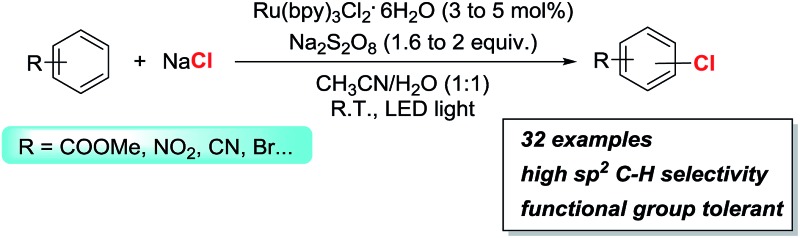
Photoredox catalysis has been developed to achieve room temperature oxidative C–H chlorination of aromatic compounds using NaCl as the chlorine source and Na_2_S_2_O_8_ as the oxidant.

## 

Chlorinated aromatic compounds exist extensively as natural products, synthetic pharmaceuticals, pesticides, and materials (Scheme 1).^1^ Moreover, they are versatile starting materials and synthetic intermediates in organic synthesis, for example, in transition-metal-catalysed cross-coupling reactions. Thus, efficient methods to synthesize chlorinated aromatic compounds are of significant interest. Direct C(sp^2^)–H electrophilic chlorination is an appealing strategy for aryl chlorination, because no directing or leaving group needs to be pre-installed on the substrates, leading to wider substrate availability, lower cost, and better step-economy. However, traditional electrophilic chlorination methods employ either toxic, hazardous, and corrosive chlorine gas, or organic electrophilic chlorination reagents, such as *N*-chlorosuccinimide (NCS) and *t*BuOCl,^2–6^ which are prepared from Cl_2_ and generate significant amounts of organic waste.

**Scheme 1:**
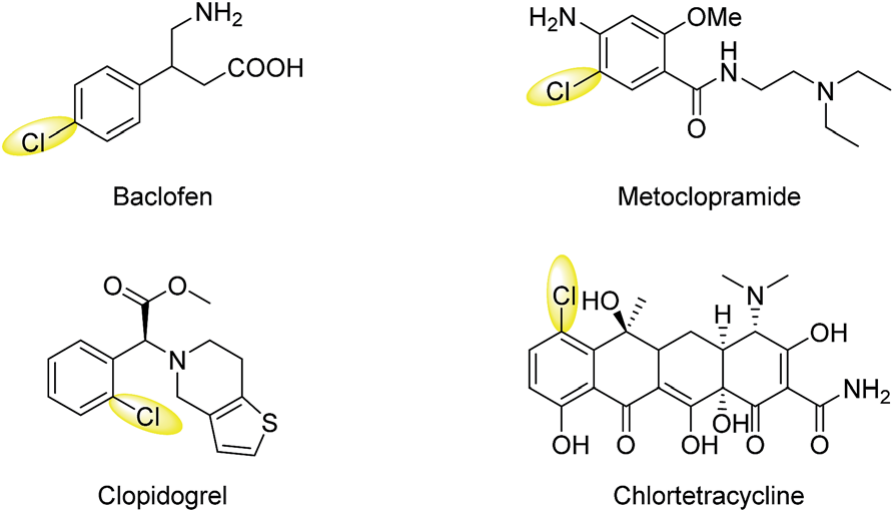
Selected examples of pharmaceuticals and natural products containing an aryl-Cl moiety.

In nature, halogenases have evolved to carry out C–H chlorination using the benign chloride anion in an oxidative manner.^7^ Inspired by nature, synthetic systems for oxidative chlorination have been developed,^8,9^ but their scope and selectivity still need to be improved. Additionally, the majority of these systems employ H_2_O_2_ as the oxidant and operate under acidic conditions,^10^ which imposes a constraint in the functional group compatibility. Konig, Wolf, and co-workers^11^ recently reported an elegant photoredox approach for oxidative chlorination using dioxygen as the oxidant. Photoexcitation of the organic dye, riboflavin tetraacetate (RFT), followed by quenching with O_2_, generated H_2_O_2_ *in situ*, which oxidized acetic acid to peracetic acid. The latter in turn oxidized Cl^–^ to OCl^–^, which was the active species for electrophilic chlorination of a number of aromatic substrates. Notwithstanding the novelty of this approach, at this early stage of its development, 6–10 equivalents of acetic acid, HCl, and *p*-methoxy benzyl alcohol (to regenerate the dye) were necessary and the scope was limited to simple arenes containing a methoxy, amino or amide group. Zhang and co-workers^12^ used potassium persulfate, a convenient inorganic oxidant for oxidative chlorination. However, a high temperature (95–100 °C) was needed and a sulphonamide group was essential to improve the efficiency through formation of a proposed N–Cl intermediate. In several cases C(sp^3^)–H chlorination was preferred over C(sp^2^)–H chlorination.

A key challenge in electrophilic C(sp^2^)–H chlorination is to achieve selectivity over competing C(sp^3^)–H chlorination. However, literature data show the opposite selectivity for substrates containing weak benzylic and α-carbonyl C(sp^3^)–H bonds. For example, acetophenone was exclusively chlorinated at the α-C(sp^3^) position under oxidative chlorination conditions using either H_2_O_2_ or O_2_ as oxidant [eqn (1)].^11^ Likewise, methyl 4-(chloromethyl)benzoate was only chlorinated at the benzylic C(sp^3^) position using K_2_S_2_O_8_ as oxidant at 100 °C [eqn (2)].^12^ To further illustrate this issue, toluene, which contains both C(sp^2^)–H bonds and benzylic C(sp^3^)–H bonds, was subjected to oxidative chlorination conditions at 100 °C using K_2_S_2_O_8_ as oxidant [eqn (3)]. The C(sp^3^)–H chlorination product, chloromethylbenzene, was obtained in 45% yield while the C(sp^2^)–H chlorination product was not formed. This undesired selectivity probably originates from the frequent formation of Cl radicals under electrophilic chlorination conditions, which react faster with weaker C(sp^3^)–H bonds than with the stronger C(sp^2^)–H bonds. For the broad applicability of oxidative C(sp^2^)–H chlorination, this selectivity problem has to be resolved. Another challenge is to develop methods that operate under mild conditions, which are essential to achieve high functional group compatibility. Here we describe a room-temperature oxidative chlorination method to selectively chlorinate aryl C–H bonds in the presence of benzylic and α-carbonyl C(sp^3^)–H bonds. Key to achieving this selectivity is the use of photoredox catalysis,^13–16^ which is able to efficiently generate the electrophilic chlorination reagent *in situ* while suppressing the detrimental Cl radical. The scope, application, and preliminary mechanistic study are described.1
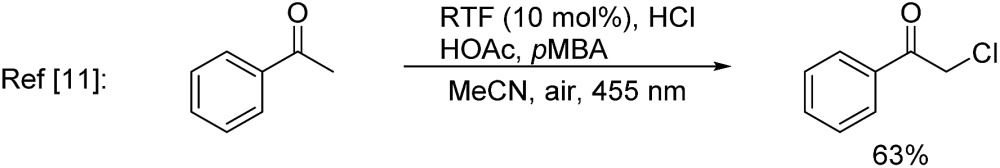

2
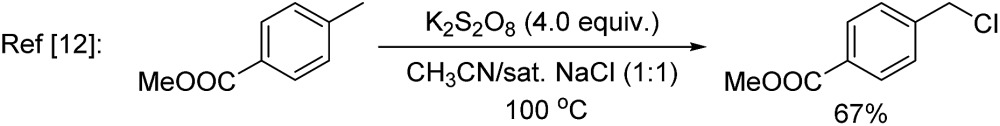

3
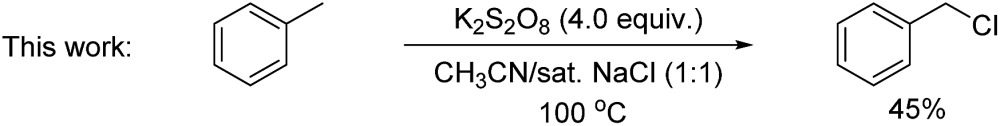



We commenced our study using toluene as the model substrate (Table 1). The selective chlorination of toluene is relatively challenging compared to other election-rich aromatic compounds. The methyl group is not a strong election-donating group and several side products such as benzaldehyde and chloromethylbenzene might form. We aimed for an “open-flask” method, so the optimizations were conducted under air (for details see Table S1, ESI†). To our delight, selective C(sp^2^–H) chlorination could be obtained at room temperature using Ru(bpy)_3_Cl_2_·6H_2_O **3** (3 mol%) as photocatalyst, Na_2_S_2_O_8_ (1.6 equiv.) as oxidant, NaCl (3.0 equiv.) as the chlorine source, and CH_3_CN/H_2_O (1 : 1) as solvent, yielding 56% 1-chloro-2-methyl-benzene **2a** and 34% 1-chloro-4-methyl-benzene **2a′** (entry 1). The use of other commonly used photocatalysts such as Ir[dFppy]_2_(dtbbpy)PF_6_ **4** (entry 2), eosin Y **5** (entry 3) and 9,10-anthracenedicarbonitrile **6** (entry 4) led to no chlorination yield. Control experiments showed that under identical conditions but in the absence of light (entry 5), Ru(bpy)Cl_2_·6H_2_O (entry 6), or Na_2_S_2_O_8_ (entry 7), no chlorination was obtained. It should be noted that no chlorination of the benzylic C–H bond was observed.

**Table 1:** Optimization of reaction conditions^*a*^

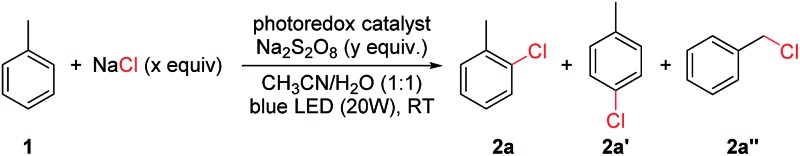						
Entry	Photocatalyst	NaCl (*x* equiv.)	Na_2_S_2_O_8_ (*y* equiv.)	Yield (%)		
				**2a**	**2a′**	**2a′′**
1	**3** (3 mol%)	3.0	1.6	56%	34%	0%
2	**4** (3 mol%)	3.0	1.6	0%	0%	0%
3	**5** (10 mol%)	3.0	1.6	0%	0%	0%
4	**6** (10 mol%)	3.0	1.6	0%	0%	0%
5^*b*^	**3** (3 mol%)	3.0	1.6	0%	0%	0%
6	0	3.0	1.6	0%	0%	0%
7	**3** (3 mol%)	3.0	0	0%	0%	0%
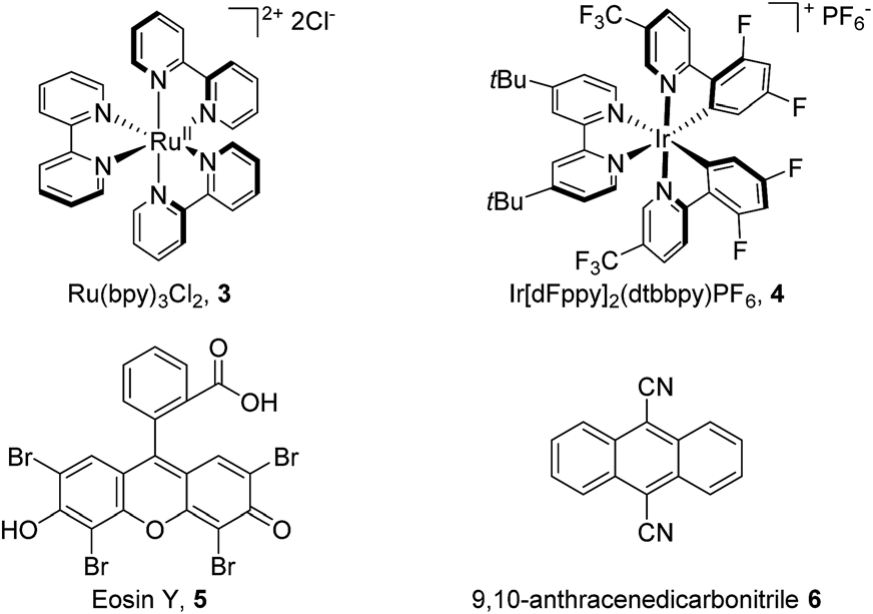						

^*a*^Reaction conditions: toluene (0.25 mmol) in CH_3_CN/H_2_O (1 mL) at 25 °C. Yields were obtained from the crude reaction mixture by GC relative to a mesitylene internal standard.

^*b*^Without light.

With the optimized conditions in hand, we studied the scope and limitation of this C–H chlorination method (Table 2). We first explored monosubstituted aromatic compounds. The substrates with an electron-donating group (isopropyl, **2b**; methoxy, **2c**) on the phenyl ring were chlorinated in good yields, and both *para*- and *ortho*-chlorinated products were formed. Substrates bearing functional groups such as a –CN (**2d**), an ester (**2e**), or a halogen (**2f** and **2g**) were all chlorinated in good yields as well. 1-(3-Bromopropyl)-4-chlorobenzene (**2g**) is an important building block for the synthesis of Parogrelil hydrochloride, a medication for the treatment of intermittent claudication. Previously^17^ it took four steps to prepare this compound, whereas using our method 1-(3-bromopropyl)-4-chlorobenzene (**2g**, 39%) and its isomer 1-(3-bromopropyl)-4-chlorobenzene (**2g′**, 33%) were prepared in one step. The two isomers were easily separated by column chromatography. In addition, aromatic compounds containing unprotected tertiary alcohol (**2h**) and amide (**2i** and **2j**) groups were suitable substrates. In agreement with the limitation of electrophilic chlorination, substrates containing only an election-withdrawing group such as nitrobenzene (**2k**) and (trifluoromethoxy)benzene (**2l**) could not be chlorinated by this method.

**Table 2:** Scope of C(sp^2^)–H chlorination^*a*^

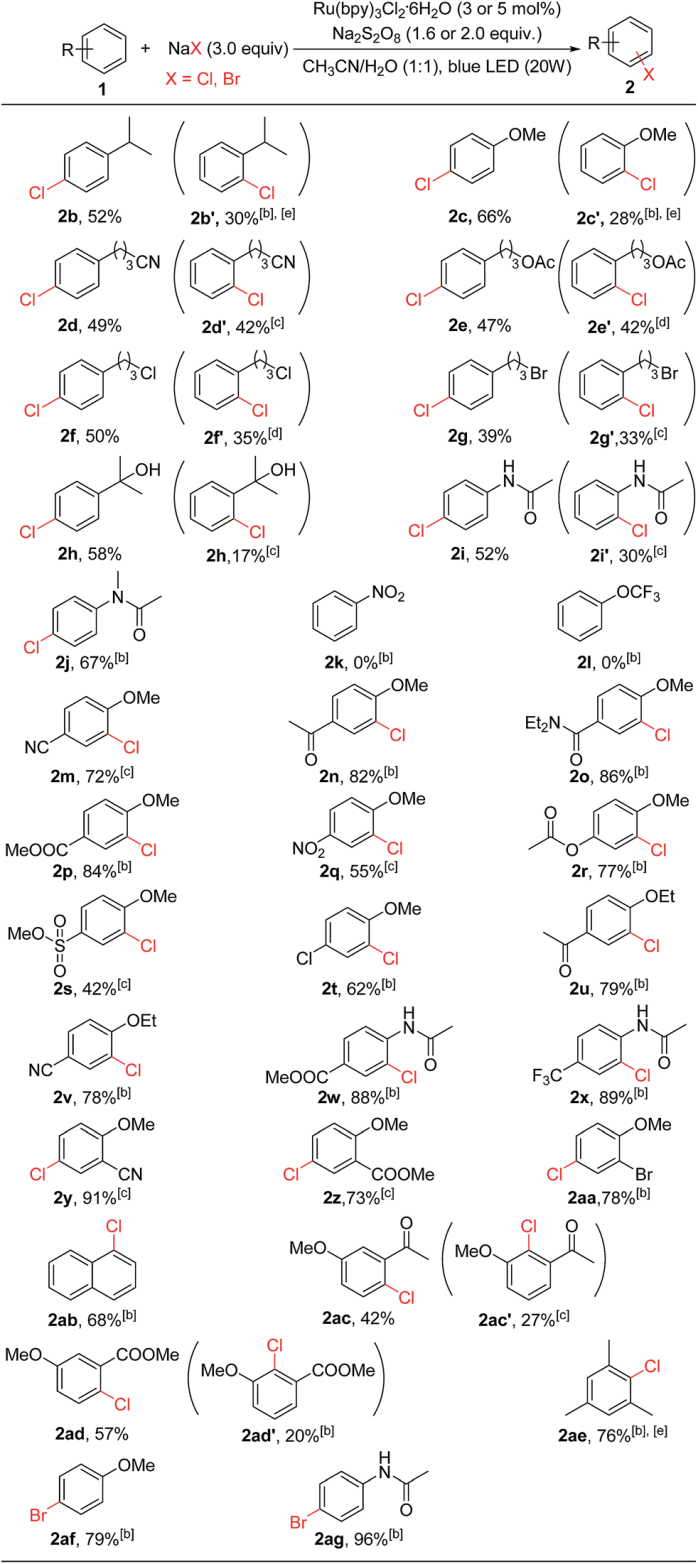

^*a*^Reaction conditions: substrate (0.5 mmol) in CH_3_CN/H_2_O (2 mL) at 25 °C. Unless otherwise specified, yields shown are isolated yields. Reaction time: 24 h.

^*b*^3 mol% of Ru(bpy)_3_Cl_2_·6H_2_O, 1.6 equiv. of Na_2_S_2_O_8_.

^*c*^5 mol% of Ru(bpy)_3_Cl_2_·6H_2_O, 2.0 equiv. of Na_2_S_2_O_8_.

^*d*^5 mol% of Ru(bpy)_3_Cl_2_·6H_2_O, 1.6 equiv. of Na_2_S_2_O_8_.

^*e*^Substrate (0.25 mmol) in CD_3_CN/H_2_O (1 mL). Yields were determined by ^1^H NMR relative to a mesitylene internal standard. Reaction time: 15 h.

However, substrates with election-withdrawing group(s) could be used if they also contain an election-donating group on the aryl ring, for example, an alkoxy group (**2m–2v**, **2y**, **2z**, **2aa**, **2ac**, or **2ad**). Thus, electron-withdrawing functional groups such as esters (**2p** and **2r**), trifluoromethyls (**2x**), amides (**2o**), halogens (**2t** and **2aa**), and sulfonates (**2s**) were all compatible. Even strong election-withdrawing groups such as cyano (**2m**) and nitro (**2q**) groups were tolerated. Although mono-substituted substrates are chlorinated at both the *ortho* and *para* positions due to the innate reactivity of Cl^+^ (see below), complete site selectivity could be achieved for disubstituted substrates bearing functional groups at either the 1,4 or 1,2 positions. In these cases chlorination occurred exclusively *ortho* to the electron-donating group. It is noted that exclusive C(sp^2^)–H chlorination also occurred for substrates containing an acyl group (**2n**, **2u** and **2ac**). Previous electrophilic chlorination methods normally led to α-carbonyl C(sp^3^)–H chlorination for acetophenone substrates.^11,12^ In addition to alkoxy groups, the amide group (**2w** and **2x**) could be used to promote the chlorination of electron-poor substrates. Chlorination of mesitylene gave the monochlorinated product in 76% yield (**2ae**). The photocatalytic oxidative halogenation strategy was also applicable to bromination. For example, bromination of anisole and *N*-phenylacetamide gave 1-bromo-4-methoxybenzene (**2ag**, 79%) and *N*-(4-bromophenyl)acetamide (**2ag**, 96%), respectively. However, iodination of anisole was not successful, possibly due to over-oxidation of iodine.

To demonstrate the potential utility of this oxidative chlorination method, it was applied for the expedited synthesis of some drugs and their key precursors. **8** is the precursor of glibenclamide, a medication used to treat type 2 diabetes. This compound could be obtained directly from **7** in 80% yield [eqn (4)]. Clofibrate (**10a**) is a lipid-lowering agent used for controlling high cholesterol and triacylglyceride levels in the blood. This compound could be obtained in one step using our C–H chlorination method from its precursor **9** in 57% yield, together with 28% of the dichlorination product [eqn (5)]. Chlorination of Boc-protected aminobenzene **11** gave **12a** (58%) and **12a′** (12%) [eqn (6)]. The major product **12a** is a precursor for the synthesis of several gastroprokinetic agents including metoclopramide, renzapride, and cisapride. Metoclopramide is one of the most widely used medicines for treating and preventing nausea and vomiting, and it is listed on the World Health Organization’s List of Essential Medicines.
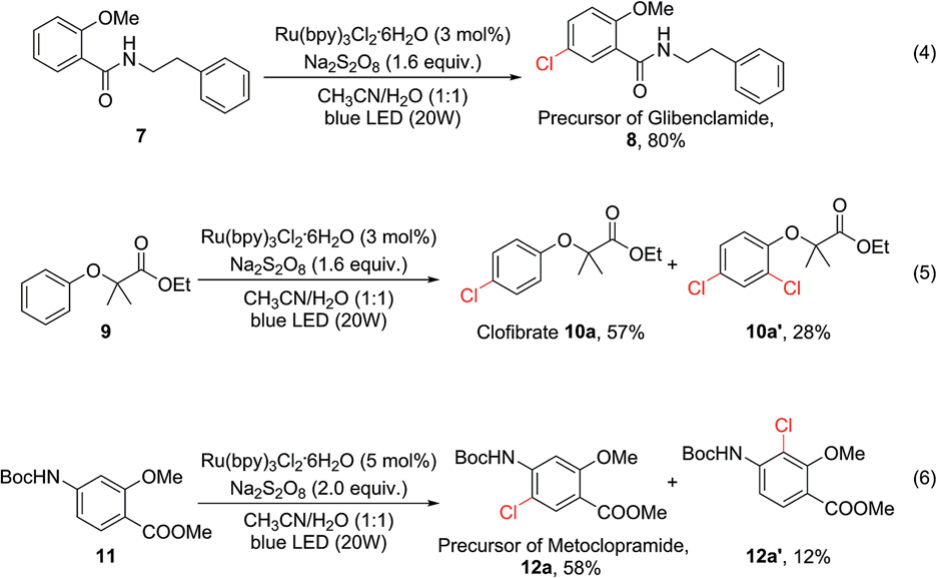


The mechanism of the reactions was then probed. It is well established that under light illumination, the excited *Ru(bpy)_3_^2+^ reacts with S_2_O_8_^2–^ to give Ru(bpy)_3_^3+^ and SO_4_·^–^, which further reacts with Ru(bpy)_3_^2+^ to give Ru(bpy)_3_^3+^ and SO_4_^2–^.^18^ Thus, either Ru(bpy)_3_^3+^ or SO_4_·^–^ or both might be the actual oxidant in this system. To probe these possibilities, Ru(bpy)_3_^3+^ was prepared chemically.^19^ With Ru(bpy)_3_^3+^ (3.2 equiv.; in accordance with the loading of Na_2_S_2_O_8_ in the catalytic system (1.6 equiv.)) as the sole oxidant, the reaction of toluene (1.0 equiv.) with NaCl (3.0 equiv.) gave 1-chloro-2-methyl-benzene (**2a**, 56%) and 1-chloro-4-methyl-benzene (**2a′**, 36%) in 30 min at room temperature (Scheme 2A). The faster rate of this reaction compared to catalysis reflects the higher loading of Ru(bpy)_3_^3+^ in the former. Thus, Ru(bpy)_3_^3+^ is a competent oxidant for the chlorination. Heating a solution of Na_2_S_2_O_8_ at 90 °C is known to generate SO_4_·^–^.^12^ The reaction of toluene (1.0 equiv.) with NaCl (3.0 equiv.) was then conducted in the presence of 1.6 equiv. of Na_2_S_2_O_8_ at 90 °C (Scheme 2B). However, no C(sp^2^)–H chlorination occurred; instead, a small amount of chloromethylbenzene (**2a′′**, 26%), the C(sp^3^–H) chlorination product, was formed (Scheme 2B). This result suggests that SO_4_·^–^ is not the major oxidant that reacts with the substrates in our system. To probe whether the chlorination reaction operates *via* a chain reaction mechanism after activation of S_2_O_8_^2–^ under the photocatalytic conditions, the chlorination of toluene was conducted under illumination for 40 minutes, and then the light was removed while allowing the reaction to continue for 15 further hours. Only 11% 1-chloro-2-methyl-benzene **2a** and 7% 1-chloro-4-methyl-benzene **2a′** were formed (Scheme 2C). The yields were much lower than those of the reaction conducted under illumination for the whole duration. This result indicates that a chain reaction based on activation of peroxodisulfate is unlikely.

**Scheme 2:**
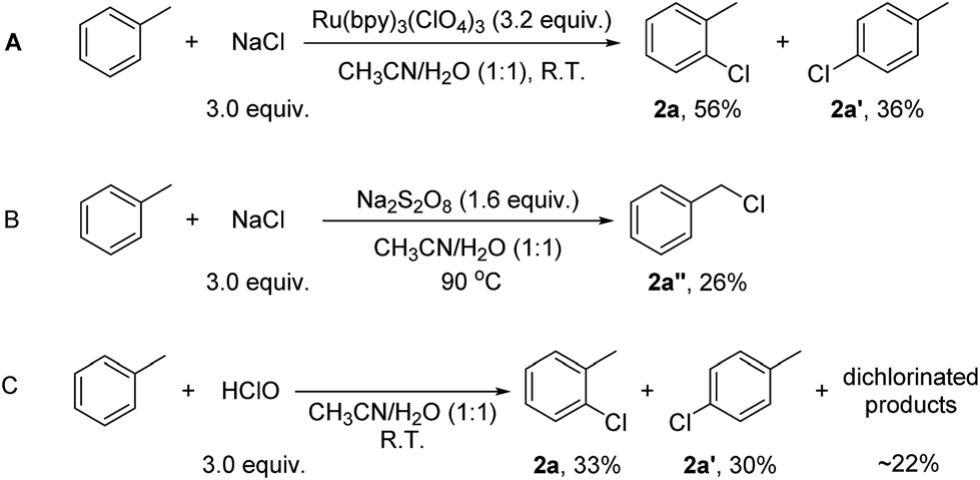
Experiments to probe the actual oxidant and chlorinating species in the current system.

Oxidative chlorination is proposed to occur *via* two major pathways.^12,20,21^ The first pathway (see ESI, Scheme S1,† Path A) involves the oxidation of chloride anions (Cl^–^) to chloride cations or their equivalent (“Cl^+^”),^22^ which react with aromatic compounds to give the chlorination products. The second pathway (Scheme S1,† Path B) involves the oxidation of aromatic compounds to aromatic radicals, which then react with chloride anions to give the chlorination products. According to its oxidative potential, Ru(bpy)_3_^3+^ (*E*III/II1/2 = +1.29 V *vs.* SCE) is not able to oxidize toluene (+2.28 V *vs.* SCE). To confirm this, the reaction of Ru(bpy)_3_^3+^ with toluene was monitored by UV-Vis spectroscopy. In CH_3_CN/H_2_O (1 : 1), Ru(bpy)_3_^3+^ on its own slowly decays into Ru(bpy)_3_^2+^ (Fig. S1, ESI†). When toluene (20 equiv.) was added to the solution containing Ru(bpy)_3_^3+^, the reduction rate remained constant (Fig. S2, ESI†). When NaCl (60 equiv.) was added to the solution, the reduction rate of Ru(bpy)_3_^3+^ became much faster (Fig. S3, ESI†). These results suggest that Ru(bpy)_3_^3+^ oxidizes Cl^–^ rather than the aromatic substrates in the catalysis. Due to the presence of water, the “Cl^+^” species might exist in the form of HClO, which works as the actual chlorinating species. To test this hypothesis, HClO was used as the chlorinating reagent for the chlorination of toluene. 1-Chloro-2-methyl-benzene (**2a**, 33%), 1-chloro-4-methyl-benzene (**2a′**, 30%) and some dichlorinated products (∼22%) were detected after 15 h (Scheme 2C). The product distribution is similar to, although not exactly the same as, that of the photocatalytic reaction. The dichlorinated products might originate from a high concentration of HClO in the stoichiometric reaction. Overall, the result in Scheme 2 suggests HClO as a probable chlorinating species in the photocatalytic reaction.

According to the above results, a tentative catalytic cycle is proposed in Fig. 1. Upon photoirradiation of Ru(bpy)_3_^2+^, *Ru(bpy)_3_^2+^ was formed.^23^ The reaction of *Ru(bpy)_3_^2+^ with Na_2_S_2_O_8_ gives Ru(bpy)_3_^3+^ and SO_4_·^–^. The latter oxidizes Ru(bpy)_3_^2+^ to give a further equivalent of Ru(bpy)_3_^3+^. Ru(bpy)_3_^3+^ then oxidizes Cl^–^ to Cl^+^ or its equivalent (HClO), probably *via* a Cl radical intermediate. When the chlorination of toluene was conducted in the presence of (2,2,6,6-tetramethylpiperidin-1-yl)oxyl (TEMPO, 3 equiv.), the chlorination was completely inhibited, supporting the involvement of a Cl radical. The further oxidation of the Cl radical must be very fast as no C(sp^3^–H) chlorination was observed. Finally, the chlorinating species reacts with an aromatic compound to effect the C(sp^2^–H) chlorination. This step likely occurs *via* an electrophilic addition process rather than an aromatic substitution process. To verify this, a 1 : 1 mixture of toluene and cyclohexene was subjected to the chlorination. After 1 h, 2-chlorocyclohexan-1-ol (∼20%) and 1,2-dichlorocyclohexane (2%) were generated while no chlorination of toluene was observed. This result supports the electrophilic addition pathway.

**Fig. 1:**
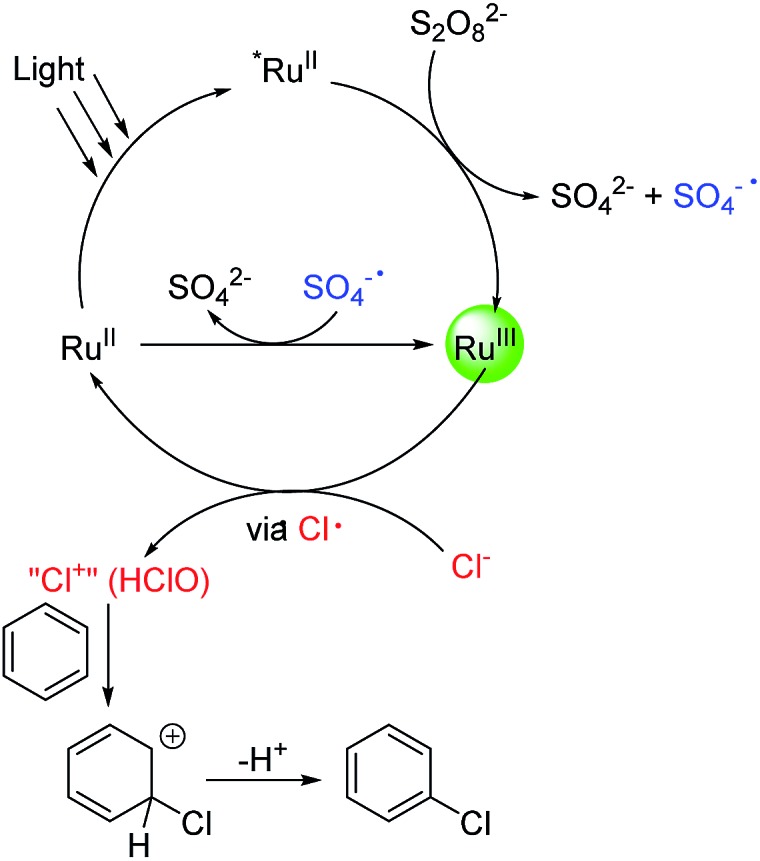
Proposed mechanism of the photocatalytic oxidative chlorination reaction.

## Conclusions

In conclusion, we have developed a photoredox catalytic method for C(sp^2^)–H oxidative chlorination at room temperature. Our method employs abundant and non-toxic NaCl as the chlorine source and inexpensive Na_2_S_2_O_8_ as the oxidant, and offers a practical and convenient alternative to existing electrophilic chlorination methods. The mild conditions lead to a broad scope and high functional group compatibility. The synthetic utility of this method is demonstrated in the chlorination of a diverse set of substrates, including the expedited synthesis of key intermediates to bioactive compounds and a drug.

## Conflicts of interest

There are no conflicts to declare.
