# Supplementary material for: Room temperature C(sp2)–H oxidative chlorination via photoredox catalysis
Source: Chem Sci. 2017 Aug 15;8(10):7009–13. doi: 10.1039/c7sc03010j (PMC5642143; doi:10.1039/c7sc03010j)
Supplement: Supplementary file 1 [file SC-008-C7SC03010J-s001.pdf]

Electronic Supplementary Material (ESI) for Chemical Science.
This journal is © The Royal Society of Chemistry 2017

Supporting Information

Room temperature C(sp^2)-H oxidative chlorination via photoredox catalysis

Lei Zhang and Xile Hu*

Laboratory of Inorganic Synthesis and Catalysis, Institute of Chemical Sciences and Engineering, Ecole Polytechnique Fédérale de Lausanne (EPFL), Lausanne CH-1015, Switzerland

E-mail: xile.hu@epfl.ch.

Table of Contents

1. General Information S3

2. Optimization of Reaction Conditions... S4

3. Preparation of Substrate S5

4. Chlorination of Aromatic Compounds S6

5. Synthesis of Clofibrate and Some Pharmaceutical Intermediates... S21

6. Mechanism study.. S24

7. References... S28

8. NMR Spectra S28

1. General Information

a. Materials

All manipulations were carried out under air. The following chemicals were purchased and used as received: $Ru(bpy)_3Cl_2{\cdot}6H_2O$ (Aldrich or TCI), $Na_2S_2O_8$ (Aldrich), aromatic compounds (Aldrich or TCI). $Ru(bpy)_3(ClO_4)_3$[1], substrates **1i**[2], **1j**[2], **1o**[3], **1s**[4], **1t**[5], **1x**[2], **7**[3] and **9**[6] were prepared according to previously reported procedures. All other reagents and solvents were purchased from commercial sources and used without purification.

b. Analytical Methods

NMR spectra were recorded on Bruker Avance 400 MHz spectrometers. ^{1}H NMR chemical shifts were referenced to residual protio solvent peaks or tetramethylsilane signal (0 ppm), and ^{13}C NMR chemical shifts were referenced to the solvent resonance. Data for ^{1}H NMR are recorded as follows: chemical shift (δ, ppm), multiplicity (s = singlet, d = doublet, t = triplet, m = multiplet or unresolved, coupling constant (s) in Hz, integration). Data for ^{13}C NMR are reported in terms of chemical shift (δ, ppm). GC measurements were conducted on a Perkin-Elmer Clarus 400 GC with a FID detector. GC-MS measurements were conducted on an Agilent Technologies 7890A GC system equipped with a 5975C MS detector. HRMS (ESI, APCI and EI) measurements were conducted at the EPFL ISIC Mass Spectrometry Service with a Micro Mass QTOF.

2. Optimization of Reaction Conditions

Table S1: Optimization of reaction conditions[a]

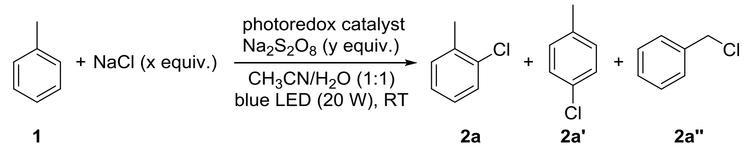

Entry	photo catalyst	NaCl (x equiv.)	$Na_2S_2O_8$ (y equiv.)	Yield (%)		
				**2a**	**2a'**	**2a"**
1	**3** (3 mol%)	3.0	1.6	56%	34%	0%
2	**4** (3 mol%)	3.0	1.6	0%	0%	0%
3	**5** (10 mol%)	3.0	1.6	0%	0%	0%
4	**6** (10 mol%)	3.0	1.6	0%	0%	0%
5	**3** (3 mol%)	3.0	1.2	46%	27%	0%
6	**3** (3 mol%)	3.0	1.4	49%	29%	0%
7	**3** (3 mol%)	3.0	1.8	56%	32%	0%
8	**3** (3 mol%)	3.0	2.0	54%	31%	0%
9	**3** (3 mol%)	1.5	1.6	40%	24%	0%
10	**3** (3 mol%)	2.0	1.6	47%	30%	0%
11	**3** (3 mol%)	2.5	1.6	50%	33%	0%
12	**3** (1 mol%)	3.0	1.6	29%	18%	0%
13	**3** (2 mol%)	3.0	1.6	48%	30%	0%
14[b]	**3** (3 mol%)	3.0	1.6	0%	0%	0%
15	0	3.0	1.6	0%	0%	0%
16	**3** (3 mol%)	3.0	0	0%	0%	0%

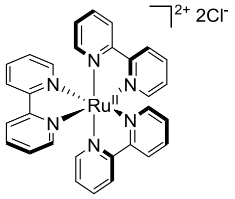

$Ru(bpy)_3Cl_2$, **3**

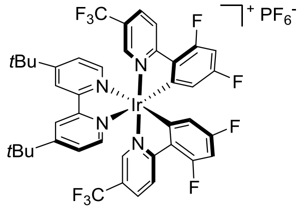

$Ir[dFppy]_2(dtbbpy)PF_6$, **4**

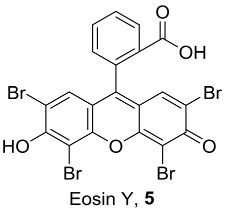

Eosin Y, **5**

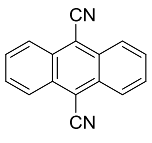

9,10-Anthracenedicarbonitrile, **6**

[a] Reaction conditions: Toluene (0.25 mmol) in CH_3CN/H_2O (1 mL) at 25 °C. Yields were obtained from the crude reaction mixture by GC relative to mesitylene internal standard. [b] Without light.

3. Preparation of Substrate

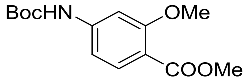

Methyl 4-((tert-butoxycarbonyl)amino)-2-methoxybenzoate (11) was prepared according to previously reported procedure.[7] White solid (1.22g, 72%). ^{1}H NMR (400 MHz, $CDCl_3$) δ 7.77 (d, *J* = 8.6 Hz, 1H, aryl-*H*), 7.37 (s, 1H, aryl-*H*), 6.90 (s, 1H, N*H*), 6.74 (dd, *J* = 8.5, 2.0 Hz, 1H, aryl-*H*), 3.88 (s, 3H), 3.84 (s, 3H), 1.49 (s, 9H, $C(CH_3)_3$). ^{13}C NMR (101 MHz, $CDCl_3$) δ 166.2, 160.9, 152.4 (aryl-*C*), 144.0 (aryl-*C*), 133.1 (aryl-*C*), 113.6 (aryl-*C*), 109.3 (aryl-*C*), 101.6 (aryl-*C*), 81.2 ($C(CH_3)_3$), 56.1 ($ArOCH_3$), 51.9 ($COOCH_3$), 28.4 ($C(CH_3)_3$). HRMS-ESI (m/z): Calcd for [($C_{14}H_{19}NO_5$+H)+], 282.1342 ; found: 282.1343.

4. Chlorination of Aromatic Compounds

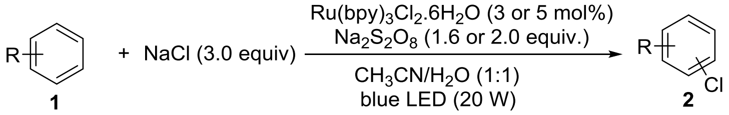

General procedure for chloronation of aromatic compounds: In the air, $Ru(bpy)_3Cl_2{\cdot}6H_2O$ (11.2 mg, 3 mol% or 18.7 mg 5 mol%), $Na_2S_2O_8$ (190 mg, 0.8 mmol, 1.6 equiv or 238 mg, 2.0 equiv), NaCl (88 mg, 1.5 mmol, 3 equiv), substrate (0.5 mmol) and solvent (CH_3CN/H_2O = 1/1, 2 mL) were added to a 8 mL vial equipped with a magnetic stir bar. The reaction was placed in the photoreactor and stirred at room temperature for 15~24 h. The resulting solution was extracted with ethyl acetate (3 mL x 3), after which the organic solution was combined and dried over Na_2SO_4. Then the solvent was evaporated under vacuum and the residue was purified by chromatography on silica gel, eluting with the mixture of ethyl acetate/hexane to give the corresponding products.

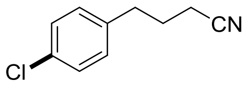

4-(4-chlorophenyl)butanenitrile (2d). 2d and **2d'** were synthesized following the general procedure. The residue was purified by chromatography on silica gel, eluting with hexane/ethyl acetate (20:1) to give the title compound as colorless oil (42.0 mg, 49%). ^{1}H NMR (400 MHz, $CDCl_3$) δ 7.30 (d, *J* = 8.5 Hz, 2H, aryl-*H*), 7.14 (d, *J* = 8.3 Hz, 2H, aryl-*H*), 2.77 (t, *J* = 7.5 Hz, 1H), 2.34 (t, *J* = 7.0 Hz, 1H), 1.97 (p, *J* = 7.2 Hz, 1H). ^{13}C NMR (101 MHz, $CDCl_3$) δ 138.2 (aryl-*C*), 132.4 (aryl-*C*), 129.9 (aryl-*C*), 128.9 (aryl-*C*), 119.4 (CN), 33.8, 26.8, 16.4. HRMS-NSI (*m/z*): Calcd for [($C_{10}H_{10}ClN$+H)+], 180.0575; found: 180.0570.

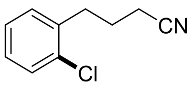

4-(2-chlorophenyl)butanenitrile (2d'). 2d and **2d'** were synthesized following the general procedure. The residue was purified by chromatography on silica gel, eluting

with hexane/ethyl acetate (20:1) to give the title compound as colorless oil (38.0 mg, 42%). ^{1}H NMR (400 MHz, $CDCl_3$) δ 7.36 (dd, *J* = 6.8, 2.0 Hz, 1H, aryl-*H*), 7.25 – 7.16 (m, 3H, aryl-*H*), 2.90 (t, *J* = 7.6 Hz, 2H), 2.36 (t, *J* = 7.1 Hz, 2H), 2.01 (m, 2H). ^{13}C NMR (101 MHz, $CDCl_3$) δ 137.6 (aryl-*C*), 134.1 (aryl-*C*), 130.7 (aryl-*C*), 129.9 (aryl-*C*), 128.2 (aryl-*C*), 127.2 (aryl-*C*), 119.5 (CN), 32.5, 25.4, 16.7. HRMS-NSI (*m/z*): Calcd for [($C_{10}H_{10}ClN$+H)+], 180.0575; found: 180.0569.

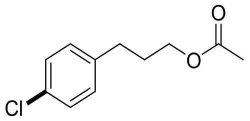

3-(2-chlorophenyl)propyl acetate (2e). 2e and **2e'** were synthesized following the general procedure. The residue was purified by chromatography on silica gel, eluting with hexane/ethyl acetate (30:1~10:1) to give the title compound as colorless oil (49.6 mg, 47%). ^{1}H NMR (400 MHz, $CDCl_3$) δ 7.25 (d, *J* = 7.8 Hz, 2H, aryl-*H*), 7.11 (d, *J* = 8.0 Hz, 2H, aryl-*H*), 4.07 (t, *J* = 6.5 Hz, 2H), 2.66 (t, *J* = 7.7 Hz, 2H), 2.05 (s, 3H, $COCH_3$), 1.97 – 1.88 (m, 2H). ^{13}C NMR (101 MHz, $CDCl_3$) δ 171.2 (*C*$OOCH_3$), 139.8 (aryl-*C*), 131.9 (aryl-*C*), 129.9 (aryl-*C*), 128.7 (aryl-*C*), 63.7, 31.7, 30.2, 21.1. These spectroscopic data correspond to reported data.[8]

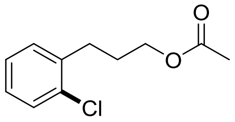

3-(4-chlorophenyl)propyl acetate (2e'). 2e and **2e'** were synthesized following the general procedure. The residue was purified by chromatography on silica gel, eluting with hexane/ethyl acetate (30:1~10:1) to give the title compound as colorless oil (45.0 mg, 42%). ^{1}H NMR (400 MHz, $CDCl_3$) δ 7.34 (dd, *J* = 7.7, 1.4 Hz, 1H, aryl-*H*), 7.23 – 7.11 (m, 3H, aryl-*H*), 4.10 (t, *J* = 6.5 Hz, 2H), 2.81 (t, *J* = 7.6 Hz, 2H), 2.06 (s, 3H, $COCH_3$), 2.01 – 1.92 (m, 2H). ^{13}C NMR (101 MHz, $CDCl_3$) δ 171.2 (*C*$OOCH_3$), 138.9 (aryl-*C*), 134.1 (aryl-*C*), 130.5 (aryl-*C*), 129.7 (aryl-*C*), 127.7 (aryl-*C*), 126.9 (aryl-*C*), 63.9 ($COOCH_3$), 30.2, 28.6, 21.1. HRMS-NSI (*m/z*): Calcd for

[($C_{11}H_{13}ClO_2$+Na)+], 235.0496; found: 235.0488.

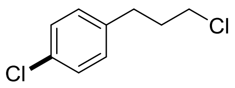

1-chloro-4-(3-chloropropyl)benzene (2f). 2f and **2f'** were synthesized following the general procedure. The residue was purified by chromatography on silica gel, eluting with hexane/ethyl acetate (50:1~30:1) to give the title compound as colorless oil Colorless oil (47.3 mg, 50%). ^{1}H NMR (400 MHz, $CDCl_3$) δ 7.27 (d, *J* = 8.4 Hz, 2H, aryl-*H*), 7.13 (d, *J* = 8.4 Hz, 2H, aryl-*H*), 3.52 (t, *J* = 6.4 Hz, 2H), 2.76 (t, *J* = 7.4 Hz, 2H), 2.10 – 2.02 (m, 2H). ^{13}C NMR (101 MHz, $CDCl_3$) δ 139.2 (aryl-*C*), 132.0 (aryl-*C*), 130.0 (aryl-*C*), 128.7 (aryl-*C*), 44.1, 34.0, 32.2. These spectroscopic data correspond to reported data.[9]

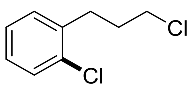

1-chloro-2-(3-chloropropyl)benzene (2f'). 2f and **2f'** were synthesized following the general procedure. The residue was purified by chromatography on silica gel, eluting with hexane/ethyl acetate (50:1~30:1) to give the title compound as colorless oil (47.3 mg, 50%). Colorless oil (33.0 mg, 35%). ^{1}H NMR (400 MHz, $CDCl_3$) δ 7.35 (dd, *J* = 7.6, 1.7 Hz, 1H, aryl-*H*), 7.24 – 7.13 (m, 3H, aryl-*H*), 3.56 (t, *J* = 6.5 Hz, 2H), 2.91 (t, *J* = 7.6 Hz, 2H), 2.15 – 2.07 (m, 2H). ^{13}C NMR (101 MHz, $CDCl_3$) δ 138.5 (aryl-*C*), 134.8 (aryl-*C*), 130.8 (aryl-*C*), 129.8 (aryl-*C*), 127.8 (aryl-*C*), 127.0 (aryl-*C*), 44.4, 32.4, 30.9.[9]

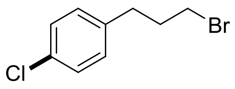

1-(3-bromopropyl)-4-chlorobenzene (2g). 2g and **2g'** were synthesized following the general procedure. The residue was purified by chromatography on silica gel, eluting with hexane/ethyl acetate (50:1~30:1) to give the title compound as colorless oil (45.0 mg, 39%). ^{1}H NMR (400 MHz, $CDCl_3$) δ 7.27 (d, *J* = 8.4 Hz, 2H, aryl-*H*),

7.14 (d, J = 8.4 Hz, 2H, aryl-H), 3.38 (t, J = 6.5 Hz, 1H), 2.76 (t, J = 7.4 Hz, 1H), 2.18 – 2.10 (m, 2H). ^{13}C NMR (101 MHz, $CDCl_3$) δ 139.1 (aryl-C), 132.1 (aryl-C), 130.0 (aryl-C), 128.7 (aryl-C), 34.1, 33.4, 32.9. These spectroscopic data correspond to reported data.[10]

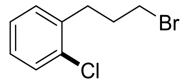

1-(3-bromopropyl)-2-chlorobenzene (2g'). **2g** and **2g'** were synthesized following the general procedure. The residue was purified by chromatography on silica gel, eluting with hexane/ethyl acetate (50:1~30:1) to give the title compound as colorless oil (39.1 mg, 33%). ^{1}H NMR (400 MHz, $CDCl_3$) δ 7.35 (dd, J = 7.5, 1.7 Hz, 1H, aryl-H), 7.26 (dd, J = 7.3, 2.0 Hz, 1H, aryl-H), 7.23 – 7.13 (m, 2H, aryl-H), 3.43 (t, J = 6.6 Hz, 2H), 2.90 (t, J = 7.4 Hz, 2H), 2.30 – 2.15 (m, 2H). ^{13}C NMR (101 MHz, $CDCl_3$) δ 138.3 (aryl-C), 134.1 (aryl-C), 130.8 (aryl-C), 129.8 (aryl-C), 127.9 (aryl-C), 127.0 (aryl-C), 33.2, 32.5, 32.2. These spectroscopic data correspond to reported data.[10]

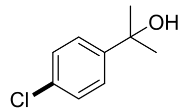

2-(4-chlorophenyl)propan-2-ol (2h). **2h** and **2h'** were synthesized following the general procedure. The residue was purified by chromatography on silica gel, eluting with hexane/ethyl acetate (20:1~5:1) to give the title compound as colorless oil (48.2 mg, 57%). ^{1}H NMR (400 MHz, $CDCl_3$) δ 7.41 (d, J = 8.6 Hz, 2H, aryl-H), 7.29 (d, J = 8.6 Hz, 2H, aryl-H), 1.94 (s, 1H, OH), 1.55 (s, 6H, CH(CH_3)$_3$). ^{13}C NMR (101 MHz, $CDCl_3$) δ 147.7 (aryl-C), 132.5 (aryl-C), 128.4 (aryl-C), 126.1 (aryl-C), 72.4 (CH(CH_3)$_3$), 31.9 (CH(CH$_3$)$_3$). These spectroscopic data correspond to reported data.[11]

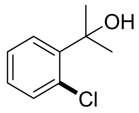

2-(2-chlorophenyl)propan-2-ol (2h'). **2h** and **2h'** were synthesized following the general procedure. The residue was purified by chromatography on silica gel, eluting with hexane/ethyl acetate (20:1~5:1) to give the title compound as colorless oil (15.2 mg, 18%). ^{1}H NMR (400 MHz, $CDCl_3$) δ 7.69 (dd, *J* = 7.8, 1.8 Hz, 1H, aryl-*H*), 7.38 (dd, *J* = 7.8, 1.5 Hz, 1H, aryl-*H*), 7.28 (td, *J* = 7.6, 1.5 Hz, 1H, aryl-*H*), 7.21 (td, *J* = 7.6, 1.8 Hz, 1H, aryl-*H*), 2.70 (s, 1H, O*H*), 1.76 (s, 6H, $CH(CH_3)_3$). ^{13}C NMR (101 MHz, $CDCl_3$) δ 144.7 (aryl-*C*), 131.4 (aryl-*C*), 131.3 (aryl-*C*), 128.3 (aryl-*C*), 127.0 (aryl-*C*), 126.9 (aryl-*C*), 73.1 ($CH(CH_3)_3$), 29.4 ($CH(CH_3)_3$). These spectroscopic data correspond to reported data.[12]

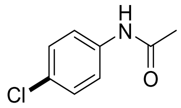

N-(4-chlorophenyl)acetamide (2i). **2i** and **2i'** were synthesized following the general procedure. The residue was purified by chromatography on silica gel, eluting with hexane/ethyl acetate (2:1~1:1) to give the title compound as white solid (43.7 mg, 52%). ^{1}H NMR (400 MHz, $CDCl_3$) δ 8.17 (s, 1H, N*H*), 7.29 (d, *J* = 8.9 Hz, 2H, aryl-*H*), 7.04 (d, *J* = 8.9 Hz, 2H, aryl-*H*), 1.79 (s, 3H, $COCH_3$). ^{13}C NMR (101 MHz, $CDCl_3$) δ 168.4 ($COCH_3$), 134.7 (aryl-*C*), 129.1 (aryl-*C*), 127.8 (aryl-*C*), 124.7 (aryl-*C*), 122.7 (aryl-*C*), 121.8 (aryl-*C*), 25.0 ($COCH_3$). These spectroscopic data correspond to reported data.[13]

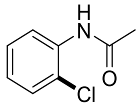

N-(2-chlorophenyl)acetamide (2i'). **2i** and **2i'** were synthesized following the general procedure. The residue was purified by chromatography on silica gel, eluting with hexane/ethyl acetate (2:1~1:1) to give the title compound as white solid (25.6 mg,

30%). ^{1}H NMR (400 MHz, CD_3CN) δ 8.36 (d, *J* = 8.3 Hz, 1H, aryl-*H*), 7.66 (s, 1H, N*H*), 7.37 (d, *J* = 7.9 Hz, 1H, aryl-*H*), 7.28 (t, *J* = 7.8 Hz, 1H, aryl-*H*), 7.05 (t, *J* = 7.5 Hz, 1H, aryl-*H*), 2.25 (s, 3H, COCH_3). ^{13}C NMR (101 MHz, CD_3CN) δ 170.2 (*C*OCH_3), 139.5 (aryl-*C*), 130.1 (aryl-*C*), 129.1 (aryl-*C*), 122.1 (aryl-*C*), 24.8 (CO*C*H_3). These spectroscopic data correspond to reported data.[7]

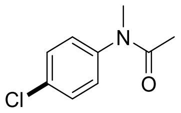

N-(4-chlorophenyl)-N-methylacetamide (2j). 2j was synthesized following the general procedure. The residue was purified by chromatography on silica gel, eluting with hexane/ethyl acetate (1:1) to give the title compound as white solid (62.0 mg, 67%). ^{1}H NMR (400 MHz, $CDCl_3$) δ 7.37 (d, *J* = 8.2 Hz, 1H, aryl-*H*), 7.12 (d, *J* = 8.2 Hz, 1H, aryl-*H*), 3.22 (s, 3H), 1.85 (s, 3H). ^{13}C NMR (101 MHz, $CDCl_3$) δ 170.3 (*C*OCH_3), 143.1 (aryl-*C*), 133.5 (aryl-*C*), 130.0 (aryl-*C*), 128.5 (aryl-*C*), 37.2, 22.4. These spectroscopic data correspond to reported data.[14]

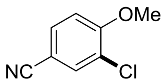

3-chloro-4-methoxybenzonitrile (2m). 2m was synthesized following the general procedure. The residue was purified by chromatography on silica gel, eluting with hexane/ethyl acetate (15:1~10:1) to give the title compound as white solid (60.0 mg, 72%). ^{1}H NMR (400 MHz, $CDCl_3$) δ 7.65 (d, *J* = 2.1 Hz, 1H, aryl-*H*), 7.55 (dd, *J* = 8.6, 2.1 Hz, 1H, aryl-*H*), 6.98 (d, *J* 8.6 Hz, 1H, aryl-*H*), 3.96 (s, 3H, ArOCH_3). ^{13}C NMR (101 MHz, $CDCl_3$) δ 158.6 (aryl-*C*), 133.6 (aryl-*C*), 132.5 (aryl-*C*), 123.6 (aryl-*C*), 117.9 (aryl-*C*), 112.3 (aryl-*C*), 104.8 (Ar*C*N), 56.5 (ArO*C*H_3). These spectroscopic data correspond to reported data.[15]

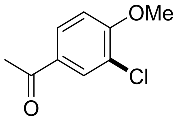

1-(3-chloro-4-methoxyphenyl)ethan-1-one (2n). 2n was synthesized following the general procedure. The residue was purified by chromatography on silica gel, eluting with hexane/ethyl acetate (20:1) to give the title compound as white solid (75.6 mg, 82%). ^{1}H NMR (400 MHz, $CDCl_3$) δ 7.92 (d, *J* = 2.2 Hz, 1H, aryl-*H*), 7.80 (dd, *J* = 8.6, 2.2 Hz, 1H, aryl-*H*), 6.91 (d, *J* = 8.6 Hz, 1H, aryl-*H*), 3.92 (s, 3H, ArOCH_3), 2.50 (s, 3H, COCH_3). ^{13}C NMR (101 MHz, $CDCl_3$) δ 195.7 (*C*OCH_3), 158.7 (aryl-*C*), 130.8 (aryl-*C*), 130.6 (aryl-*C*), 128.8 (aryl-*C*), 122.8 (aryl-*C*), 111.3 (aryl-*C*), 56.4 (ArO*C*H_3), 26.3 (CO*C*H_3). These spectroscopic data correspond to reported data.[16]

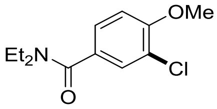

3-chloro-N,N-diethyl-4-methoxybenzamide (2o). 2o was synthesized following the general procedure. The residue was purified by chromatography on silica gel, eluting with hexane/ethyl acetate (3:1~2:1) to give the title compound as colorless oil (104.2 mg, 86%). ^{1}H NMR (400 MHz, $CDCl_3$) δ 7.38 (d, *J* = 2.1 Hz, 1H, aryl-*H*), 7.23 (dd, *J* = 8.4, 2.1 Hz, 1H, aryl-*H*), 6.89 (d, *J* = 8.4 Hz, 1H, aryl-*H*), 3.87 (s, 3H, ArOCH_3), 3.37 (br, 4H, NCH_2CH_3), 1.13 (br, 6H, NCH_2CH_3). ^{13}C NMR (101 MHz, $CDCl_3$) δ 169.7 (*C*ONEt_2), 155.7 (aryl-*C*), 130.3 (aryl-*C*), 128.7 (aryl-*C*), 126.3 (aryl-*C*), 122.4 (aryl-*C*), 111.7 (aryl-*C*), 56.2 (ArO*C*H_3), 43.3 (N*C*H_2CH_3), 39.6 (N*C*H_2CH_3), 14.1 (NCH_2*C*H_3), 12.9 (NCH_2*C*H_3). HRMS-ESI (m/z): Calcd for [($C_{12}H_{16}ClNO_2$+H)+], 242.0948; found: 242.0952.

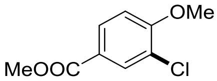

Methyl 3-chloro-4-methoxybenzoate (2p). 2p was synthesized following the general procedure. The residue was purified by chromatography on silica gel, eluting with

hexane/ethyl acetate (10:1) to give the title compound as white solid (84.5 mg, 84%). ^{1}H NMR (400 MHz, $CDCl_3$) δ 8.05 (d, *J* = 2.1 Hz, 1H, aryl-*H*), 7.93 (dd, *J* = 8.6, 2.1 Hz, 1H, aryl-*H*), 6.94 (d, *J* = 8.6 Hz, 1H, aryl-*H*), 3.96 (s, 3H, COOC*H*$_3$), 3.89 (s, 3H, ArOC*H*$_3$). ^{13}C NMR (101 MHz, $CDCl_3$) δ 165.8 (*C*OOCH$_3$), 158.6 (aryl-*C*), 131.6 (aryl-*C*), 129.9 (aryl-*C*), 123.3 (aryl-*C*), 122.5 (aryl-*C*), 111.2 (aryl-*C*), 56.3 (ArO*C*H$_3$), 52.2 (COO*C*H$_3$). These spectroscopic data correspond to reported data.[17]

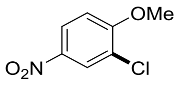

2-chloro-1-methoxy-4-nitrobenzene (2q). 2q was synthesized following the general procedure. The residue was purified by chromatography on silica gel, eluting with hexane/ethyl acetate (20:1~10:1) to give the title compound as white solid (51.0 mg, 55%). ^{1}H NMR (400 MHz, $CDCl_3$) δ 8.05 (d, *J* = 2.1 Hz, 1H, aryl-*H*), 7.93 (dd, *J* = 8.6, 2.1 Hz, 1H, aryl-*H*), 6.94 (d, *J* = 8.6 Hz, 1H, aryl-*H*), 3.96 (s, 3H), 3.89 (s, 3H). ^{13}C NMR (101 MHz, $CDCl_3$) δ 160.2 (aryl-*C*), 141.3 (aryl-*C*), 126.0 (aryl-*C*), 124.1 (aryl-*C*), 123.3 (aryl-*C*), 111.1 (aryl-*C*), 57.0 (ArO*C*H$_3$). These spectroscopic data correspond to reported data.[18]

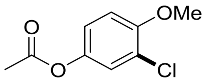

3-chloro-4-methoxyphenyl acetate (2r). 2r was synthesized following the general procedure. The residue was purified by chromatography on silica gel, eluting with hexane/ethyl acetate (20:1~10:1) to give the title compound as yellow oil (77.1 mg, 77%). ^{1}H NMR (400 MHz, $CDCl_3$) δ 7.14 (d, *J* = 2.7 Hz, 1H, aryl-*H*), 6.96 (dd, *J* = 8.9, 2.7 Hz, 1H, aryl-*H*), 6.88 (d, *J* = 8.9 Hz, 1H, aryl-*H*), 3.86 (s, 3H, OCOC*H*$_3$), 2.25 (s, 3H, ArOC*H*$_3$). ^{13}C NMR (101 MHz, $CDCl_3$) δ 169.5 (O*C*OCH$_3$), 153.0 (aryl-*C*), 143.9 (aryl-*C*), 123.7 (aryl-*C*), 122.6 (aryl-*C*), 120.7 (aryl-*C*), 112.1 (aryl-*C*), 56.5 (ArO*C*H$_3$), 21.0 (OCO*C*H$_3$). HRMS-APCI (m/z): Calcd for [($C_9H_9ClO_3$+H)+], 201.0313; found: 201.0314.

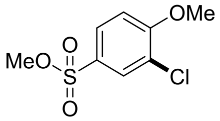

Methyl 3-chloro-4-methoxybenzenesulfonate (2s). 2s was synthesized following the general procedure. The residue was purified by chromatography on silica gel, eluting with hexane/ethyl acetate (10:1~5:1) to give the title compound as pale yellow oil (50.0 mg, 42%). ^{1}H NMR (400 MHz, $CDCl_3$) δ 7.89 (d, *J* = 2.3 Hz, 1H, aryl-*H*), 7.79 (dd, *J* = 8.7, 2.3 Hz, 1H, aryl-*H*), 7.04 (d, *J* = 8.7 Hz, 1H, aryl-*H*), 3.98 (s, 3H), 3.98 (s, 3H). ^{13}C NMR (101 MHz, $CDCl_3$) δ 159.4 (aryl-*C*), 130.1 (aryl-*C*), 128.6 (aryl-*C*), 127.4 (aryl-*C*), 123.7 (aryl-*C*), 111.9 (aryl-*C*), 56.7，56.5. HRMS-NSI (*m/z*): Calcd for [($C_8H_9ClO_4S$+Na)+], 258.9802; found: 258.9795.

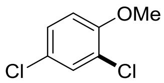

2,4-dichloro-1-methoxybenzene (2t). 2t was synthesized following the general procedure. The residue was purified by chromatography on silica gel, eluting with hexane/ethyl acetate (30:1) to give the title compound as pale yellow oil (55.0 mg, 62%). ^{1}H NMR (400 MHz, $CDCl_3$) δ 7.36 (d, *J* = 2.6 Hz, 1H, aryl-*H*), 7.19 (dd, *J* = 8.8, 2.6 Hz, 1H, aryl-*H*), 6.84 (d, *J* = 8.8 Hz, 1H, aryl-*H*), 3.88 (s, 3H, ArOC*H*$_3$). ^{13}C NMR (101 MHz, $CDCl_3$) δ 154.0 (aryl-*C*), 130.1 (aryl-*C*), 127.7 (aryl-*C*), 125.8 (aryl-*C*), 123.4 (aryl-*C*), 112.9 (aryl-*C*), 56.5 (ArO*C*H$_3$). These spectroscopic data correspond to reported data.[19]

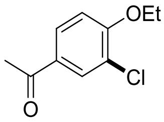

1-(3-chloro-4-ethoxyphenyl)ethan-1-one (2u). 2u was synthesized following the general procedure. The residue was purified by chromatography on silica gel, eluting with hexane/ethyl acetate (10:1) to give the title compound as pale yellow solid (78.7 mg, 79%). ^{1}H NMR (400 MHz, $CDCl_3$) δ 7.95 (d, *J* = 2.2 Hz, 1H, aryl-*H*), 7.80 (dd,

J = 8.6, 2.2 Hz, 1H, aryl-H), 6.90 (d, J = 8.6 Hz, 1H, aryl-H), 4.15 (q, J = 7.0 Hz, 2H, ArOCH_2CH$_3$), 2.51 (s, 3H, COCH_3), 1.47 (t, J = 7.0 Hz, 3H, ArOCH$_2$CH_3). ^{13}C NMR (101 MHz, CDCl$_3$) δ 195.8 (COCH$_3$), 158.3 (aryl-C), 130.7 (aryl-C), 130.6 (aryl-C), 128.8 (aryl-C), 123.0 (aryl-C), 112.1 (aryl-C), 65.0 (ArOCH$_2$CH$_3$), 26.3 (COCH$_3$), 14.6 (ArOCH$_2$$CH_3$). HRMS-ESI (m/z): Calcd for [($C_{10}H_{11}ClO_2$+H)+], 199.0526; found: 199.0527.

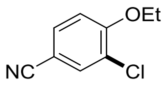

3-chloro-4-ethoxybenzonitrile (2v). 2j was synthesized following the general procedure. The residue was purified by chromatography on silica gel, eluting with hexane/ethyl acetate (30:1~10:1) to give the title compound as white solid (71.2 mg, 78%). ^{1}H NMR (400 MHz, CDCl$_3$) δ 7.60 (d, J = 2.1 Hz, 1H, aryl-H), 7.49 (dd, J = 8.6, 2.1 Hz, 1H, aryl-H), 6.93 (d, J = 8.6 Hz, 1H, aryl-H), 4.14 (q, J = 7.0 Hz, 2H, ArOCH_2CH$_3$), 1.48 (t, J = 7.0 Hz, 3H, ArOCH$_2$CH_3). ^{13}C NMR (101 MHz, CDCl$_3$) δ 158.1 (aryl-C), 133.6 (aryl-C), 132.4 (aryl-C), 123.7 (aryl-C), 118.0 (aryl-C), 113.0 (aryl-C), 104.3(CN), 65.2 (ArOCH$_2$CH$_3$), 14.4 (ArOCH$_2$$CH_3$). HRMS-APCI (m/z): Calcd for [(C_9H_8ClNO+H)+], 182.0367; found: 182.0370.

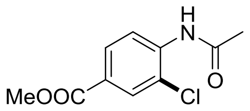

Methyl 4-acetamido-3-chlorobenzoate (2w). 2w was synthesized following the general procedure. The residue was purified by chromatography on silica gel, eluting with hexane/ethyl acetate (3:1) to give the title compound as white solid (100.3 mg, 88%). ^{1}H NMR (400 MHz, CDCl$_3$) δ 8.48 (d, J = 8.7 Hz, 1H, aryl-H), 8.02 (d, J = 1.9 Hz, 1H, aryl-H), 7.90 (dd, J = 8.7, 1.9 Hz, 1H, aryl-H), 7.81 (s, 1H, NH), 3.88 (s, 3H, COOCH_3), 2.25 (s, 3H, NHCOCH_3). ^{13}C NMR (101 MHz, CDCl$_3$) δ 168.5, 165.5, 138.6 (aryl-C), 130.3 (aryl-C), 129.2 (aryl-C), 126.0 (aryl-C), 122.0 (aryl-C),

120.5 (aryl-*C*), 52.3 (COO*C*H_3), 24.9 (NHCO*C*H_3). These spectroscopic data correspond to reported data.[20]

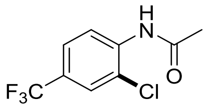

N-(2-chloro-4-(trifluoromethyl)phenyl)acetamide (2x). 2x was synthesized following the general procedure. The residue was purified by chromatography on silica gel, eluting with hexane/ethyl acetate (3:1) to give the title compound as white solid (105.1 mg, 89%). ^{1}H NMR (400 MHz, $CDCl_3$) δ 8.53 (d, *J* = 8.7 Hz, 1H, aryl-*H*), 7.78 (s, N*H*), 7.61 (s, 1H, aryl-*H*), 7.49 (d, *J* = 8.6 Hz, 1H, aryl-*H*), 2.26 (s, 3H, COC*H*$_3$). ^{13}C NMR (101 MHz, $CDCl_3$) δ 168.6 (*C*=O), 137.8 (aryl-*C*), 126.5 (q, *J* = 33.5 Hz, aryl-*C*), 126.2 (q, *J* = 4.1 Hz, aryl-*C*), 125.0 (q, *J* = 3.7 Hz, aryl-*C*), 123.4 (q, *J* = 271.9 Hz, *C*F_3), 122.4 (aryl-*C*), 121.2 (aryl-*C*), 25.0 (CO*C*H_3). ^{19}F NMR (376 MHz, $CDCl_3$) δ = -62.3. These spectroscopic data correspond to reported data.[21]

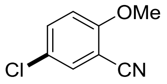

5-chloro-2-methoxybenzonitrile (2y). 2y was synthesized following the general procedure. The residue was purified by chromatography on silica gel, eluting with hexane/ethyl acetate (10:1~5:1) to give the title compound as white solid (76.6 mg, 91%). ^{1}H NMR (400 MHz, $CDCl_3$) δ 7.53 – 7.47 (m, 2H, aryl-H), 6.92 (d, *J* = 8.9 Hz, 1H, aryl-*H*), 3.93 (s, 3H, ArOC*H*$_3$). ^{13}C NMR (101 MHz, $CDCl_3$) δ 159.9 (aryl-*C*), 134.4 (aryl-*C*), 132.9 (aryl-*C*), 125.6 (aryl-*C*), 115.1 (aryl-*C*), 112.8 (aryl-*C*), 103.0 (Ar*C*N), 56.5 (ArO*C*H_3). These spectroscopic data correspond to reported data.[22]

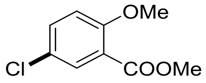

Methyl 5-chloro-2-methoxybenzoate (2z). 2z was synthesized following the general

procedure. The residue was purified by chromatography on silica gel, eluting with hexane/ethyl acetate (10:1~7:1) to give the title compound as pale yellow oil (72.9 mg, 73%). ^{1}H NMR (400 MHz, $CDCl_3$) δ 7.74 (d, *J* = 2.8 Hz, 1H, aryl-*H*), 7.39 (dd, *J* = 8.9, 2.8 Hz, 1H, aryl-*H*), 6.90 (d, *J* = 8.9 Hz, 1H, aryl-*H*), 3.87 (s, 3H, COOC*H*$_3$), 3.87 (s, 3H, ArOC*H*$_3$). ^{13}C NMR (101 MHz, $CDCl_3$) δ 165.4 (*C*OOCH$_3$), 157.8 (aryl-*C*), 133.1 (aryl-*C*), 131.3 (aryl-*C*), 125.2 (aryl-*C*), 121.3 (aryl-*C*), 113.5 (aryl-*C*), 56.3 (ArO*C*H$_3$), 52.2 (COO*C*H$_3$). These spectroscopic data correspond to reported data.[23]

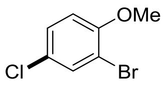

2-bromo-4-chloro-1-methoxybenzene (2aa). 2aa was synthesized following the general procedure. The residue was purified by chromatography on silica gel, eluting with hexane/ethyl acetate (20:1) to give the title compound as pale yellow oil (86.0 mg, 78%). ^{1}H NMR (400 MHz, $CDCl_3$) δ 7.52 (d, *J* = 2.6 Hz, 1H, aryl-*H*), 7.23 (dd, *J* = 8.8, 2.5 Hz, 1H, aryl-*H*), 6.80 (d, *J* = 8.8 Hz, 1H, aryl-*H*), 3.87 (s, 3H, ArOC*H*$_3$). ^{13}C NMR (101 MHz, $CDCl_3$) δ 154.9 (aryl-*C*), 132.9 (aryl-*C*), 128.4 (aryl-*C*), 126.1 (aryl-*C*), 112.7 (aryl-*C*), 112.2 (aryl-*C*), 56.6 (ArO*C*H$_3$). These spectroscopic data correspond to reported data.[24]

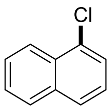

1-chloronaphthalene (2ab). 2ab was synthesized following the general procedure. The residue was purified by chromatography on silica gel, eluting with hexane/ethyl acetate (30:1) to give the title compound as colorless oil (55.0 mg, 68%). ^{1}H NMR (400 MHz, $CDCl_3$) δ 8.30 (d, *J* = 8.4 Hz, 1H, aryl-*H*), 7.87 (d, *J* = 8.1 Hz, 1H, aryl-*H*), 7.78 (d, *J* = 8.2 Hz, 1H, aryl-*H*), 7.65 – 7.53 (m, 3H, aryl-*H*), 7.40 (t, *J* = 7.8 Hz, 1H, aryl-*H*). ^{13}C NMR (101 MHz, $CDCl_3$) δ 134.7 (aryl-*C*), 132.0 (aryl-*C*), 130.9 (aryl-*C*), 128.3 (aryl-*C*), 127.3 (aryl-*C*), 127.2 (aryl-*C*), 126.8 (aryl-*C*), 126.3 (aryl-*C*),

125.8 (aryl-*C*), 124.5 (aryl-*C*). These spectroscopic data correspond to reported data.[25]

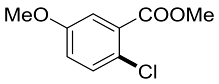

Methyl 2-chloro-5-methoxybenzoate (2ac). 2ac and **2ac'** was synthesized following the general procedure. The residue was purified by chromatography on silica gel, eluting with hexane/ethyl acetate (20:1~15:1) to give the title compound as colorless oil (57.3 mg, 57%). ^{1}H NMR (400 MHz, $CDCl_3$) δ 7.36 – 7.32 (m, 2H, aryl-*H*), 6.97 (dd, *J* = 8.9, 3.1 Hz, 1H, aryl-*H*), 3.94 (s, 3H), 3.83 (s, 3H). ^{13}C NMR (101 MHz, $CDCl_3$) δ 166.1 (*C*$OOCH_3$), 158.0 (aryl-*C*), 131.9 (aryl-*C*), 130.7 (aryl-*C*), 125.0 (aryl-*C*), 119.0 (aryl-*C*), 116.1 (aryl-*C*), 55.8, 52.6. These spectroscopic data correspond to reported data.[26]

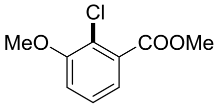

Methyl 4-chloro-3-methoxybenzoate (2ac'). 2ac and **2ac'** was synthesized following the general procedure. The residue was purified by chromatography on silica gel, eluting with hexane/ethyl acetate (20:1~15:1) to give the title compound as colorless oil (19.7 mg, 20%). ^{1}H NMR (400 MHz, $CDCl_3$) δ 7.33 (dd, J = 7.8, 1.6 Hz, 1H, aryl-*H*), 7.26 (t, *J* = 7.9 Hz, 1H, aryl-*H*), 7.05 (dd, *J* = 8.1, 1.6 Hz, 1H, aryl-*H*), 3.92 (s, 3H), 3.91 (s, 3H). ^{13}C NMR (101 MHz, $CDCl_3$) δ 166.6 (*C*$OOCH_3$), 155.8 (aryl-*C*), 132.3 (aryl-*C*), 127.2 (aryl-*C*), 122.5 (aryl-*C*), 122.1 (aryl-*C*), 114.7 (aryl-*C*), 56.7, 52.6. These spectroscopic data correspond to reported data.[27]

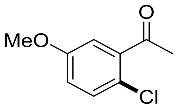

1-(2-chloro-5-methoxyphenyl)ethan-1-one (2ad). 2ad and **2ad'** was synthesized following the general procedure. The residue was purified by chromatography on

silica gel, eluting with hexane/ethyl acetate (30:1) to give the title compound as colorless oil (38.4 mg, 42%). ^{1}H NMR (400 MHz, $CDCl_3$) δ 7.28 (d, *J* = 8.8 Hz, 1H, aryl-*H*), 7.04 (d, *J* = 3.1 Hz, 1H, aryl-*H*), 6.91 (dd, *J* = 8.8, 3.1 Hz, 1H, aryl-*H*), 3.79 (s, 3H, ArOC*H*$_3$), 2.63 (s, 3H, COC*H*$_3$). ^{13}C NMR (101 MHz, $CDCl_3$) δ 200.4 (*C*OCH$_3$), 158.4 (aryl-*C*), 139.8 (aryl-*C*), 131.6 (aryl-*C*), 122.7 (aryl-*C*), 118.3 (aryl-*C*), 114.2 (aryl-*C*), 55.8 (ArO*C*H$_3$), 30.8 (CO*C*H$_3$). HRMS-NSI (*m/z*): Calcd for [($C_9H_9ClO_2$+H)+], 185.0364; found: 185.0358.

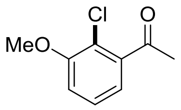

1-(2-chloro-3-methoxyphenyl)ethan-1-one (2ad'). **2ad** and **2ad'** was synthesized following the general procedure. The residue was purified by chromatography on silica gel, eluting with hexane/ethyl acetate (30:1) to give the title compound as colorless oil (25.0 mg, 27%). ^{1}H NMR (400 MHz, $CDCl_3$) δ 7.27 (t, *J* = 7.9 Hz, 1H, aryl-*H*), 7.04 (dd, *J* = 7.8, 1.4 Hz, aryl-*H*), 7.01 (dd, *J* = 8.2, 1.4 Hz, 1H, aryl-*H*), 3.92 (s, 3H, ArOC*H*$_3$), 2.62 (s, 3H, COC*H*$_3$). ^{13}C NMR (101 MHz, $CDCl_3$) δ 201.2 (*C*OCH$_3$), 155.5 (aryl-*C*), 141.5 (aryl-*C*), 127.8 (aryl-*C*), 120.3 (aryl-*C*), 119.5 (aryl-*C*), 113.9 (aryl-*C*), 56.6 (ArO*C*H$_3$), 31.0 (CO*C*H$_3$). HRMS-NSI (*m/z*): Calcd for [($C_9H_9ClO_2$+H)+], 185.0364; found: 185.0358.

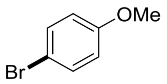

1-bromo-4-methoxybenzene (2af). **2af** was synthesized following the general procedure. The residue was purified by chromatography on silica gel, eluting with hexane/ethyl acetate (10:1) to give the title compound as yellow oil (73.7 mg, 79%). ^{1}H NMR (400 MHz, $CDCl_3$) δ 7.38 (d, *J* = 8.9 Hz, 1H, aryl-*H*), 6.78 (d, *J* = 8.9 Hz, 1H, aryl-*H*), 3.78 (s, 2H, ArOC*H*$_3$). ^{13}C NMR (101 MHz, $CDCl_3$) δ 158.8 (aryl-*C*), 132.3 (aryl-*C*), 115.8 (aryl-*C*), 112.9 (aryl-*C*), 55.5 (ArO*C*H$_3$). These spectroscopic data correspond to reported data.[28]

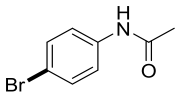

N-(4-bromophenyl)acetamide (2ag). 2ag was synthesized following the general procedure. The residue was purified by chromatography on silica gel, eluting with hexane/ethyl acetate (1:1) to give the title compound white solid (103.1 mg, 96%). ^{1}H NMR (400 MHz, $CDCl_3$) δ 7.67 (bs, 1H, N*H*), 7.39 (s, 4H, aryl-*H*), 2.15 (s, 3H, $COCH_3$). ^{13}C NMR (101 MHz, $CDCl_3$) δ 168.9 (*C*OCH_3), 137.1 (aryl-*C*), 132.0 (aryl-*C*), 121.7 (aryl-*C*), 117.0 (aryl-*C*), 24.6 (CO*C*H_3). These spectroscopic data correspond to reported data.[13]

5. Synthesis of Clofibrate and Some Pharmaceutical Intermediates

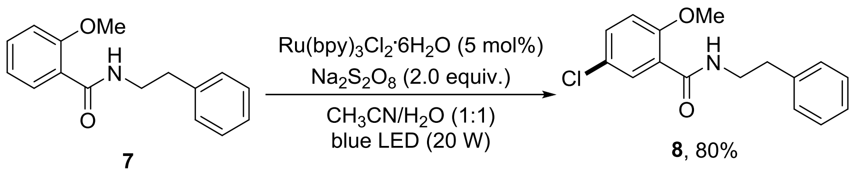

7 **8**, 80%

5-chloro-2-methoxy-N-phenethylbenzamide (8). 8 was synthesized following the general procedure of chlorination. The residue was purified by chromatography on silica gel, eluting with hexane/ethyl acetate (5:1~3:1) to give the title compound as colorless oil (115.6 mg, 80%). ^{1}H NMR (400 MHz, $CDCl_3$) δ 8.16 (d, *J* = 2.8 Hz, 1H, aryl-*H*), 7.85 (s, 1H, aryl-*H*), 7.37 – 7.22 (m, 6H, aryl-*H*), 6.82 (d, *J* = 8.8 Hz, 1H, aryl-*H*), 3.80 – 3.69 (m, 5H, OCH_3 and $NHCH_2$), 2.92 (t, *J* = 6.8 Hz, 2H, $PhCH_2$). ^{13}C NMR (101 MHz, $CDCl_3$) δ 163.8 (*C*ONH), 155.9 (aryl-*C*), 139.1 (aryl-*C*), 132.1 (aryl-*C*), 131.7 (aryl-*C*), 128.8 (aryl-*C*), 128.5 (aryl-*C*), 126.4 (aryl-*C*), 126.4 (aryl-*C*), 122.9 (aryl-*C*), 112.8 (aryl-*C*), 56.0, 40.8, 35.4. These spectroscopic data correspond to reported data.[29]

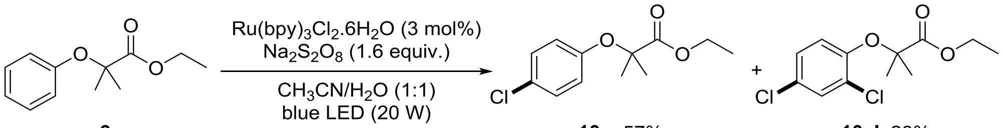

9 **10a**, 57% **10a'**, 28%

Clofibrate (10a). 10a and **10a'** was synthesized following the general procedure of chlorination. The residue was purified by chromatography on silica gel, eluting with hexane/ethyl acetate (10:1) to give the mixture of **10a** and **10a'**. These two compound were further separated by preparative HPLC (CHIRALPAK IC column, hexane/*i*PrOH = 99.2:0.8) to give the title compound as colorless oil (69.6 mg, 57%). ^{1}H NMR (400 MHz, $CDCl_3$) δ 7.18 (d, *J* = 8.9 Hz, 2H, aryl-*H*), 6.78 (d, *J* = 8.9 Hz, 2H, aryl-*H*), 4.22 (q, *J* = 7.1 Hz, 2H, OCH_2CH_3), 1.57 (s, 6H, $C(CH_3)_2$), 1.24 (t, *J* = 7.1 Hz, 3H, OCH_2CH_3). ^{13}C NMR (101 MHz, $CDCl_3$) δ 174.1 (*C*=O), 154.2

(aryl-*C*), 129.2 (aryl-*C*), 127.3 (aryl-*C*), 120.7 (aryl-*C*), 79.6, 61.6, 25.4, 14.2. These spectroscopic data correspond to reported data.[30]

Ethyl 2-(2,4-dichlorophenoxy)-2-methylpropanoate (10a’). 10a and **10a'** was synthesized following the general procedure of chlorination. The residue was purified by chromatography on silica gel, eluting with hexane/ethyl acetate (10:1) to give the mixture of **10a** and **10a'**. These two compound were further separated by preparative HPLC (CHIRALPAK IC column, hexane/*i*PrOH = 99.2:0.8) to give the title compound as colorless oil (39.0 mg, 28%). ^{1}H NMR (400 MHz, $CDCl_3$) 7.37 (d, J = 2.6 Hz, 1H, aryl-*H*), 7.09 (dd, J = 8.8, 2.6 Hz, 1H, aryl-*H*), 6.85 (d, J = 8.8 Hz, 1H, aryl-*H*), 4.23 (q, J = 7.1 Hz, 2H, OCH_2CH_3), 1.60 (s, 6H, $C(CH_3)_2$), 1.27 (t, J = 7.1 Hz, 3H, OCH_2CH_3). ^{13}C NMR (101 MHz, $CDCl_3$) δ 173.8 (*C*=O), 150.5 (aryl-*C*), 130.2 (aryl-*C*), 127.9 (aryl-*C*), 127.7 (aryl-*C*), 127.3 (aryl-*C*), 120.9 (aryl-*C*), 81.4, 61.8, 25.2, 14.2. These spectroscopic data correspond to reported data.[30]

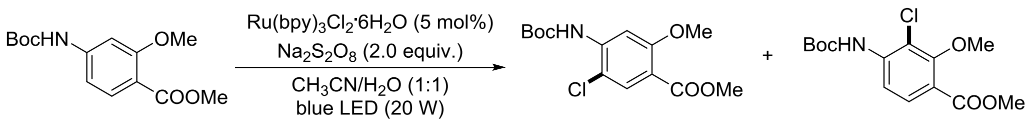

Methyl 4-((tert-butoxycarbonyl)amino)-5-chloro-2-methoxybenzoate (12a). 12a and **12a'** was synthesized following the general procedure of chlorination. The residue was purified by chromatography on silica gel, eluting with hexane/ethyl acetate (15:1~10:1) to give the title compound as colorless oil (91.3 mg, 58%). ^{1}H NMR (400 MHz, $CDCl_3$) δ 8.02 (s, 1H, aryl-*H*), 7.84 (s, 1H, aryl-*H*), 7.17 (s, 1H, N*H*), 3.93 (s, 3H), 3.85 (s, 3H), 1.53 (s, 9H, $C(CH_3)_3$). ^{13}C NMR (101 MHz, $CDCl_3$) δ 164.9, 159.4, 151.8 (aryl-*C*), 139.8 (aryl-*C*), 132.1 (aryl-*C*), 113.7 (aryl-*C*), 111.9 (aryl-*C*), 102.4 (aryl-*C*), 81.8 (*C*$(CH_3)_3$), 56.3 ($ArOCH_3$), 51.9 ($COOCH_3$), 28.2 ($C(CH_3)_3$). HRMS-ESI (m/z): Calcd for [($C_{14}H_{18}ClNO_5$+H)+], 316.0952; found: 316.0956.

Methyl 4-((tert-butoxycarbonyl)amino)-3-chloro-2-methoxybenzoate (12a’). 12a and **12a'** was synthesized following the general procedure of chlorination. The residue was purified by chromatography on silica gel, eluting with hexane/ethyl acetate

(15:1~10:1) to give the title compound as colorless oil (18.2 mg, 12%). ^{1}H NMR (400 MHz, $CDCl_3$) δ 8.06 (d, *J* = 8.9 Hz, 1H, aryl-*H*), 7.78 (d, *J* = 9.0 Hz, 1H, aryl-*H*), 7.23 (s, 1H, N*H*), 3.91 (s, 3H), 3.90 (s, 3H), 1.54 (s, 9H, C(C*H*$_3$)$_3$). ^{13}C NMR (101 MHz, $CDCl_3$) δ 165.2, 156.7, 151.8 (aryl-*C*), 140.2 (aryl-*C*), 130.5 (aryl-*C*), 119.2 (aryl-*C*), 117.0 (aryl-*C*), 113.7 (aryl-*C*), 81.8 (*C*(CH$_3$)$_3$), 61.9 (ArO*C*H$_3$), 52.1 (COO*C*H$_3$), 28.2 (C(*C*H$_3$)$_3$). HRMS-ESI (m/z): Calcd for [($C_{14}H_{18}ClNO_5$+H)+], 316.0952; found: 316.0957.

6. Mechanism study

Scheme S1. Two probably pathways of oxidative chlorination of aromatic compounds

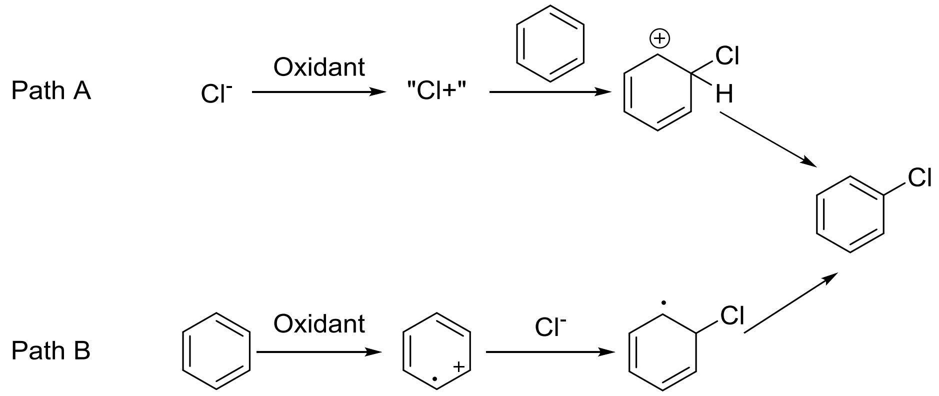

A:

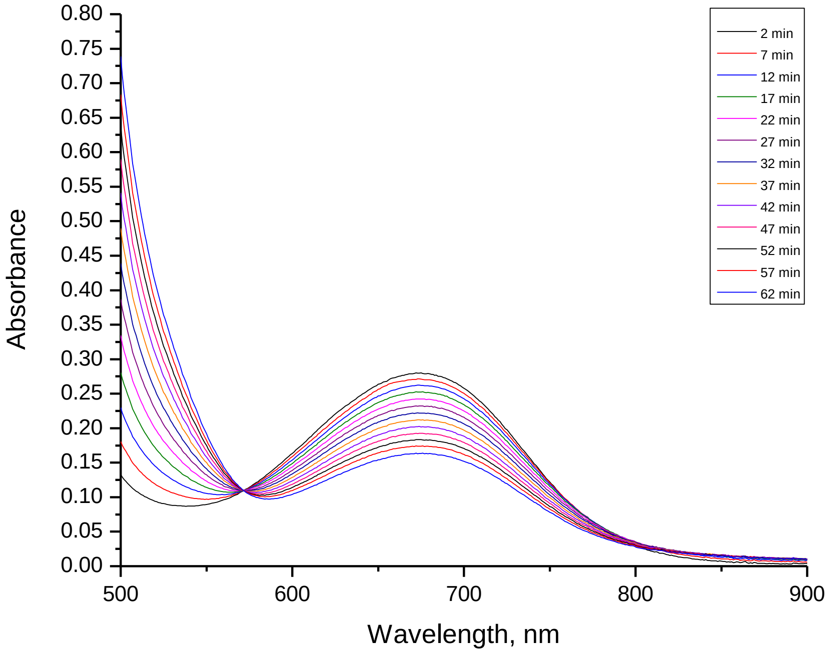

B:

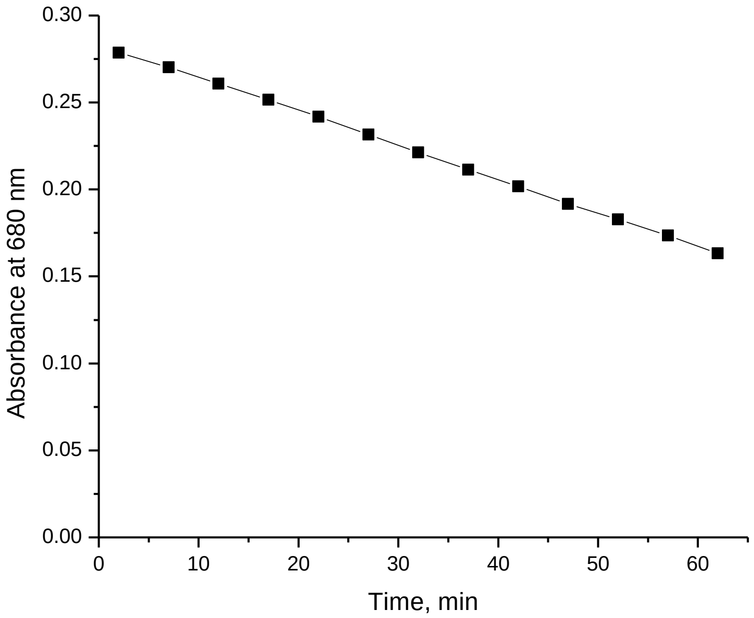

Figure S1. (A) Time-dependent UV-vis spectra of $Ru(bpy)_3^{3+}$ (1x 10^{-3} M) in CH_3CN/H_2O (1:1); (B) The variation of absorbance at 680 nm on UV-vis spectra.

A：

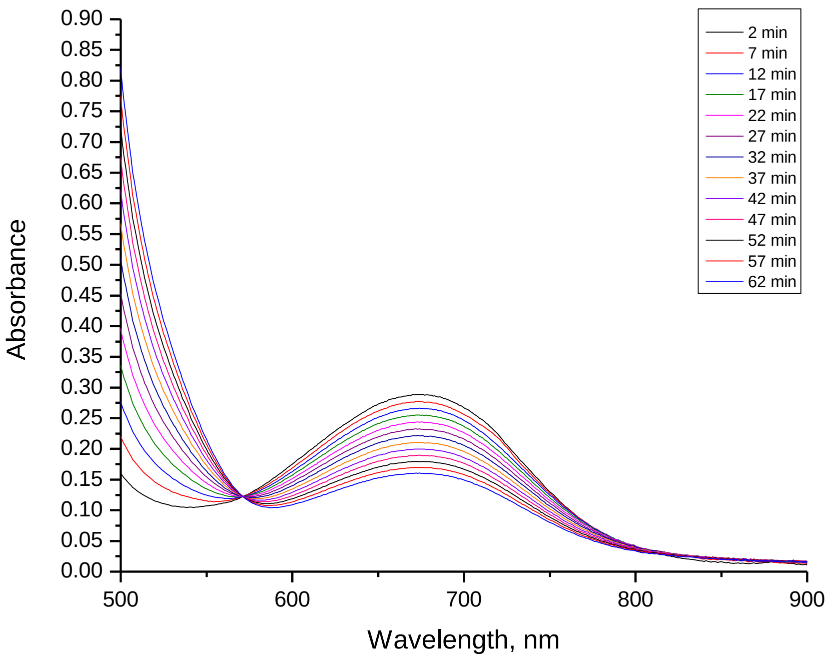

B：

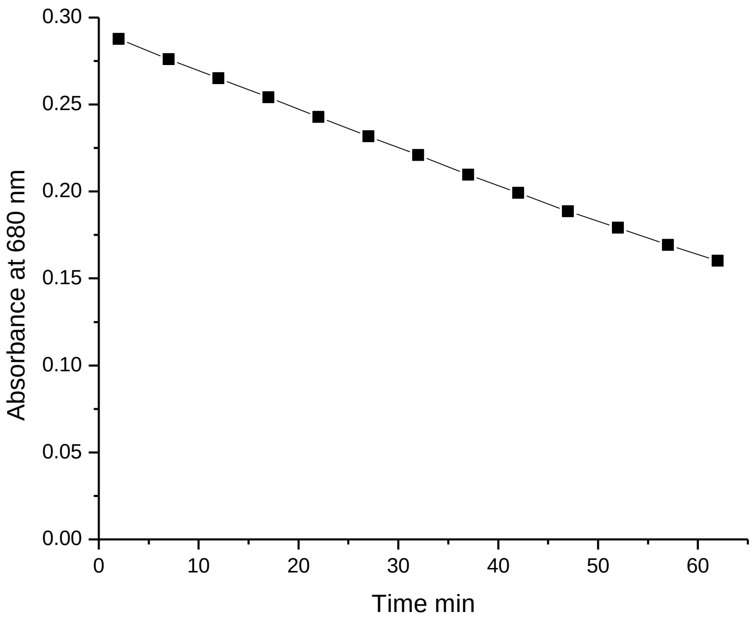

Figure S2. Time-dependent UV-vis spectra of $Ru(bpy)_3^{3+}$ (1x 10^{-3} M) with added toluene (2x 10^{-2} M) in CH_3CN/H_2O (1:1) ; (B) The variation of absorbance at 680 nm on UV-vis spectra.

A:

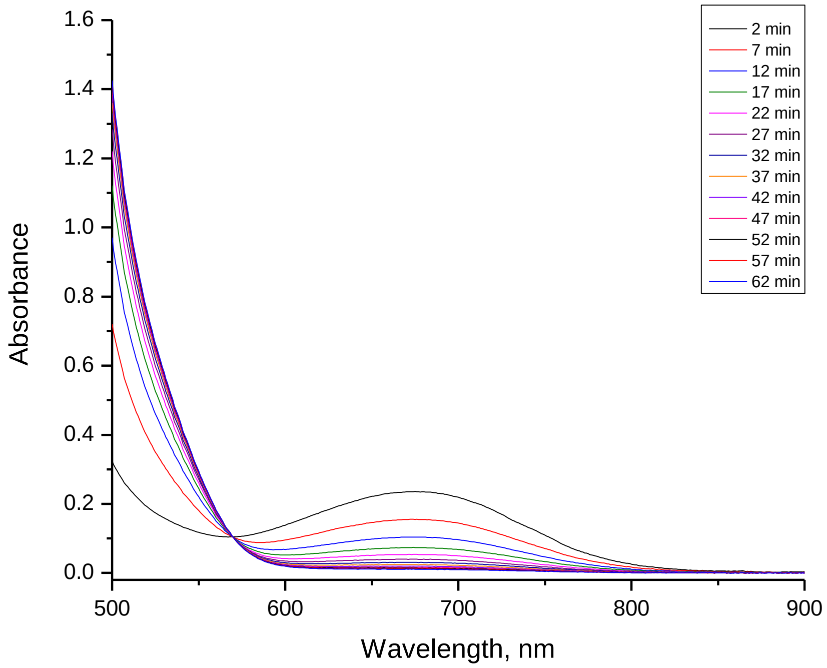

B:

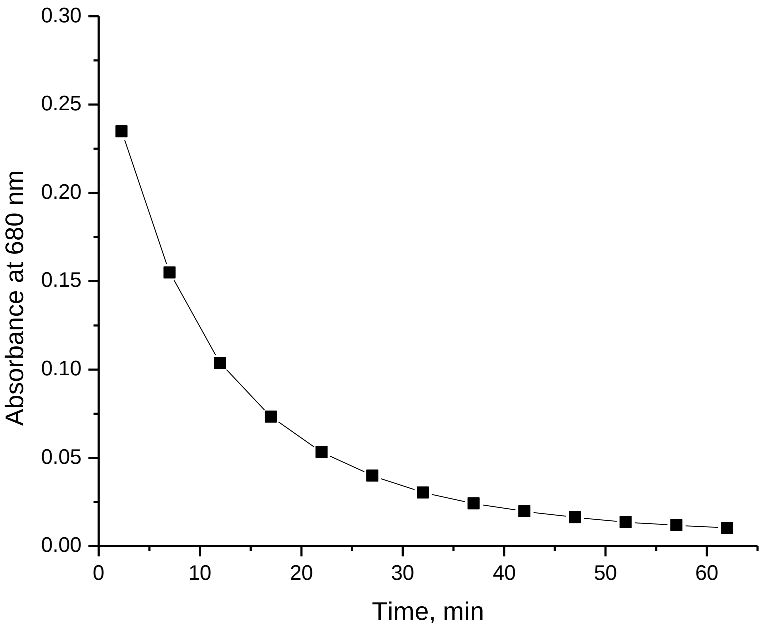

Figure S3. Time-dependent UV-vis spectra of $Ru(bpy)_3^{3+}$ (1x 10^{-3} M) with added NaCl (6 x 10^{-2} M) in CH_3CN/H_2O (1:1); (B) The variation of absorbance at 680 nm on UV-vis spectra.

7. References

[1] H. Lv, J. Song, Y. V. Geletii, J. W. Vickers, J. M. Sumliner, D. G. Musaev, P. Kögerler, P. F. Zhuk, J. Bacsa, G. Zhu, C. L. Hill, *J. Am. Chem. Soc.* 2014, **136**, 9268.
[2] Y. Gao, Y. Huang, W. Wu, K. Huang, H. Jiang, *Chem. Commun.* 2014, **50**, 8370.
[3] Z. Zhuang, Z.-P. Hu, W.-W. Liao, *Org. Lett.* 2014, **16**, 3380.
[4] C. K. Lee, J. S. Yu, H.-J. Lee, *J. Heterocycl. Chem.* 2002, **39**, 1207.
[5] C. Yamamoto, K. Takamatsu, K. Hirano, M. Miura, *J. Org. Chem.* 2016, **81**, 7675.
[6] L. Kevin, W. M. James, A. N. Stewart, L. W. Paul, 2004, WO2004092117A1.
[7] X. Xiong, Y.-Y. Yeung, *Angew. Chem. Int. Ed.* 2016, **55**, 16101.
[8] C. Leveque, L. Chenneberg, V. Corce, J.-P. Goddard, C. Ollivier, L. Fensterbank, *Org. Chem. Front.* 2016, **3**, 462.
[9] K. Gao, N. Yoshikai, *J. Am. Chem. Soc.* 2013, **135**, 9279.
[10] D. G. C. Philip, R. D. Brian, O. David, A. A. Paul, N. Manuel, S. Frank, A. Stephanie, B. Nick, R. B. Thomas, B. Angelica, B. Judd, A. C. Robert, R. E. Bruce, K. Harpreet, M. David, P. Vinh, R. Andrew, W. Phil, W. Jeffrey, 2011, WO2011126567A1.
[11] N. Miyoshi, T. Matsuo, M. Wada, *Eur. J. Org. Chem.* 2005, 4253.
[12] P. I. Fedorov, T. P. Fedorova, V. P. Sheverdov, G. P. Pavlov, A. V. Eremkin, *Russ. J. Org. Chem.* 2016, **52**, 806.
[13] P. S. Mahajan, V. T. Humne, S. D. Tanpure, S. B. Mhaske, *Org. Lett.* 2016, **18**, 3450.
[14] Y. Peng, H. Liu, M. Tang, L. Cai, V. Pike, *Chin. J. Chem* . 2009, **27**, 1339.
[15] J. B. McManus, D. A. Nicewicz, *J. Am. Chem. Soc.* 2017, **139**, 2880.
[16] X. Zhang, C. Lou, N. Li, X. Xu, R. Qiu, S. Yin, *J. Organomet. Chem.* 2014, **749**, 241.
[17] R. M. Riggs, D. E. Nichols, M. M. Foreman, L. L. Truex, *J. Med. Chem.* 1987, **30**, 1887.
[18] T. D. Huber, F. Wang, S. Singh, B. R. Johnson, J. Zhang, M. Sunkara, S. G. Van Lanen, A. J. Morris, G. N. Phillips, J. S. Thorson, *ACS Chem. Biol.* 2016, **11**, 2484.
[19] R. Prebil, K. K. Laali, S. Stavber, *Org. Lett.* 2013, **15**, 2108.
[20] S. Kathiravan, I. A. Nicholls, *Chem. Eur. J.* 2017, **23**, 7153.
[21] M. Gantenbein, M. Hellstern, L. Le Pleux, M. Neuburger, M. Mayor, *Chem. Mater.* 2015, **27**, 1772.
[22] T. Tamura, K. Moriyama, H. Togo, *Eur. J. Org. Chem.* 2015, 2023.
[23] A. Ueno, M. Takimoto, W. W. N. O, M. Nishiura, T. Ikariya, Z. Hou, *Chemistry – An Asian Journal* 2015, **10**, 1010.
[24] M. A. B. Mostafa, E. D. D. Calder, D. T. Racys, A. Sutherland, *Chem. Eur. J.* 2017, **23**, 1044.
[25] G. A. Molander, L. N. Cavalcanti, *J. Org. Chem.* 2011, **76**, 7195.
[26] S. M. Wilkinson, H. Gunosewoyo, M. L. Barron, A. Boucher, M. McDonnell, P. Turner, D. E. Morrison, M. R. Bennett, I. S. McGregor, L. M. Rendina, M. Kassiou, *ACS Chem. Neurosci.* 2014, **5**, 335.
[27] A. M. Costero, M. Pitarch, *J. Org. Chem.* 1994, **59**, 2939.
[28] F. Mo, J. M. Yan, D. Qiu, F. Li, Y. Zhang, J. Wang, *Angew. Chem. Int. Ed.* 2010, **49**, 2028.
[29] D. Obermayer, D. Znidar, G. Glotz, A. Stadler, D. Dallinger, C. O. Kappe, *J. Org. Chem.* 2016, **81**, 11788.
[30] D. Ok Jang, D. Hyan Cho, J.-G. Kim, *Synth. Commun.* 2003, **33**, 2885.
[31] T. M. Donald, A. Daniel, P. P.Norton, D. W. John, 2010, WO2010118046A1.

8. NMR Spectra

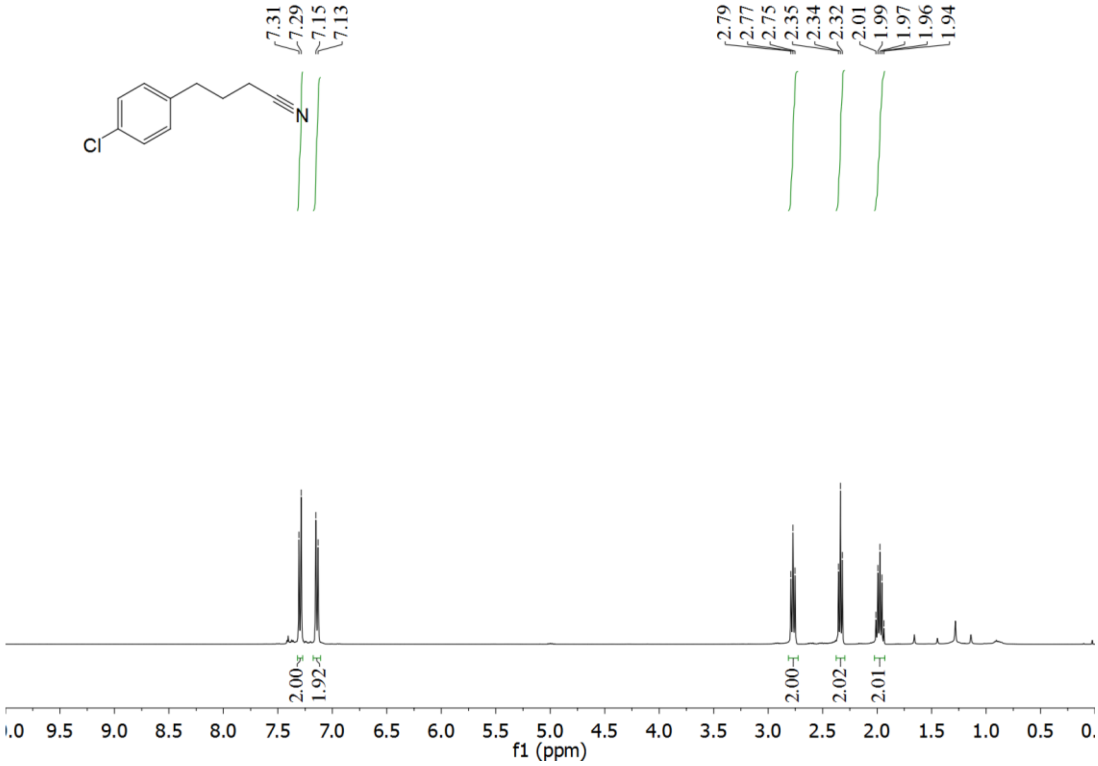

^{1}H NMR (400 MHz, $CDCl_3$) of **2d**

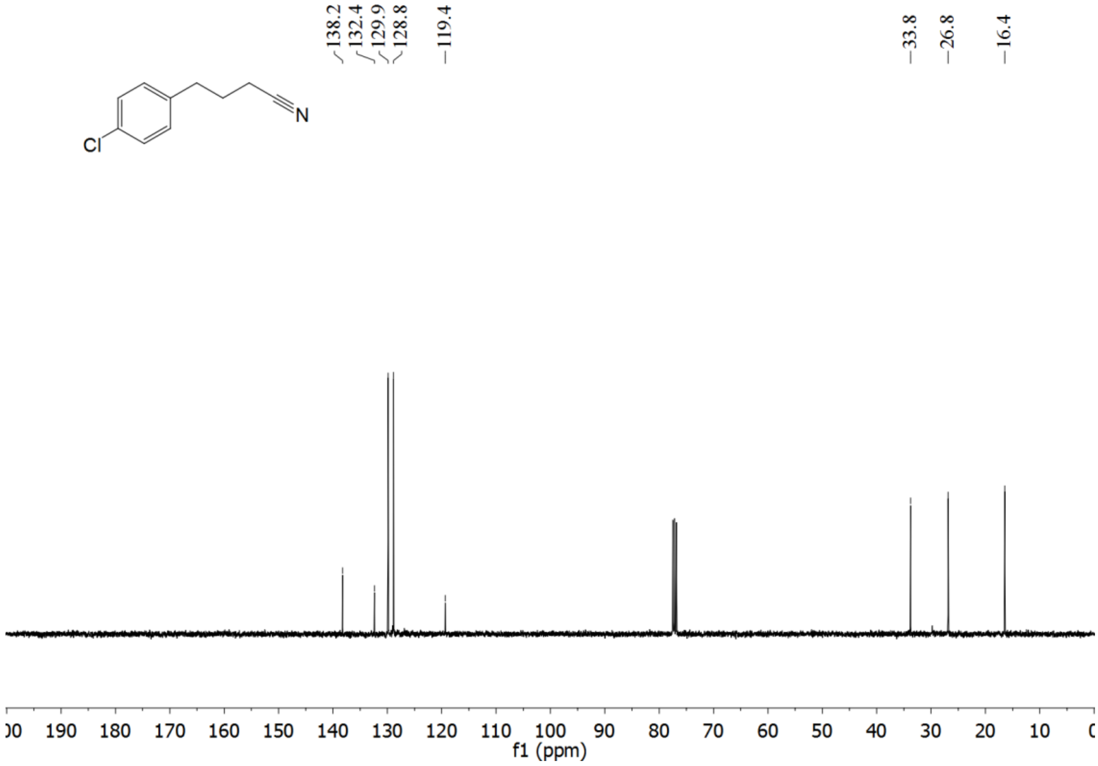

^{13}C NMR (101 MHz, $CDCl_3$) of **2d**

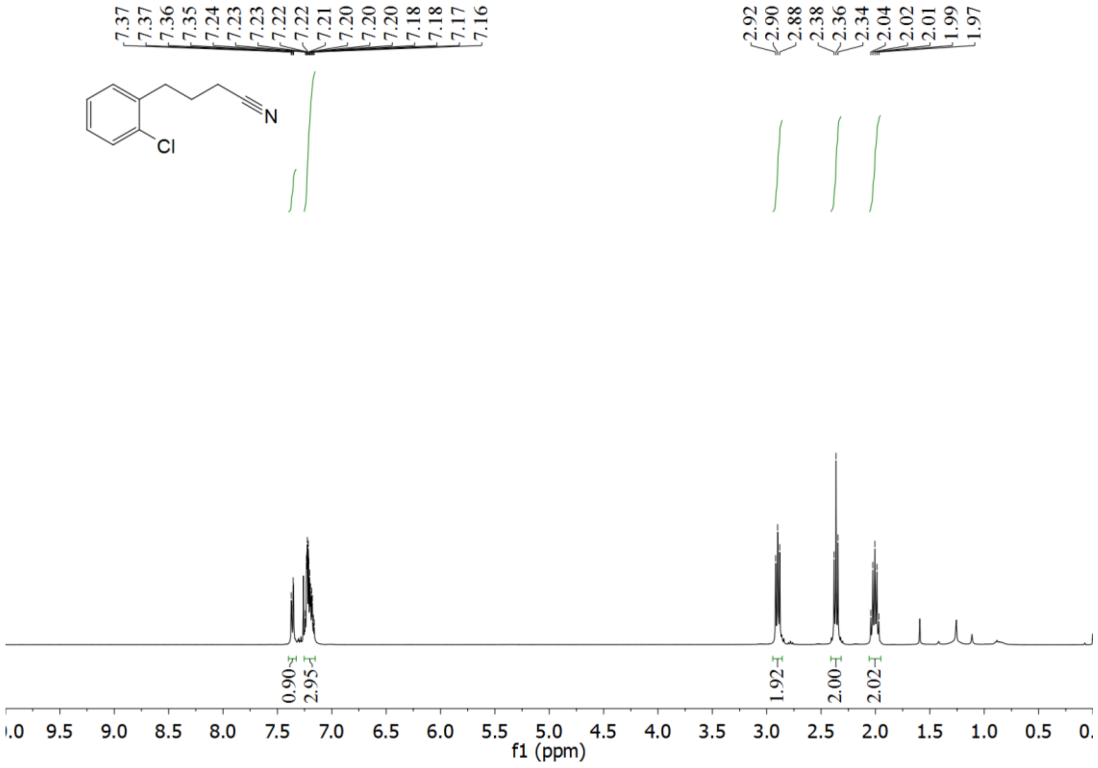

^{1}H NMR (400 MHz, $CDCl_3$) of **2d'**

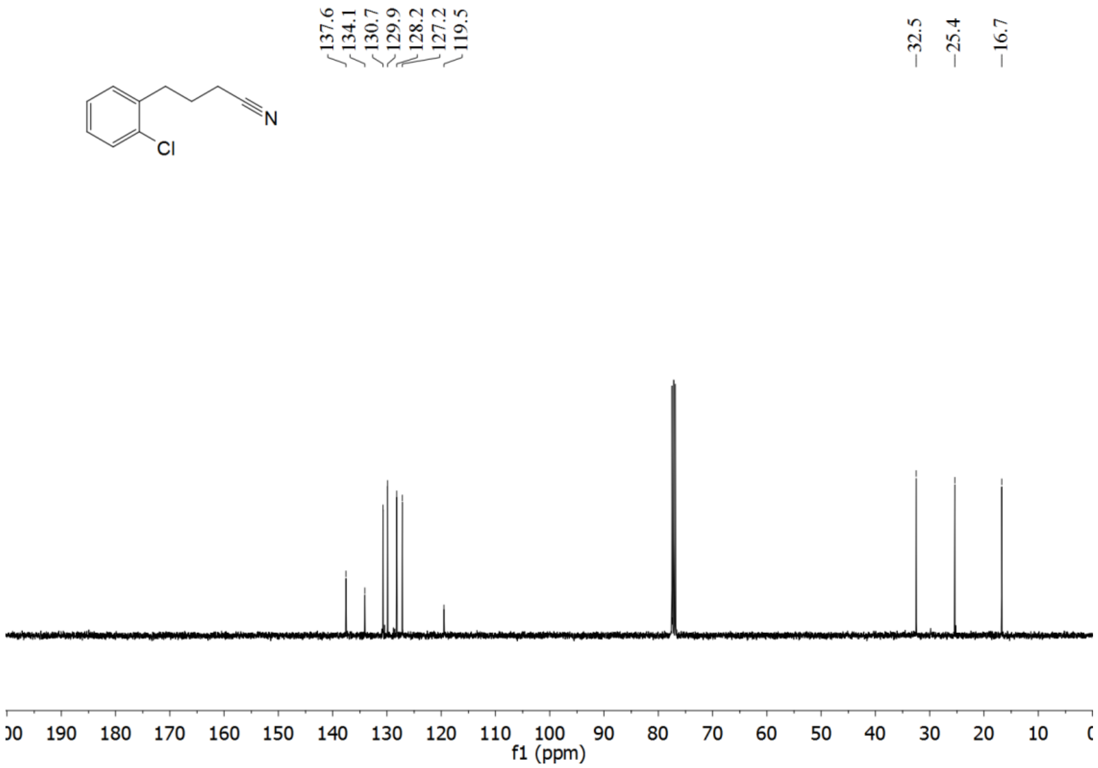

^{13}C NMR (101 MHz, $CDCl_3$) of **2d'**

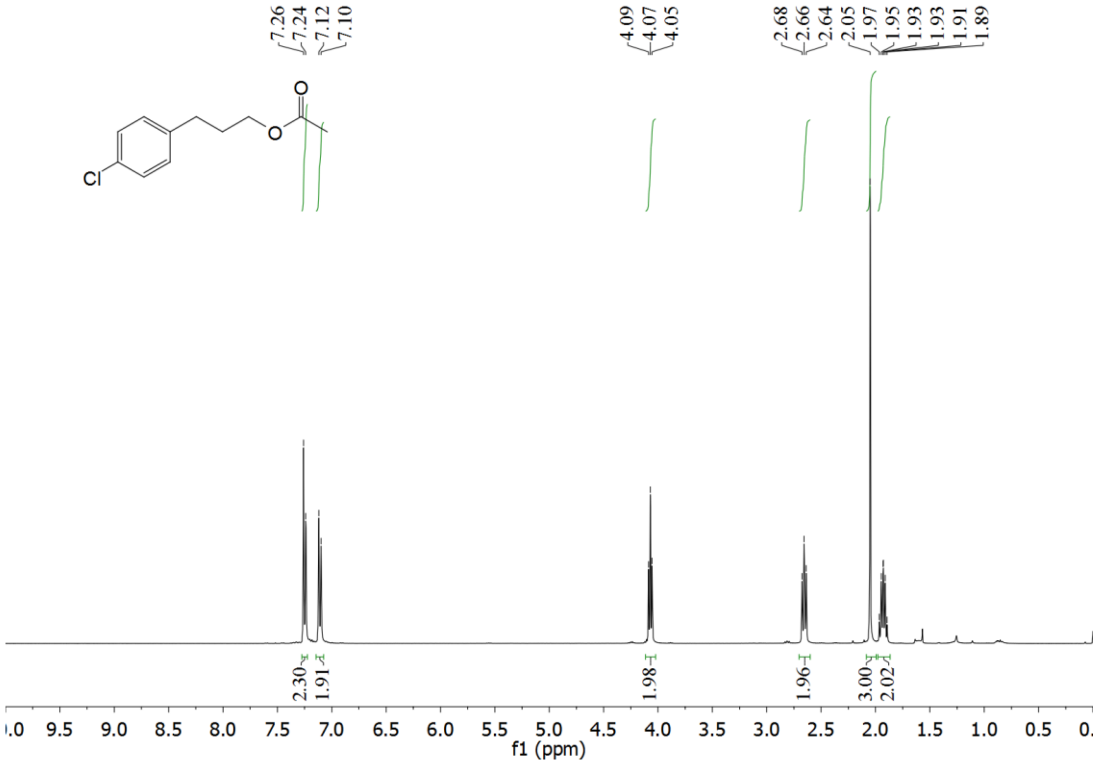

^{1}H NMR (400 MHz, $CDCl_3$) of **2e**

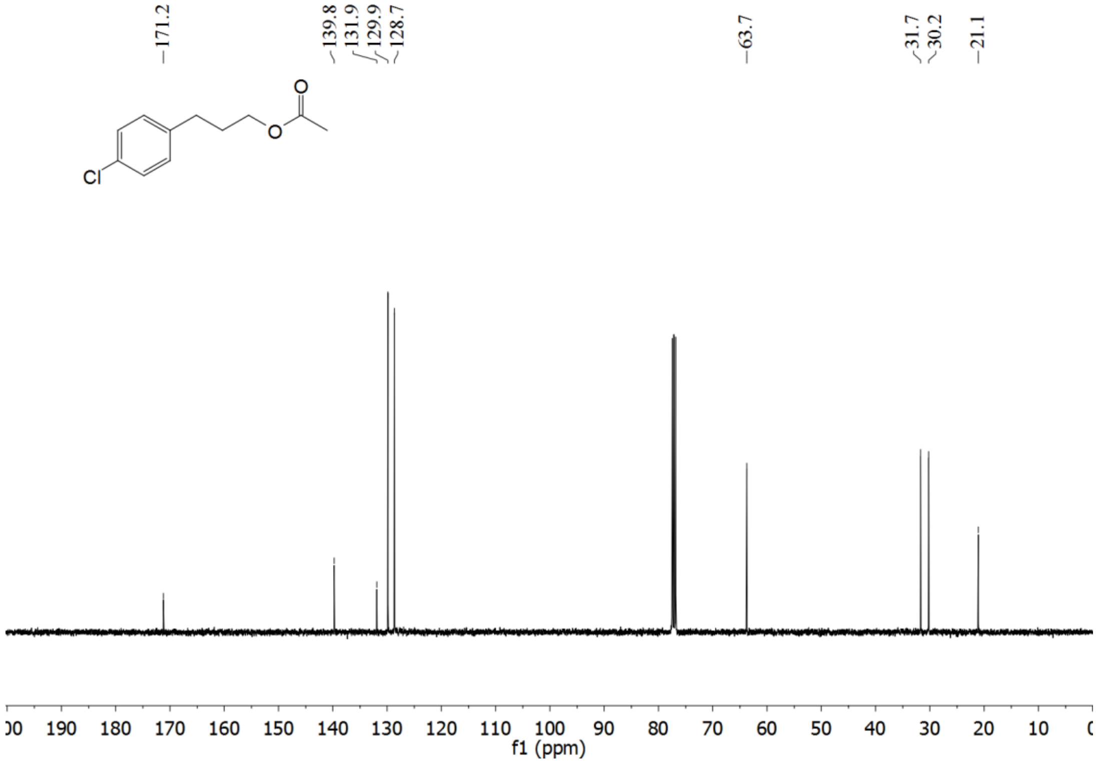

^{13}C NMR (101 MHz, $CDCl_3$) of **2e**

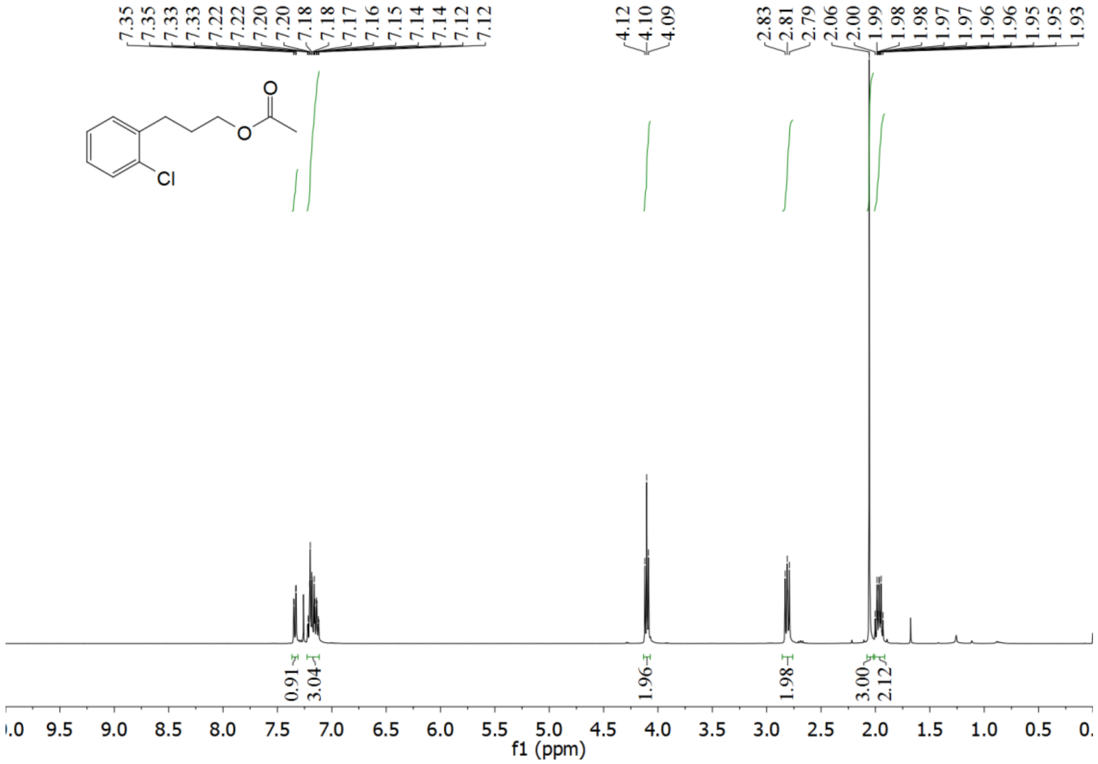

^1H NMR (400 MHz, $CDCl_3$) of **2e'**

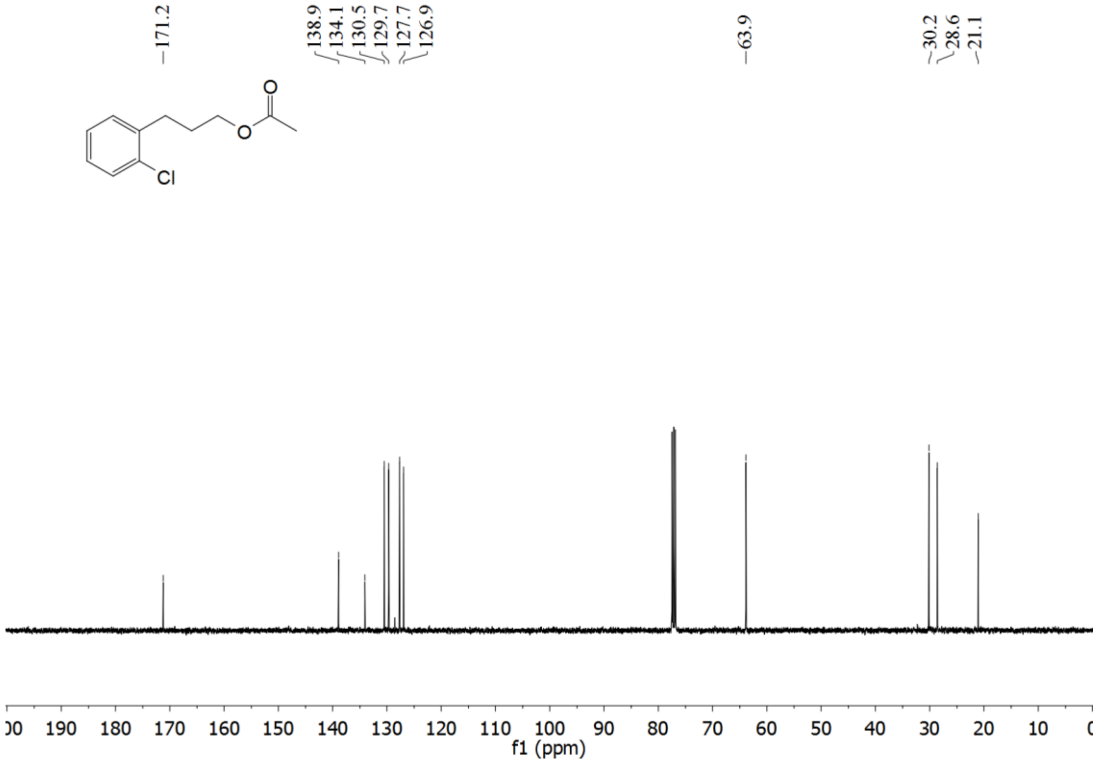

^{13}C NMR (101 MHz, $CDCl_3$) of **2e'**

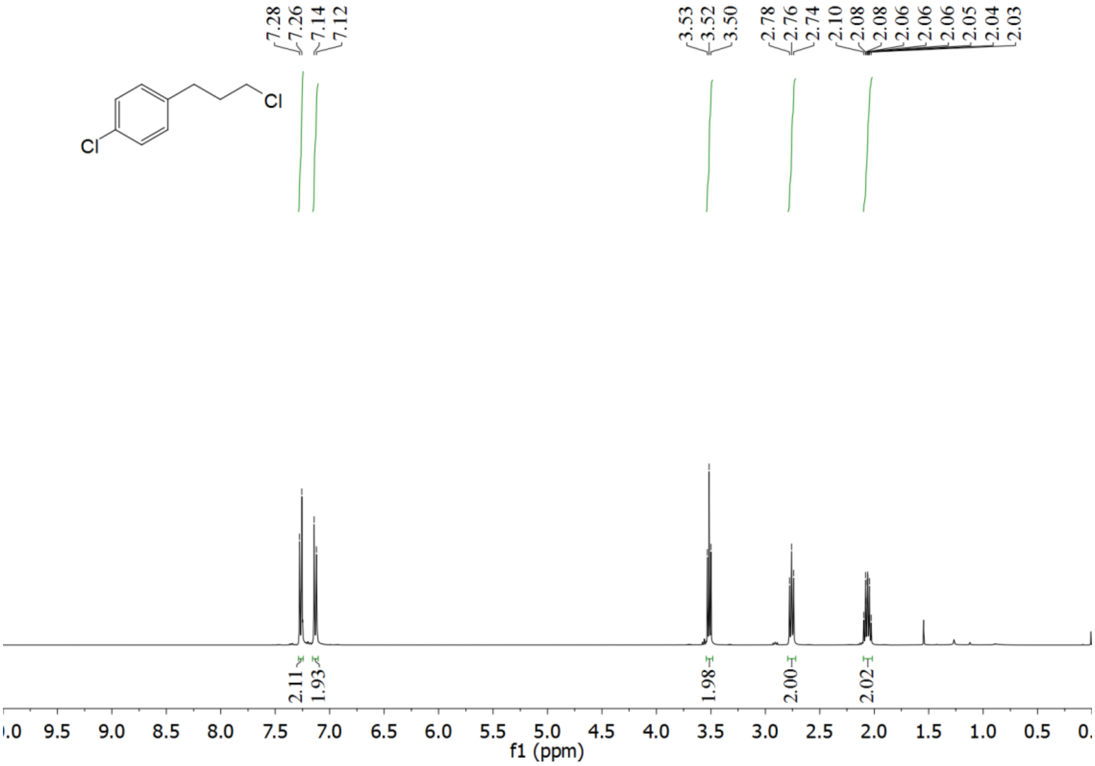

^{1}H NMR (400 MHz, $CDCl_3$) of **2f**

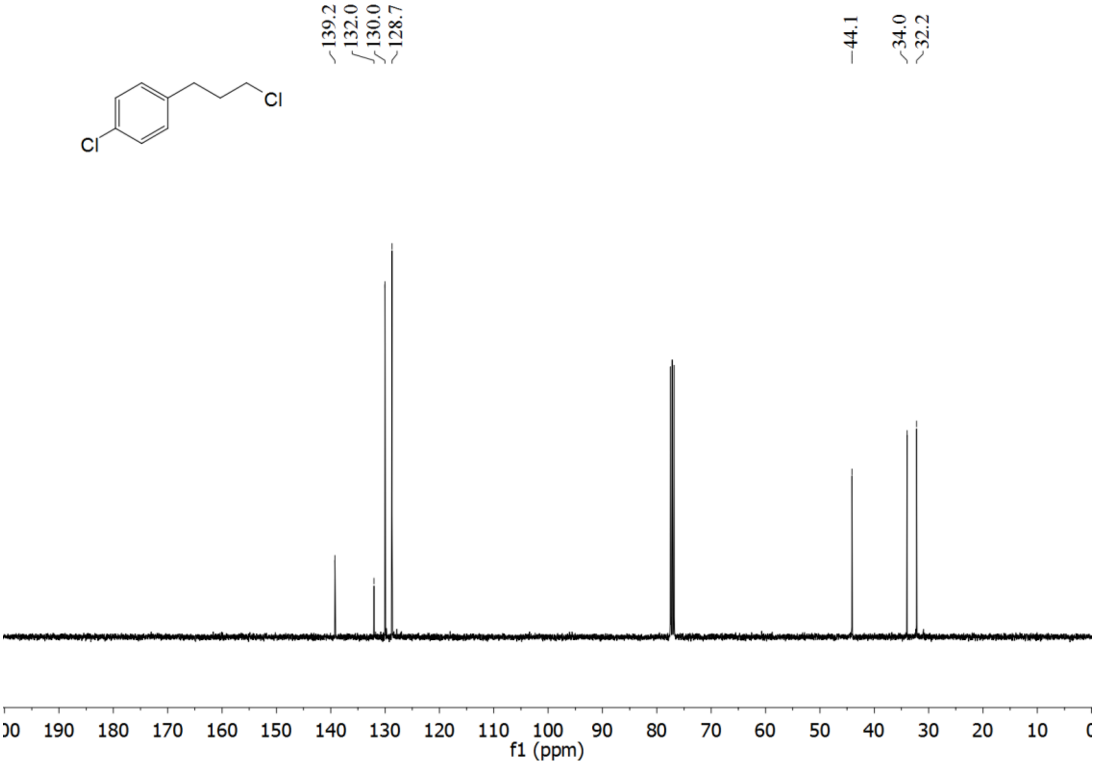

^{13}C NMR (101 MHz, $CDCl_3$) of **2f**

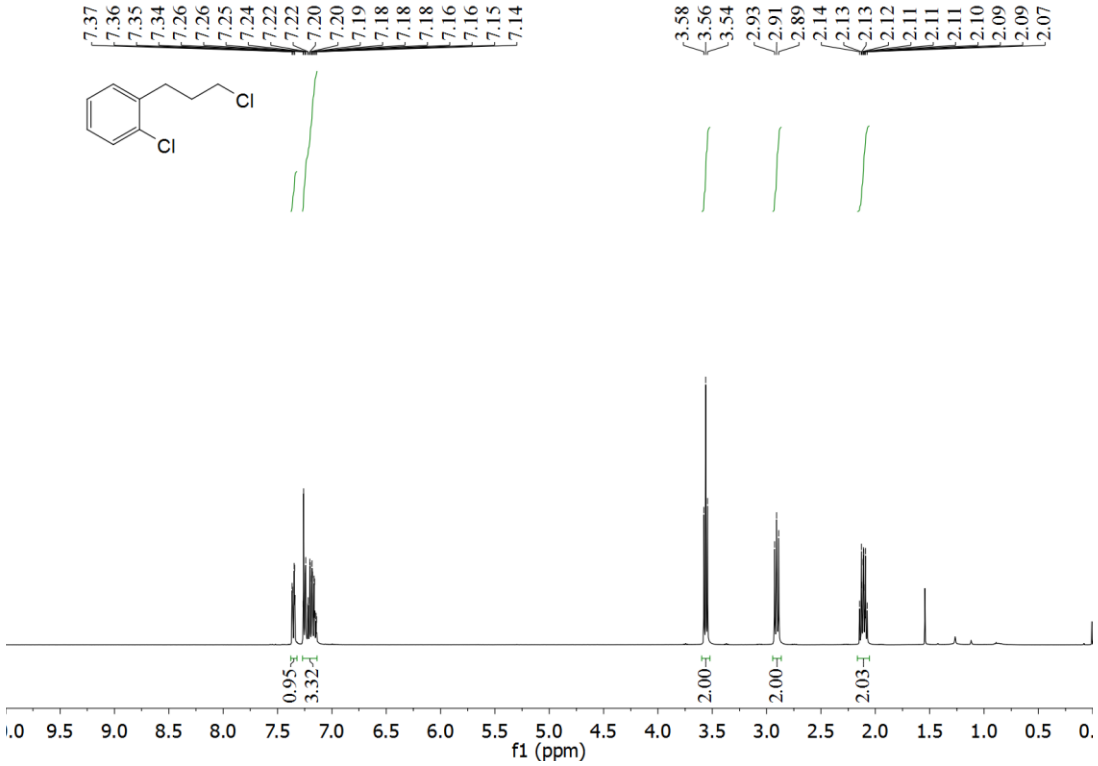

^{1}H NMR (400 MHz, $CDCl_3$) of **2f’**

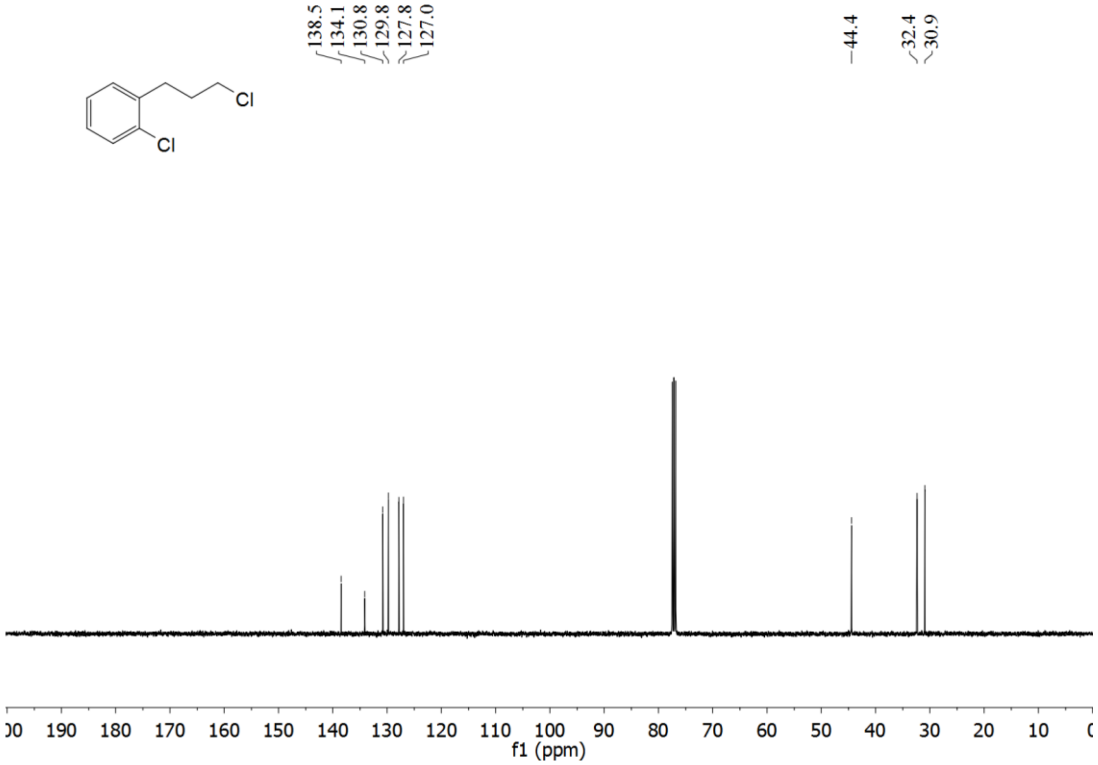

^{13}C NMR (101 MHz, $CDCl_3$) of **2f’**

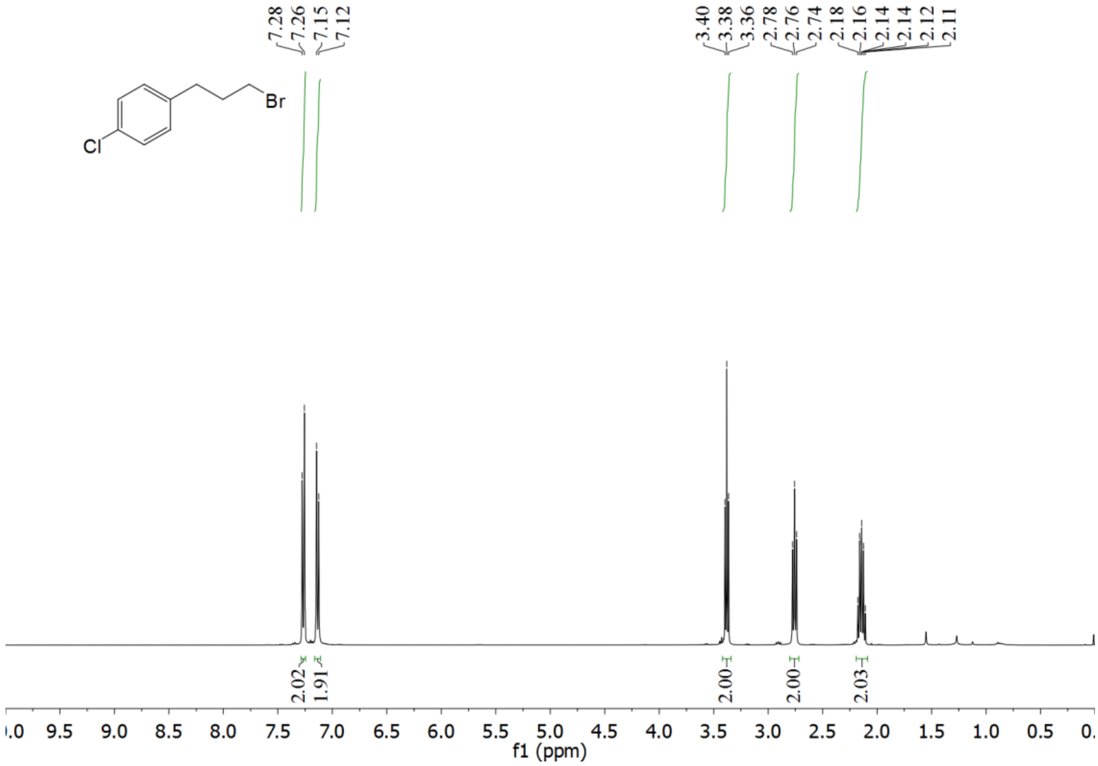

^{1}H NMR (400 MHz, $CDCl_3$) of **2g**

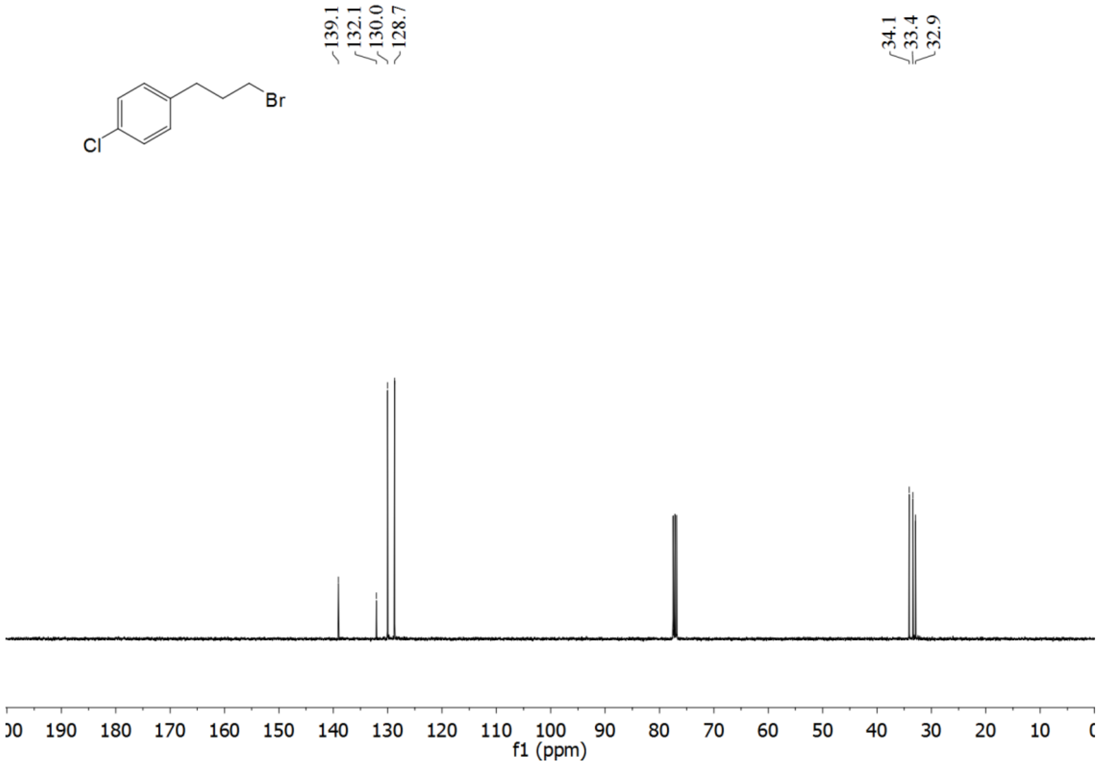

^{13}C NMR (101 MHz, $CDCl_3$) of **2g**

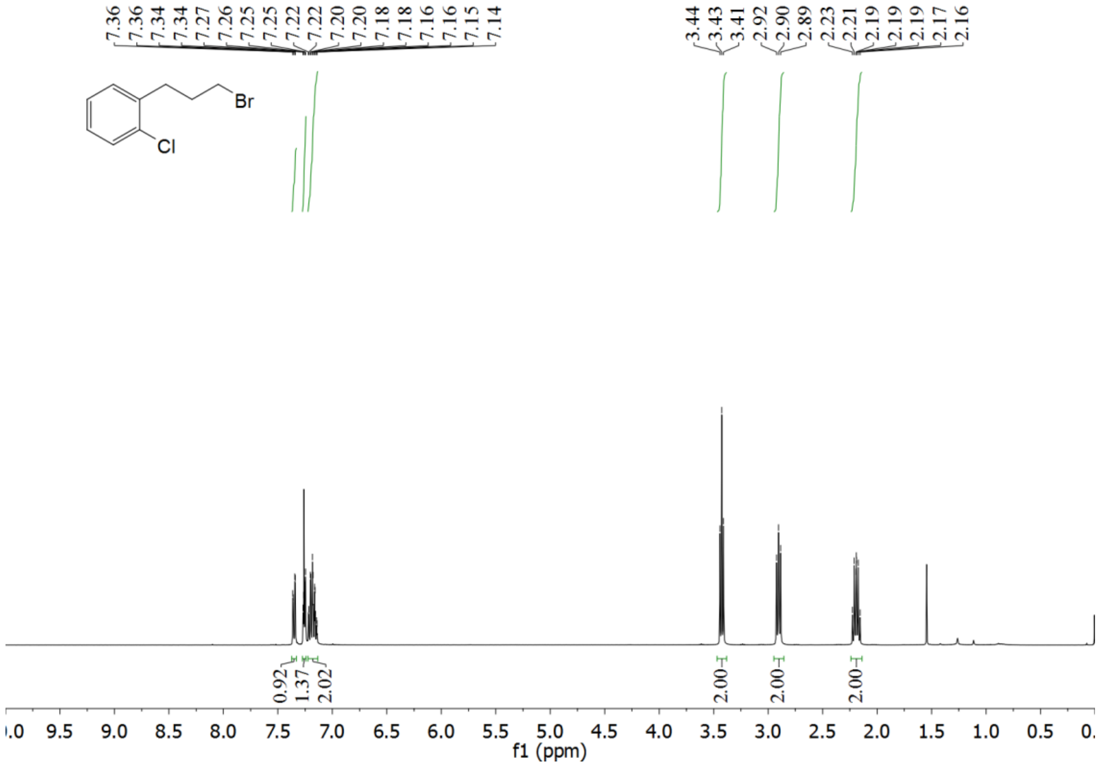

^{1}H NMR (400 MHz, $CDCl_3$) of **2g’**

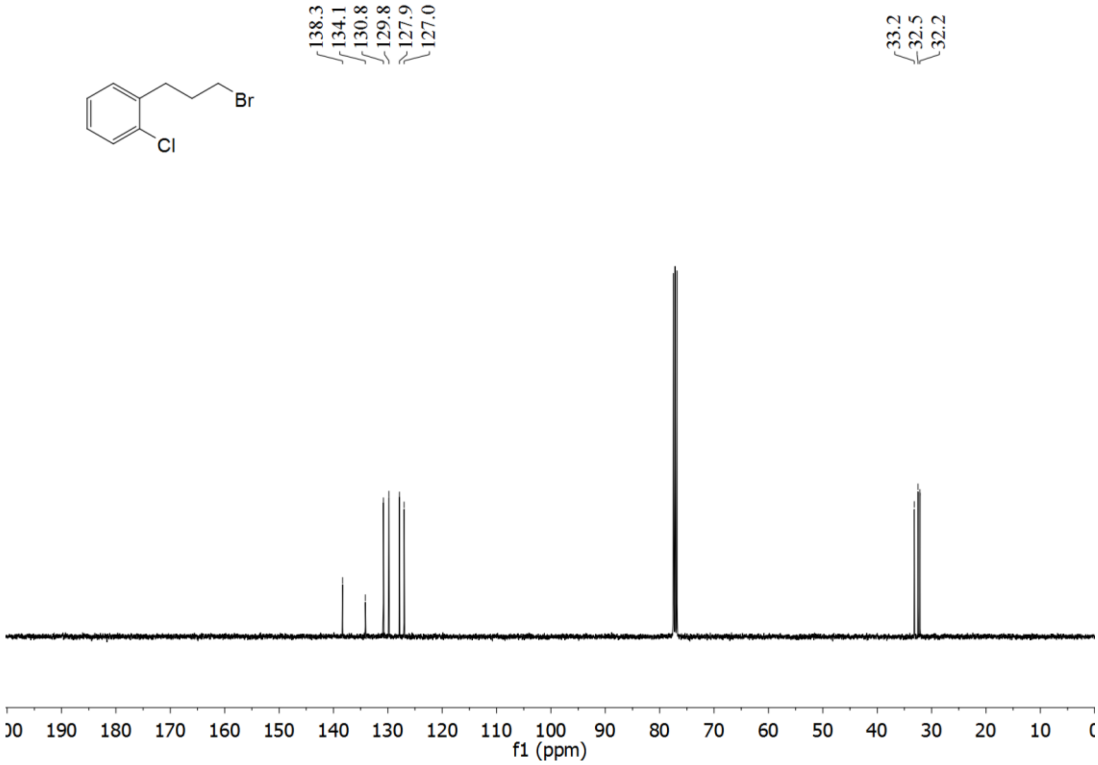

^{13}C NMR (101 MHz, $CDCl_3$) of **2g’**

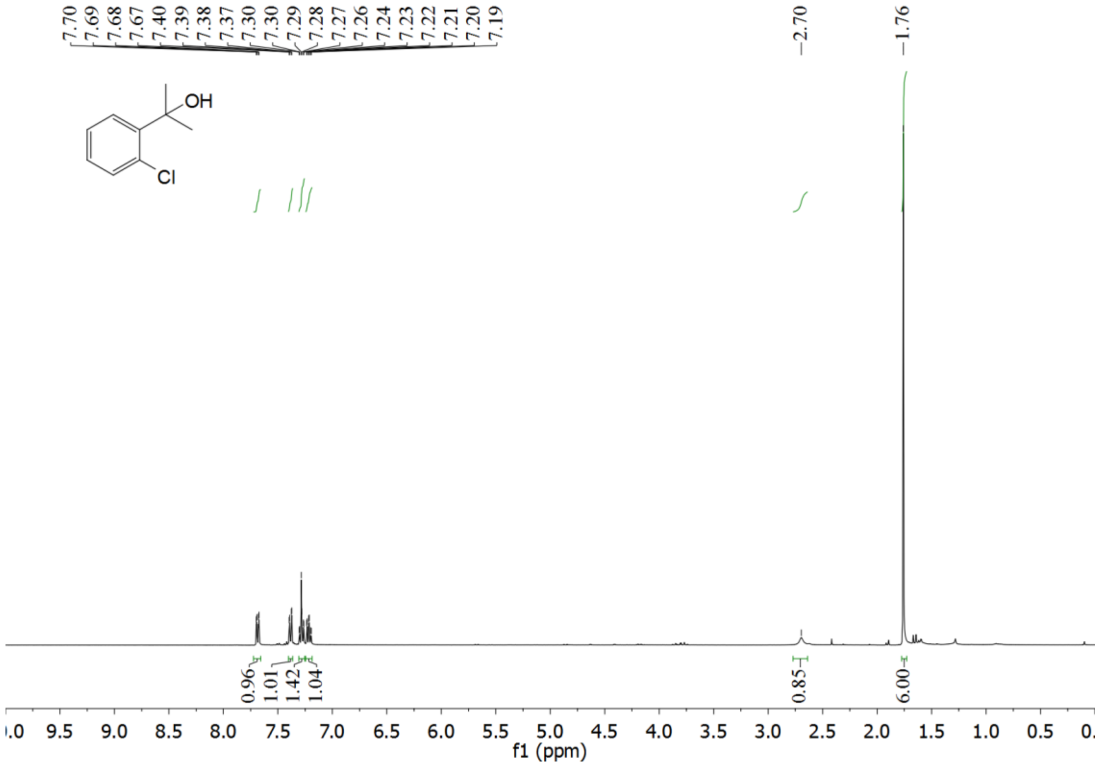

^{1}H NMR (400 MHz, $CDCl_3$) of **2h**

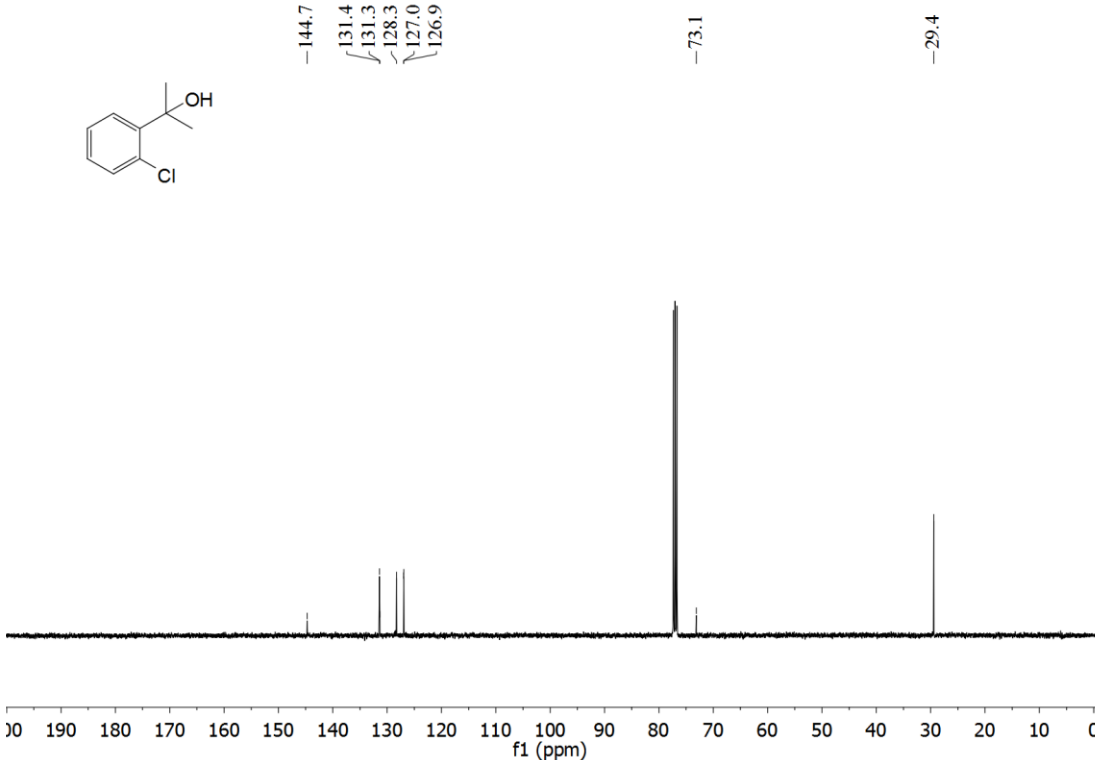

^{13}C NMR (101 MHz, $CDCl_3$) of **2h**

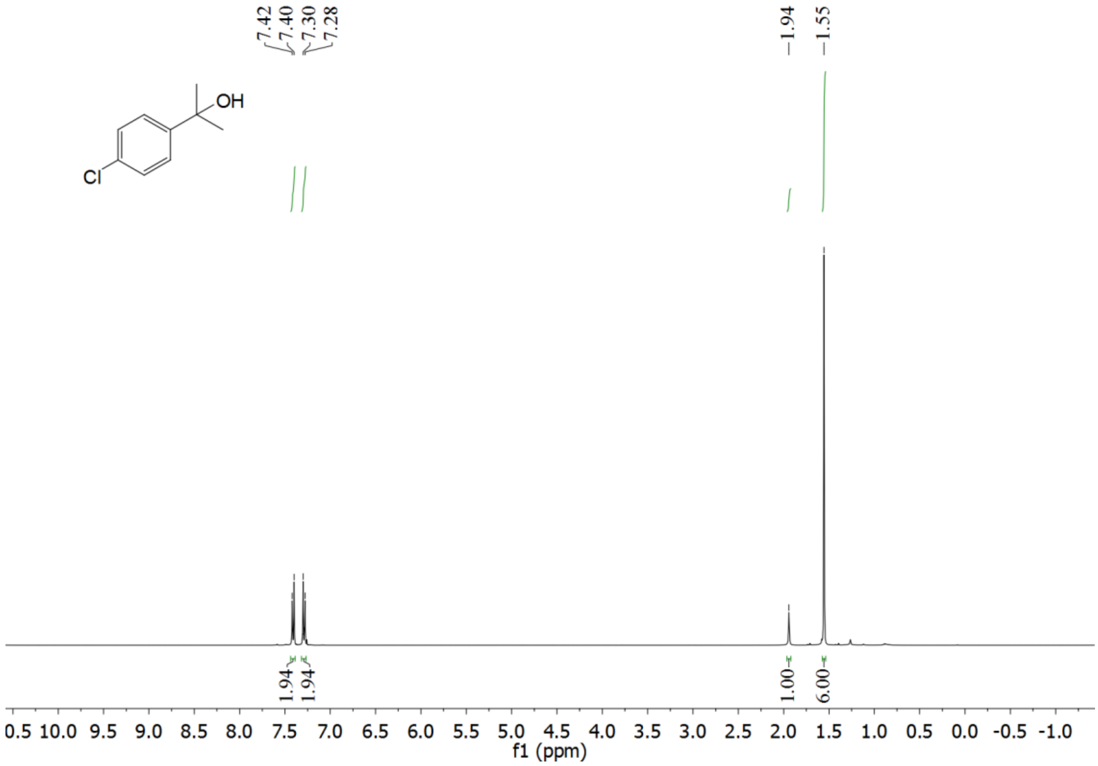

^{1}H NMR (400 MHz, $CDCl_3$) of **2h'**

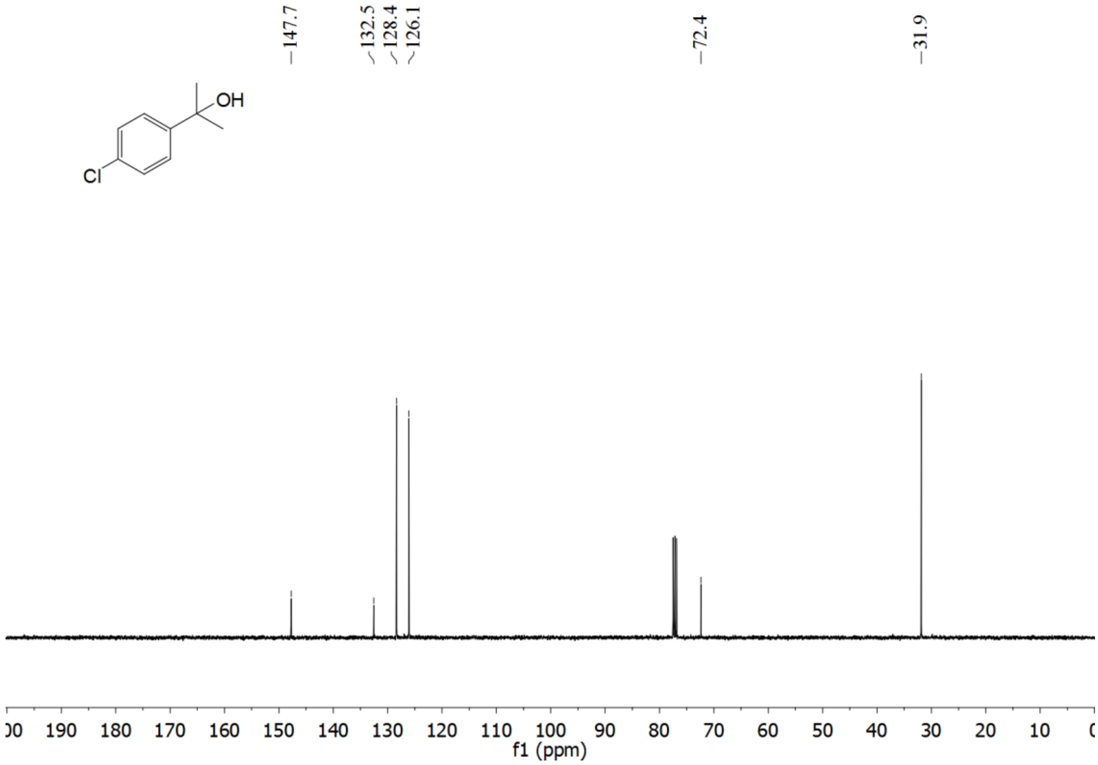

^{13}C NMR (101 MHz, $CDCl_3$) of **2h'**

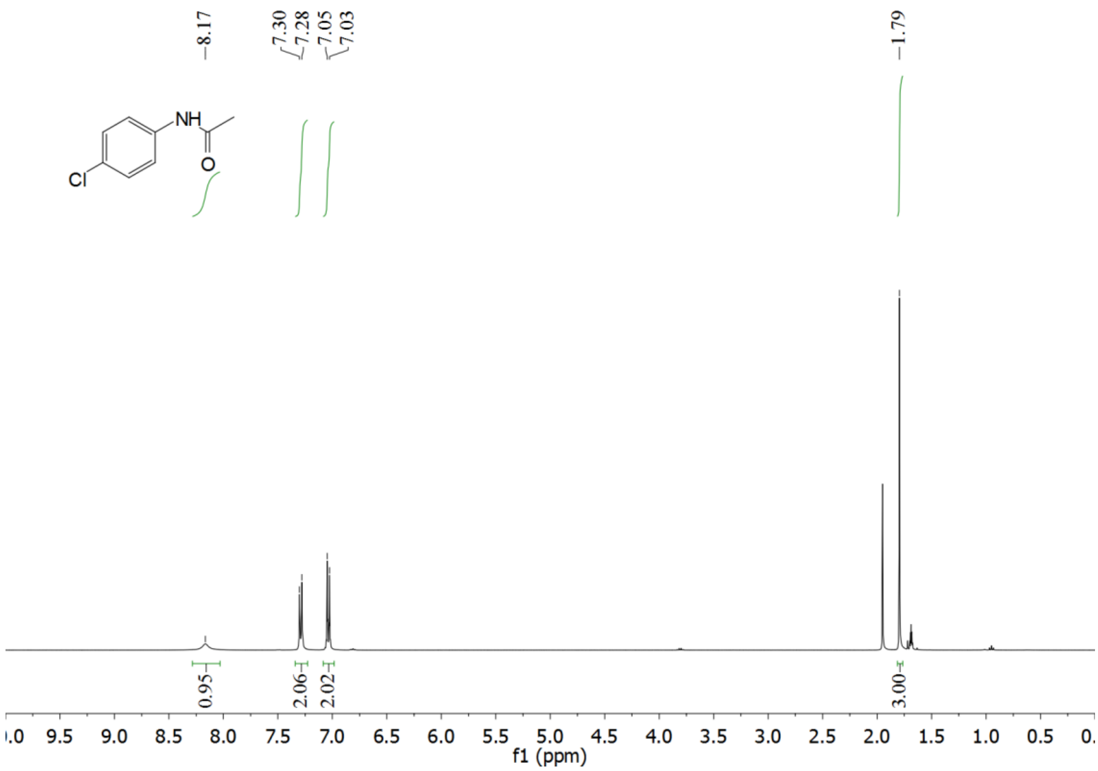

^{1}H NMR (400 MHz, $CDCl_3$) of **2i**

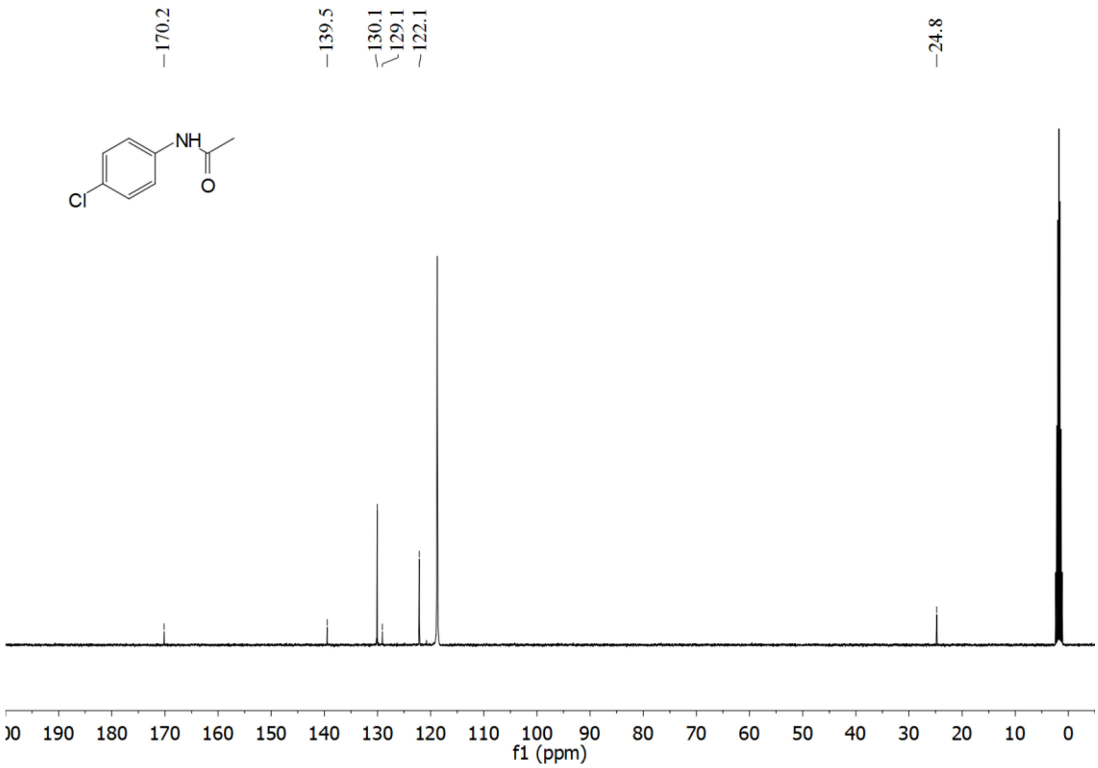

^{13}C NMR (101 MHz, $CDCl_3$) of **2i**

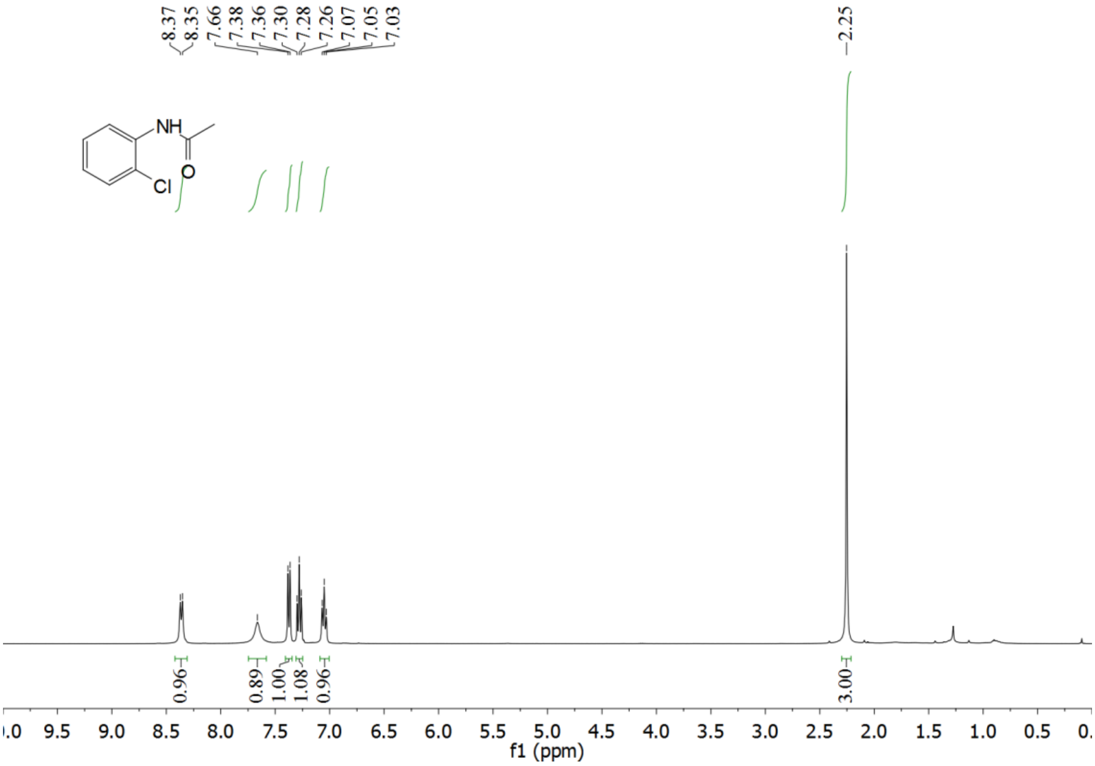

^{1}H NMR (400 MHz, $CDCl_3$) of **2i'**

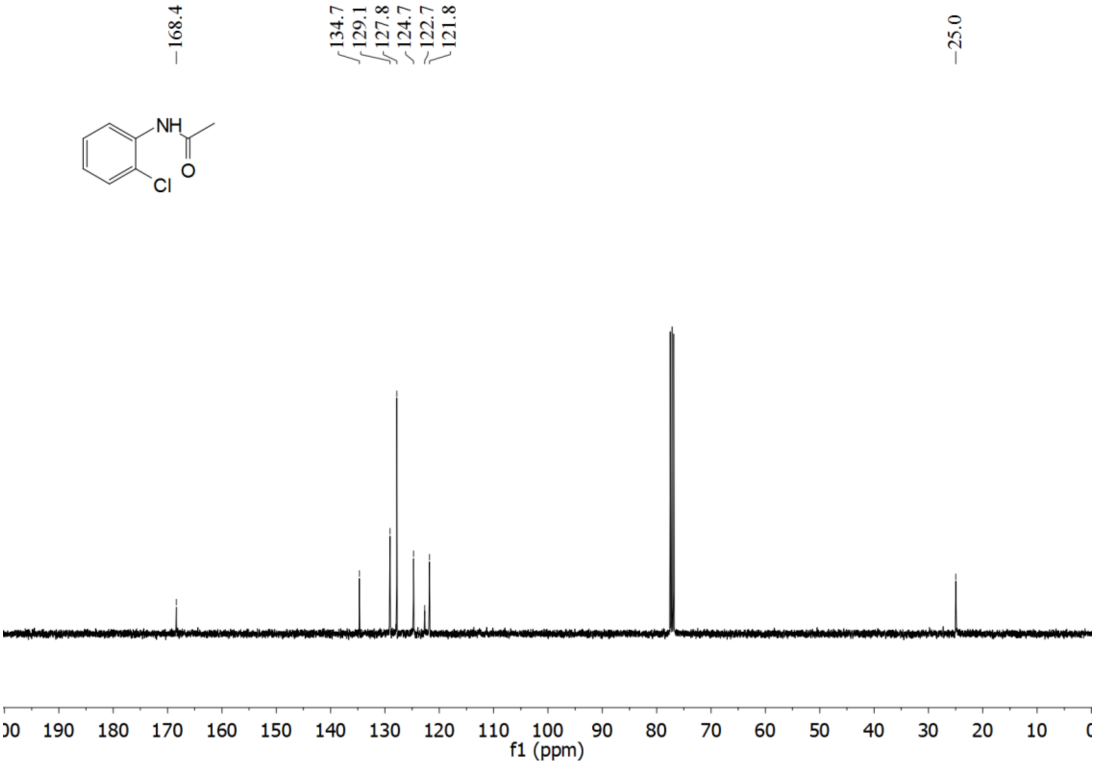

^{13}C NMR (101 MHz, $CDCl_3$) of **2i'**

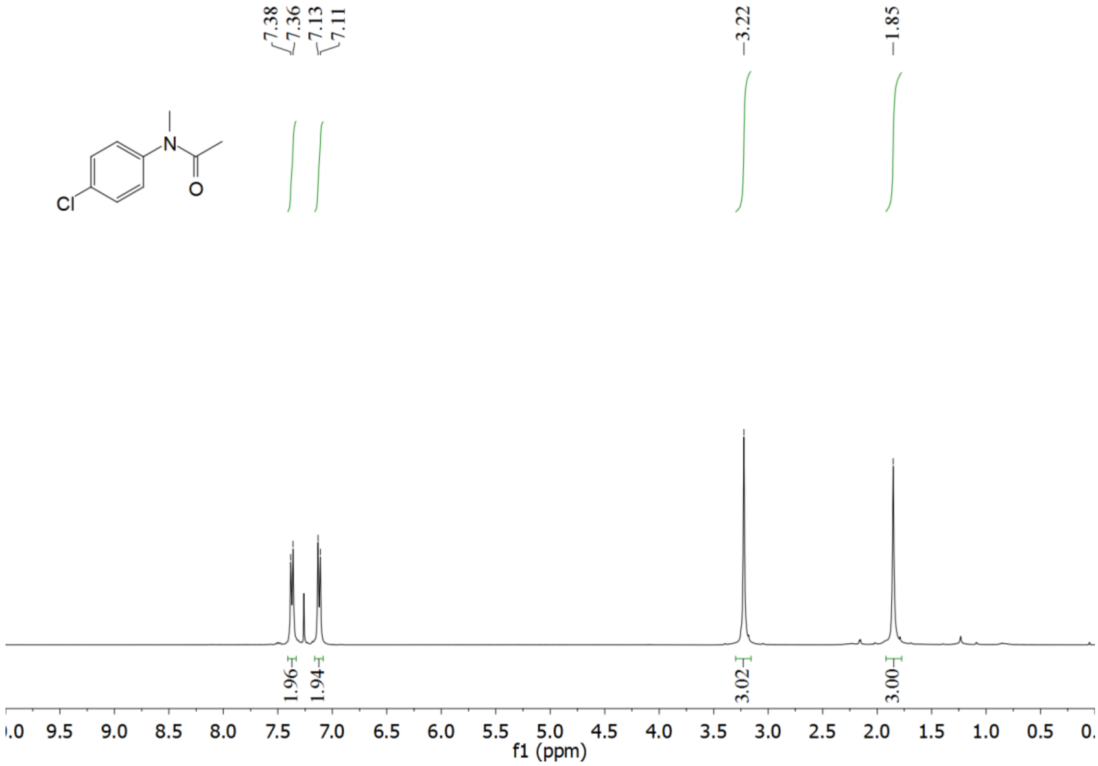

^{1}H NMR (400 MHz, $CDCl_3$) of **2j**

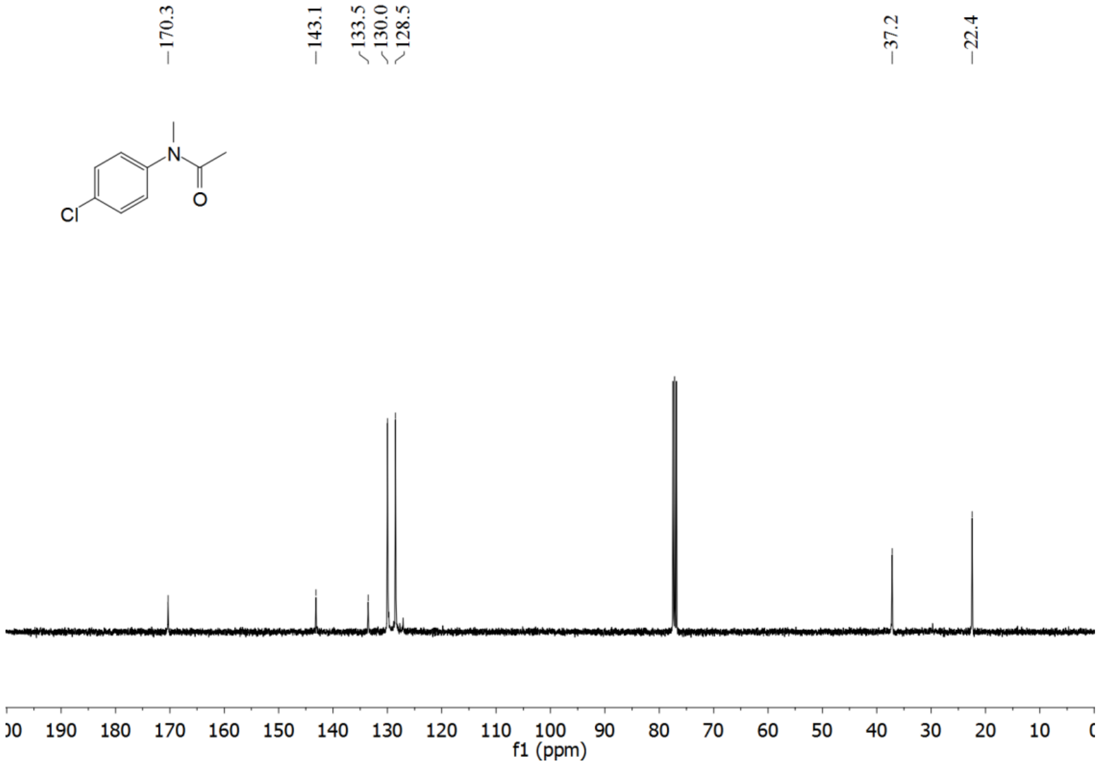

^{13}C NMR (101 MHz, $CDCl_3$) of **2j**

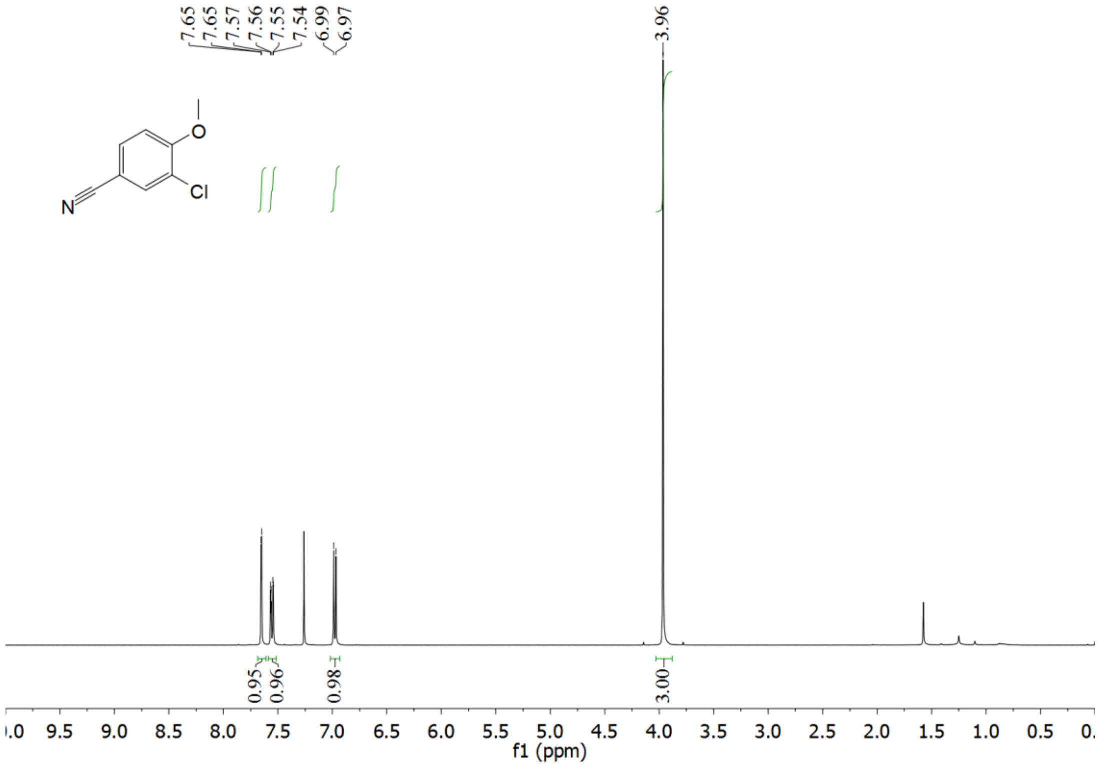

^{1}H NMR (400 MHz, $CDCl_3$) of **2m**

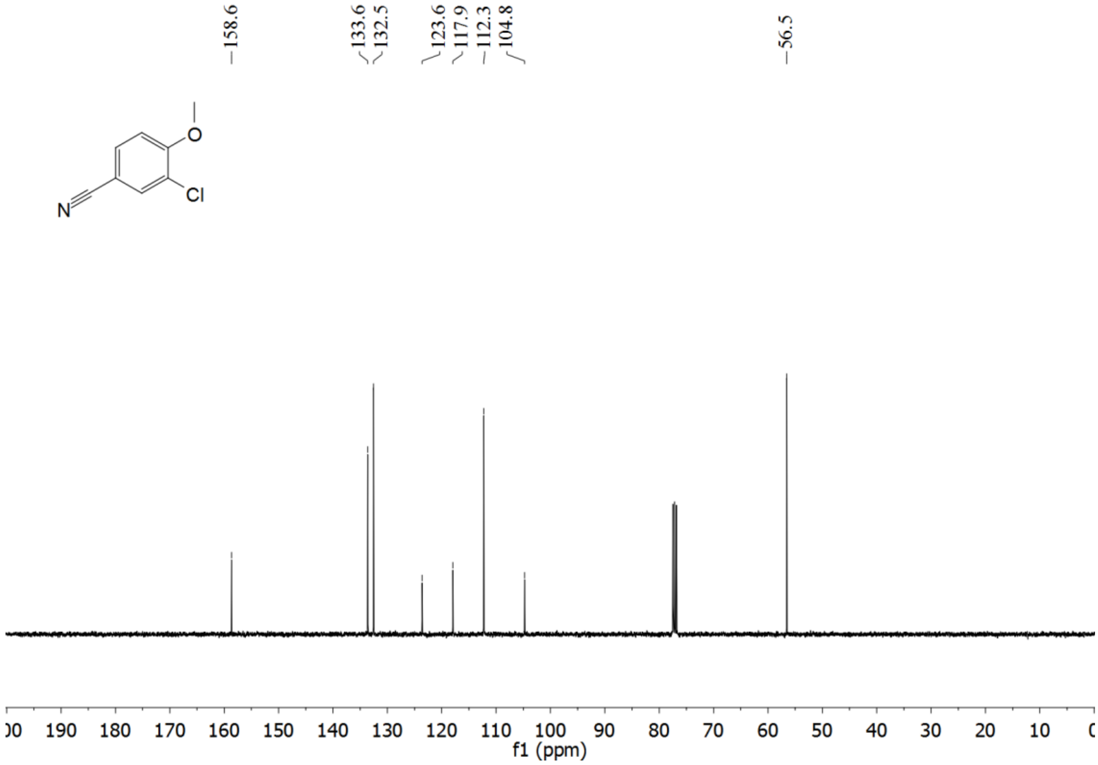

^{13}C NMR (101 MHz, $CDCl_3$) of **2m**

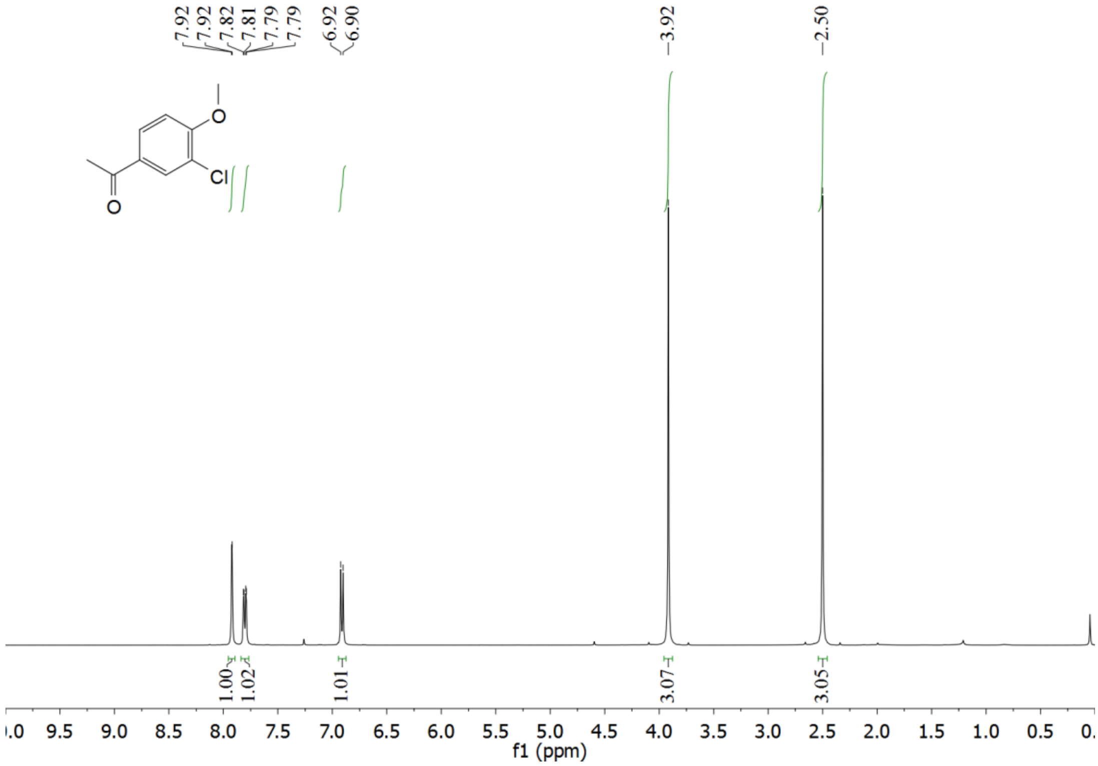

^{1}H NMR (400 MHz, $CDCl_3$) of **2n**

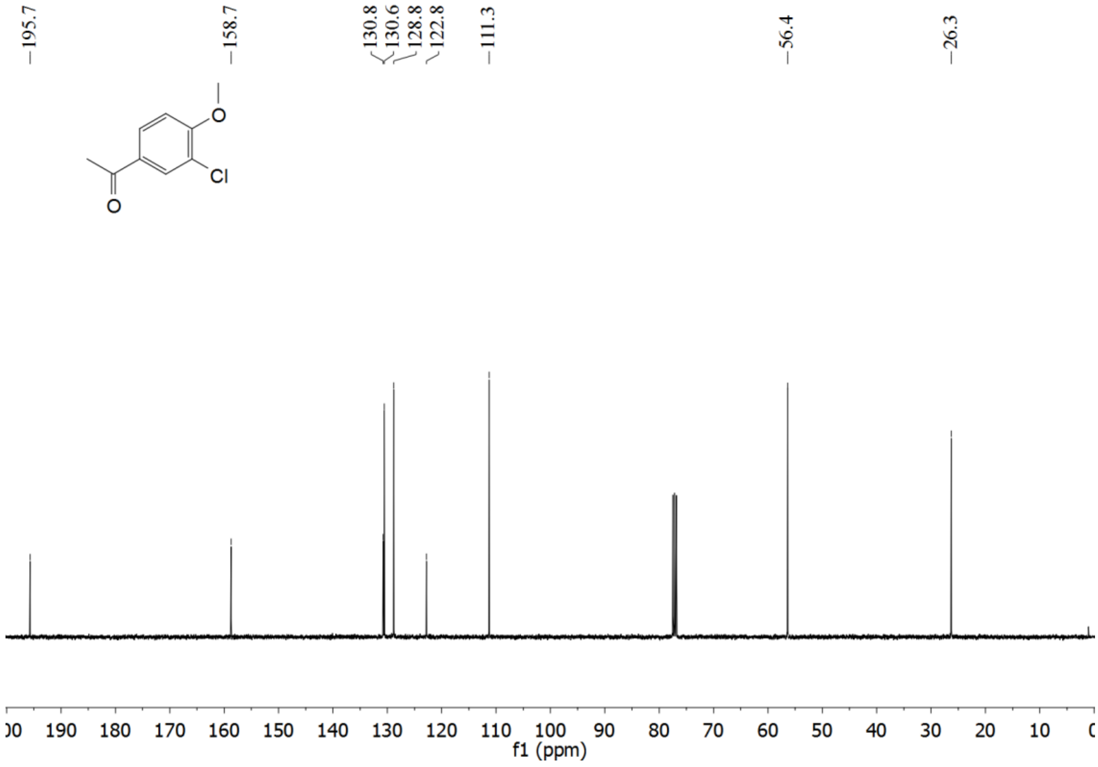

^{13}C NMR (101 MHz, $CDCl_3$) of **2n**

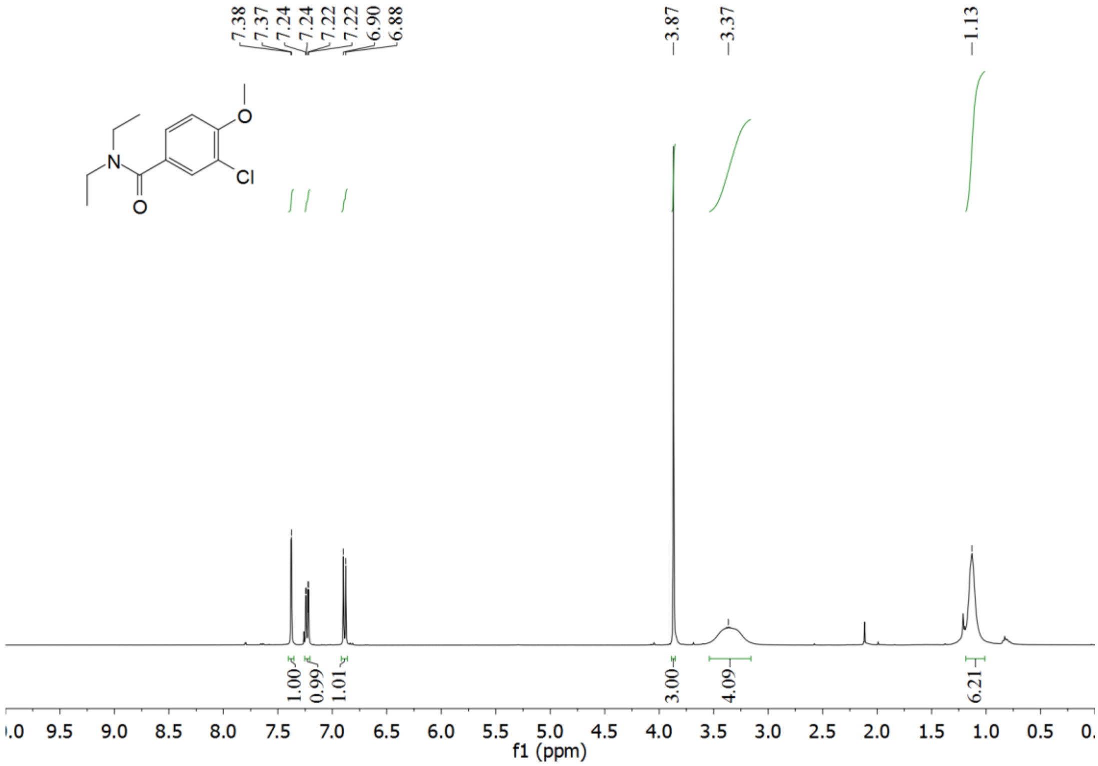

^{1}H NMR (400 MHz, $CDCl_3$) of **2o**

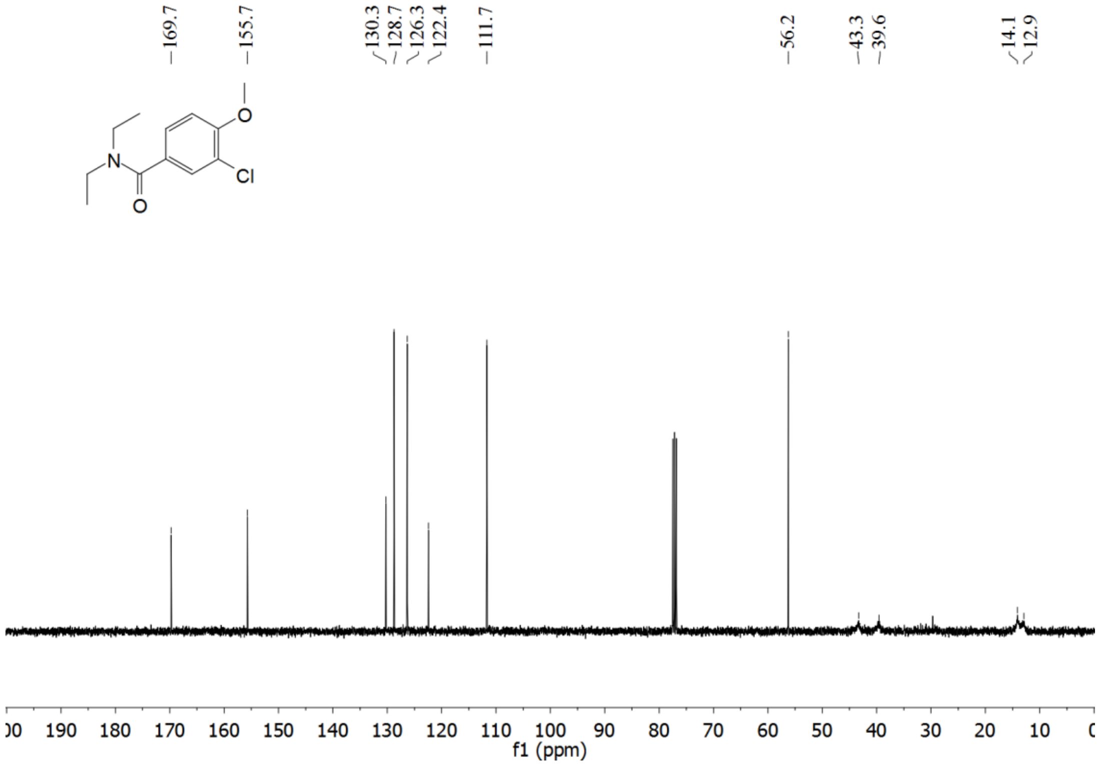

^{13}C NMR (101 MHz, $CDCl_3$) of **2o**

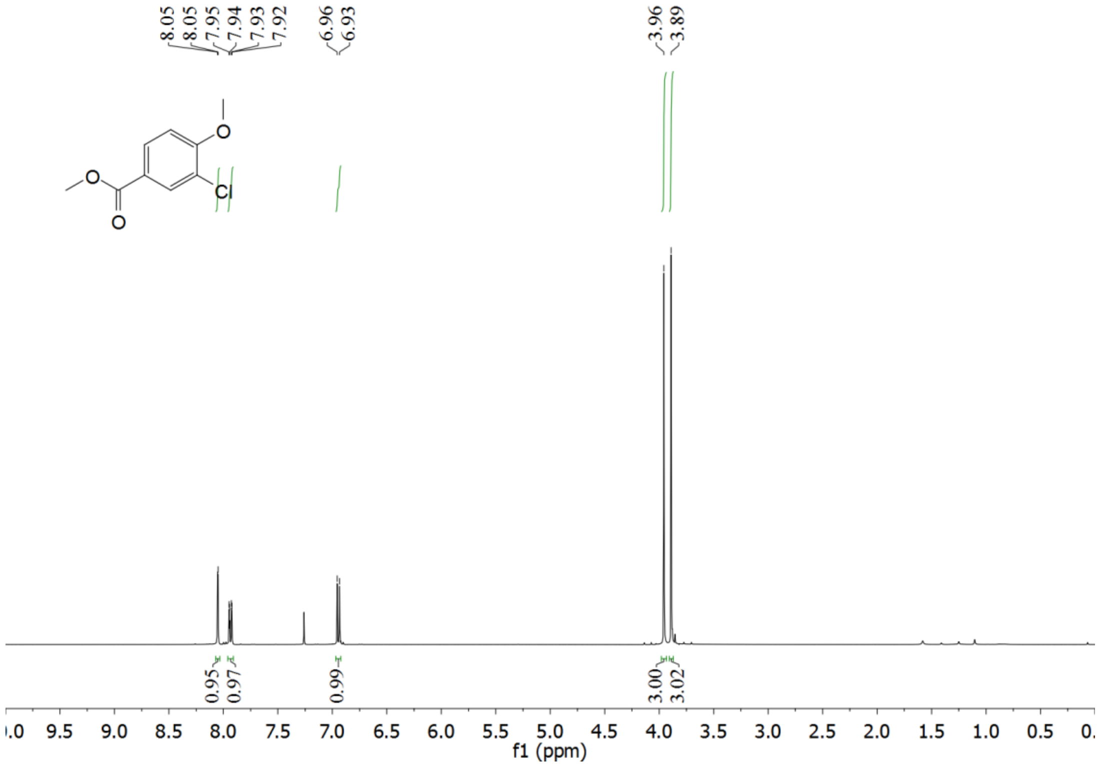

^{1}H NMR (400 MHz, $CDCl_3$) of **2p**

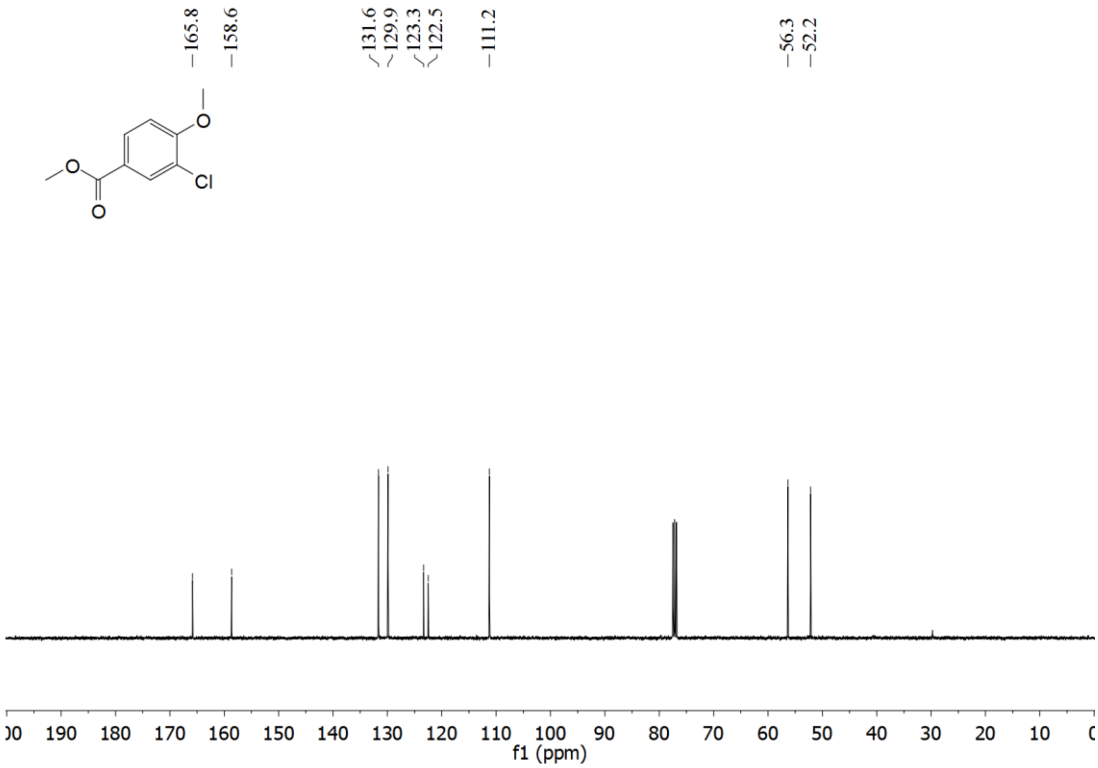

^{13}C NMR (101 MHz, $CDCl_3$) of **2p**

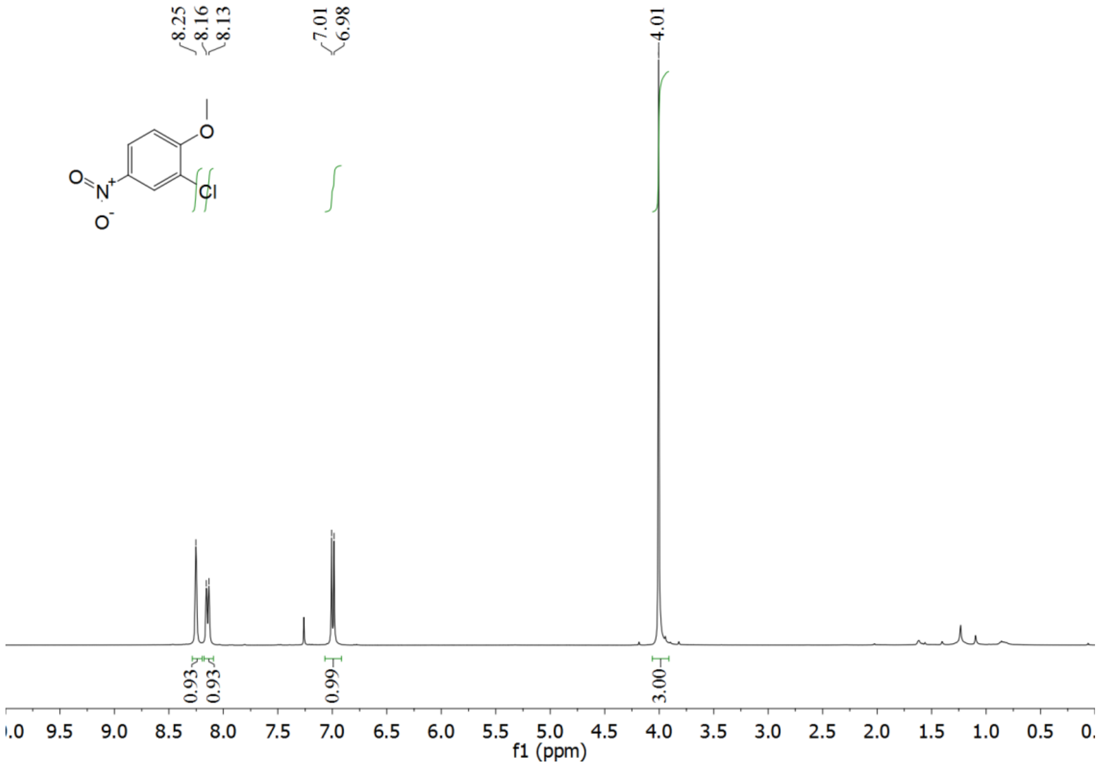

^{1}H NMR (400 MHz, $CDCl_3$) of **2q**

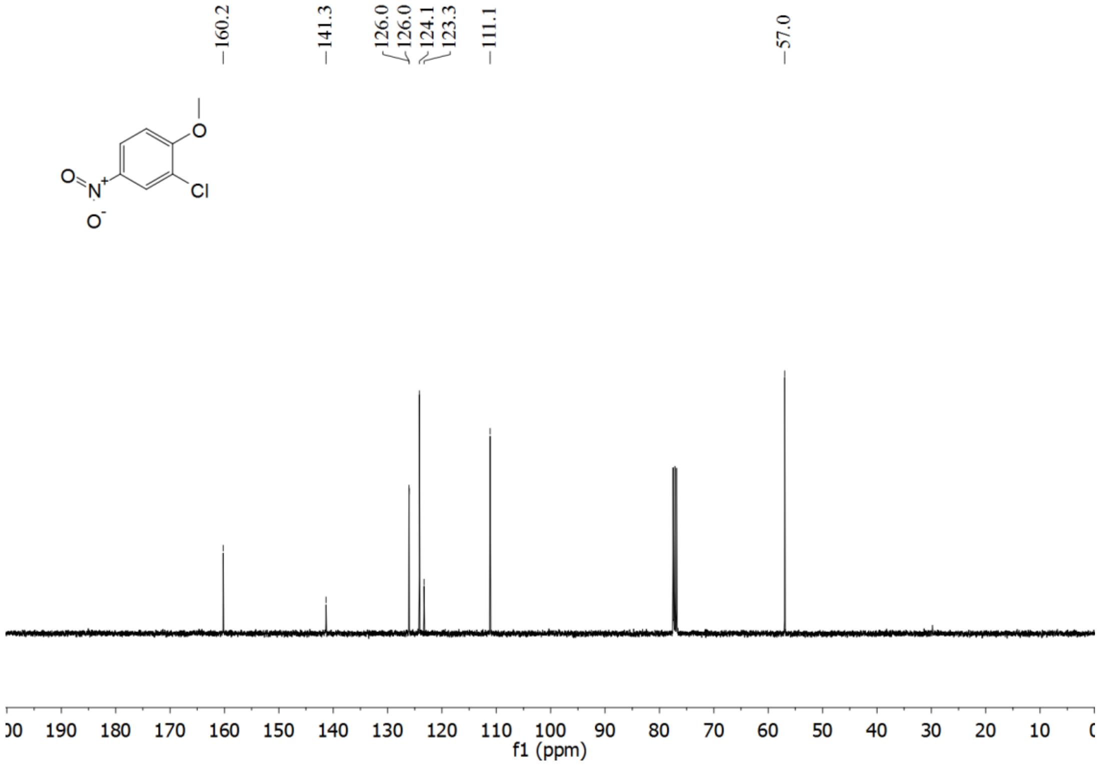

^{13}C NMR (101 MHz, $CDCl_3$) of **2q**

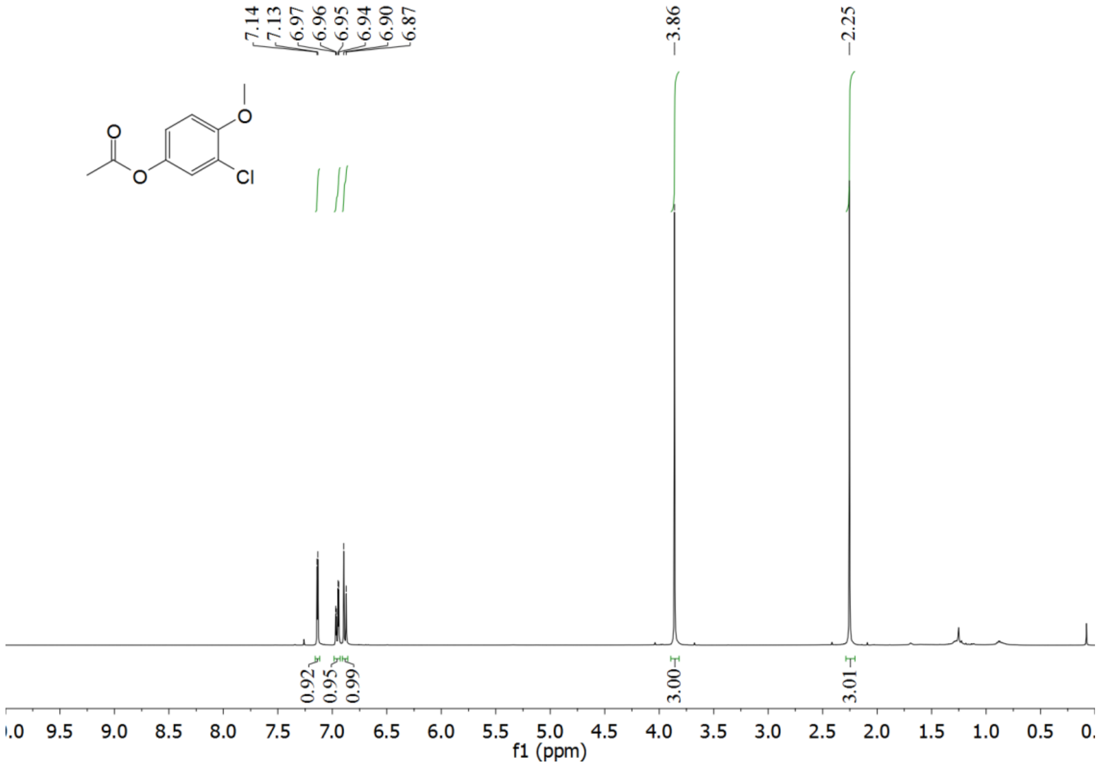

^{1}H NMR (400 MHz, $CDCl_3$) of **2r**

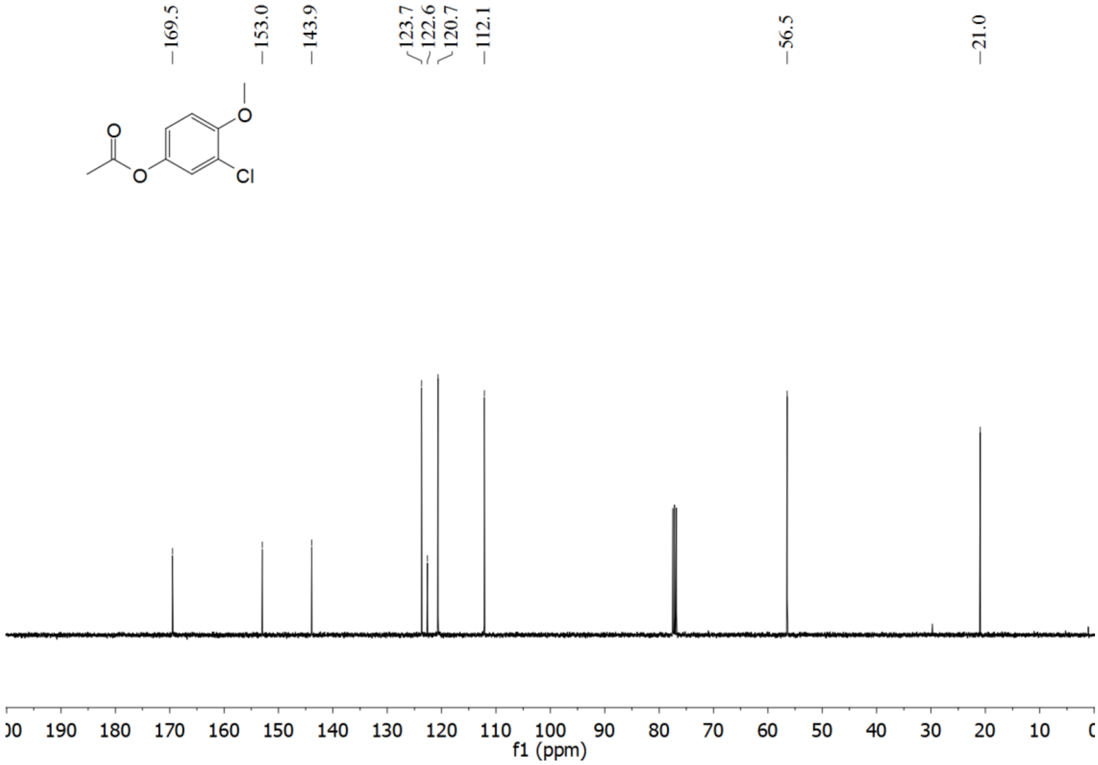

^{13}C NMR (101 MHz, $CDCl_3$) of **2r**

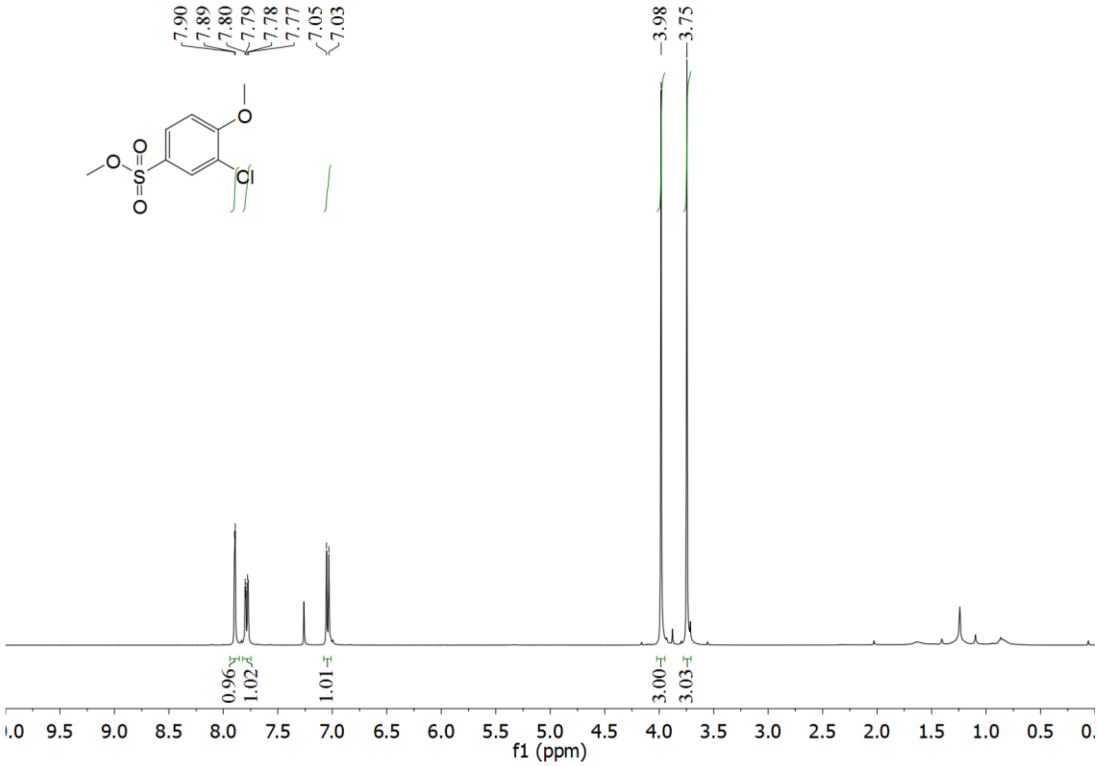

^{1}H NMR (400 MHz, $CDCl_3$) of **2s**

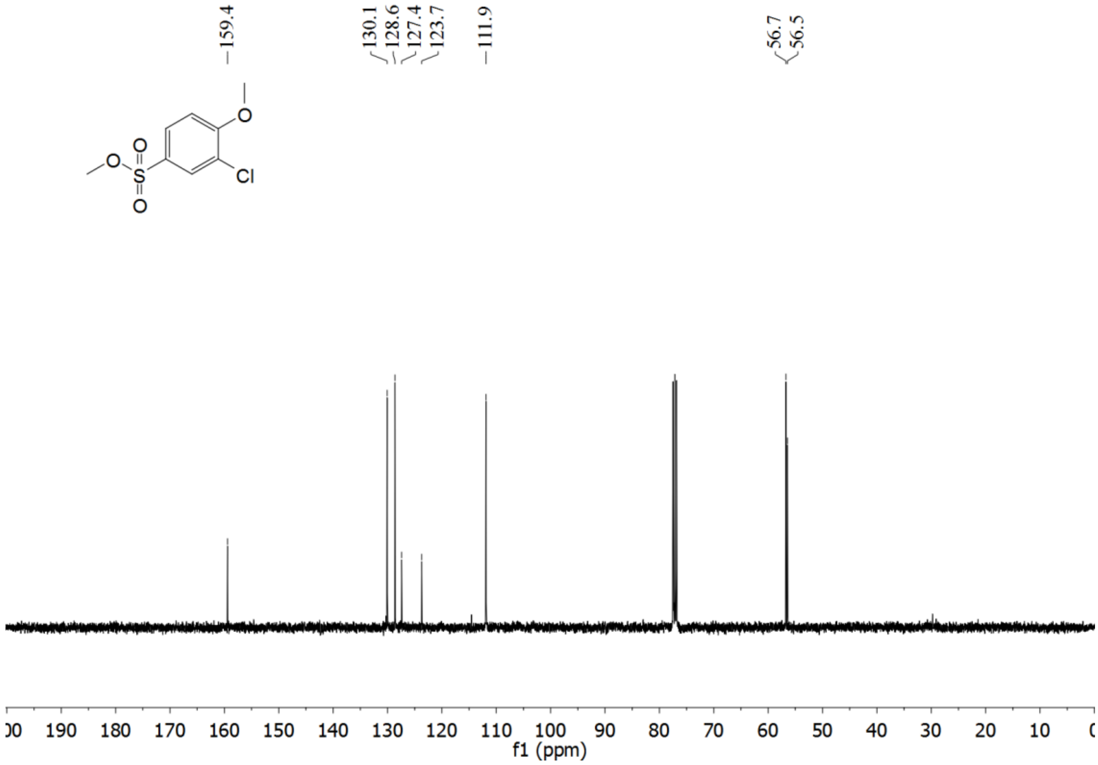

^{13}C NMR (101 MHz, $CDCl_3$) of **2s**

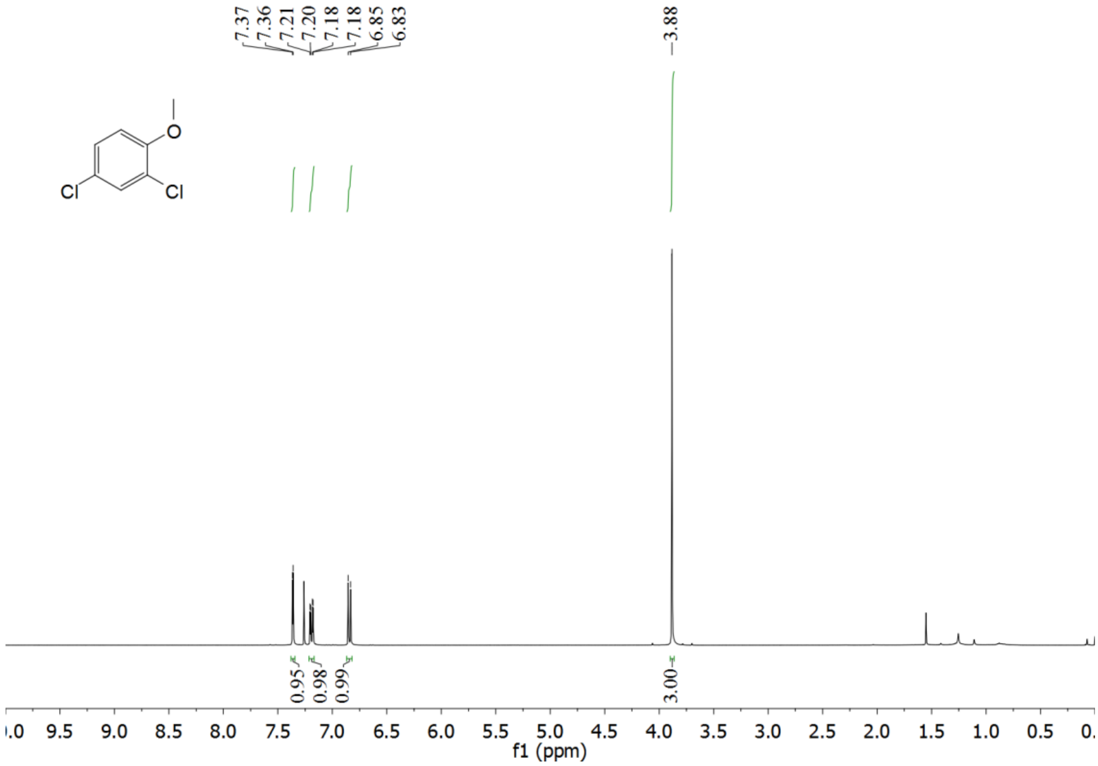

^{1}H NMR (400 MHz, $CDCl_3$) of **2t**

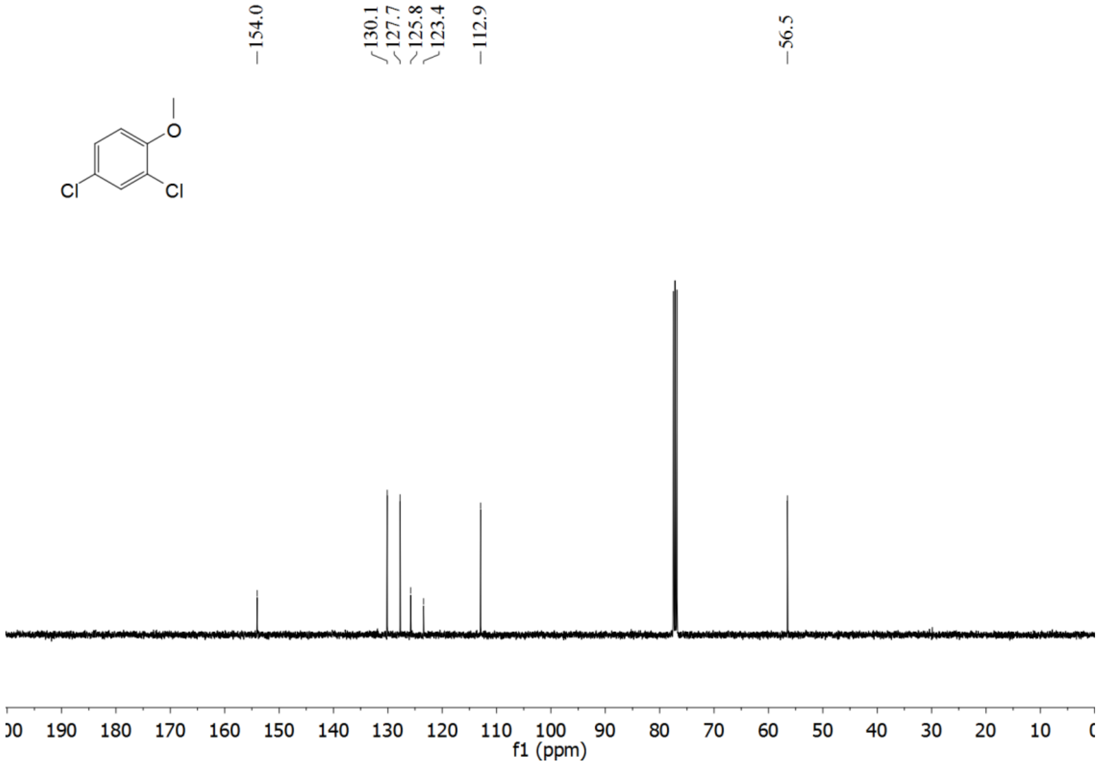

^{13}C NMR (101 MHz, $CDCl_3$) of **2t**

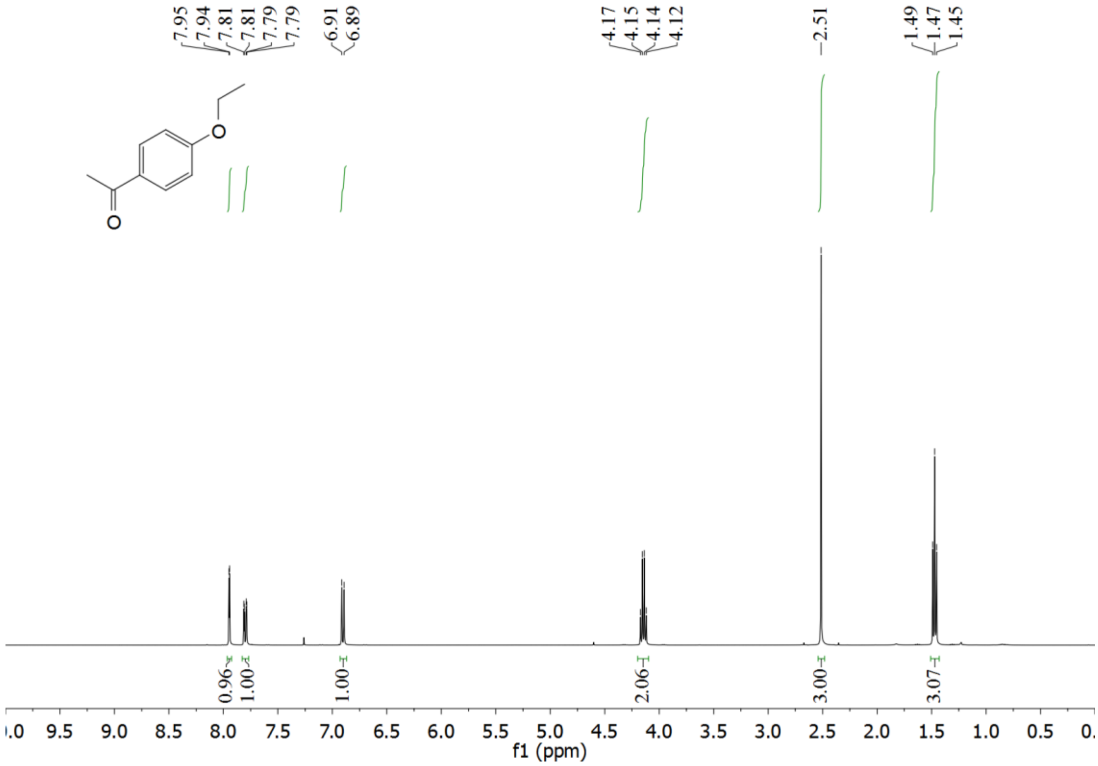

^1H NMR (400 MHz, $CDCl_3$) of **2u**

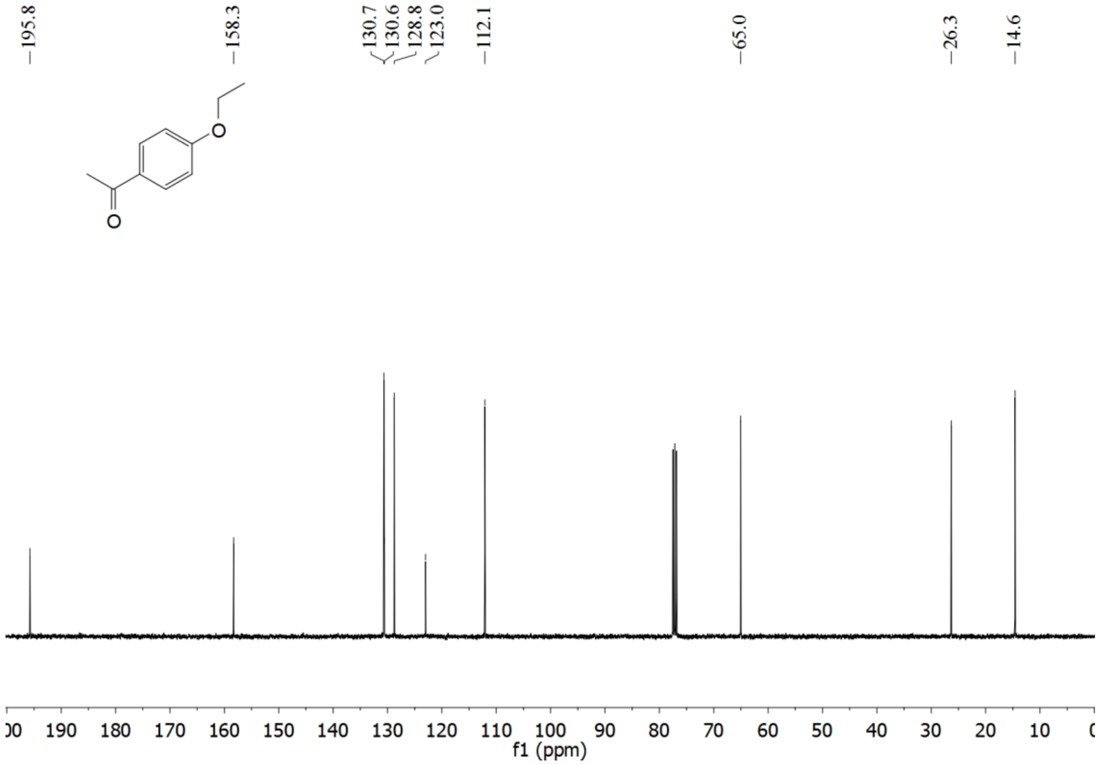

^{13}C NMR (101 MHz, $CDCl_3$) of **2u**

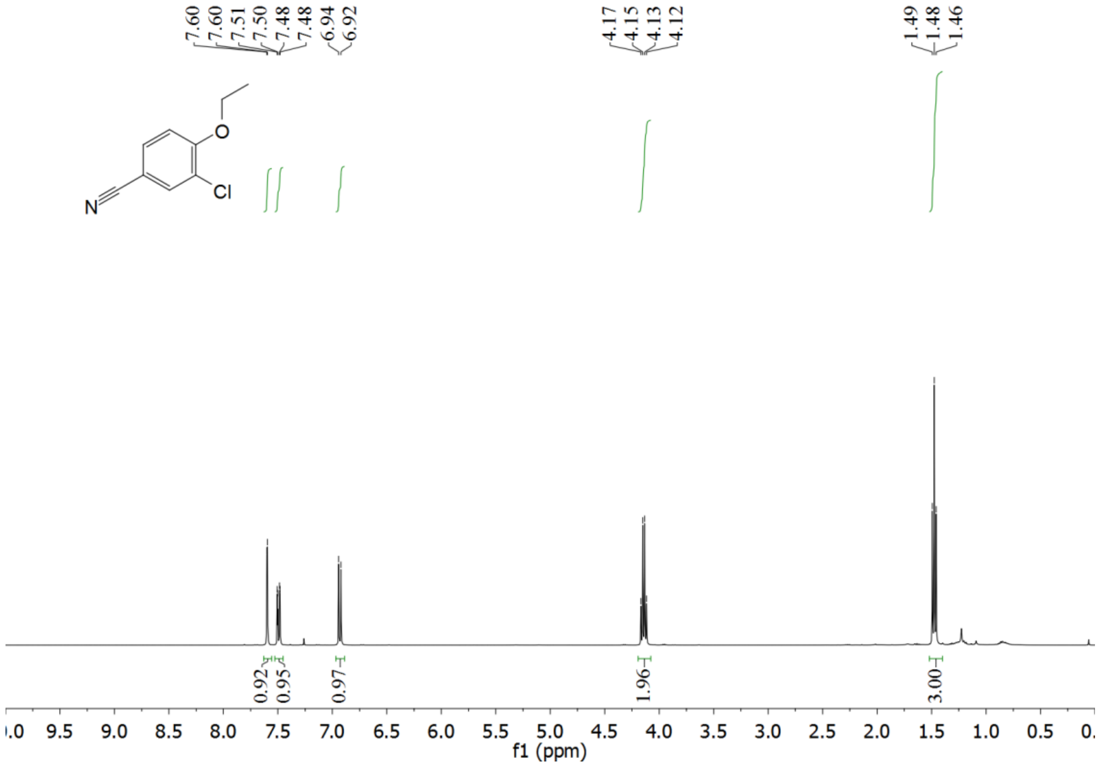

^{1}H NMR (400 MHz, $CDCl_3$) of **2v**

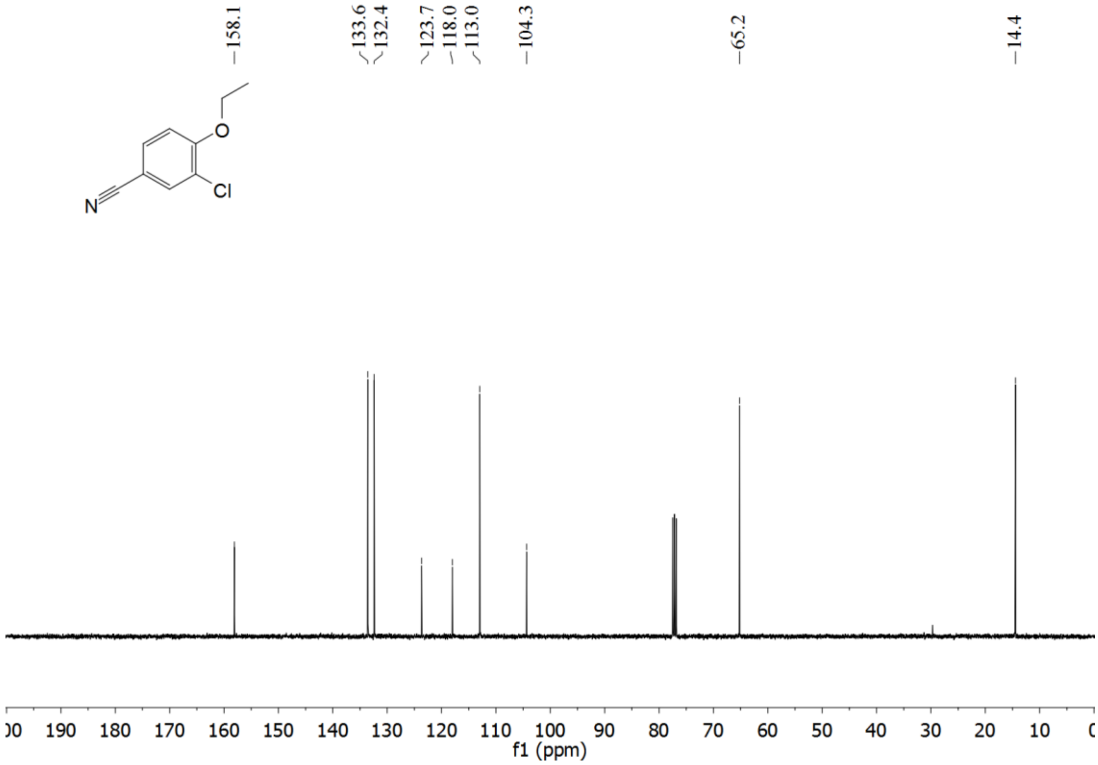

^{13}C NMR (101 MHz, $CDCl_3$) of **2v**

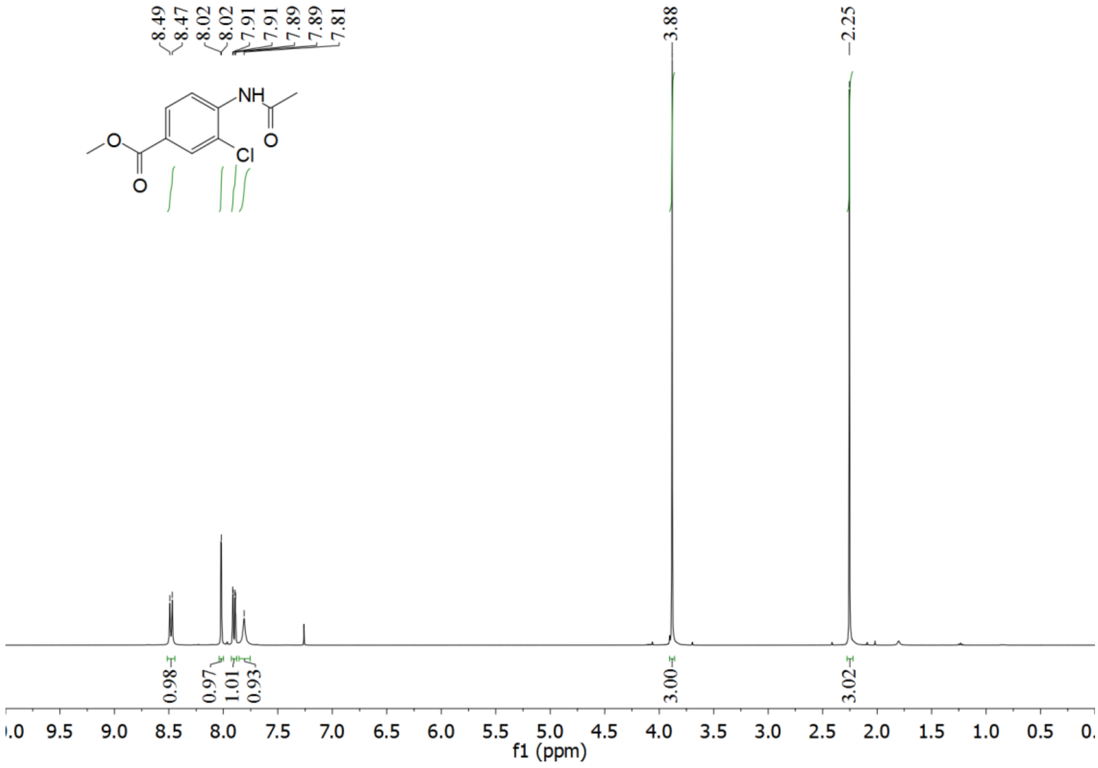

^{1}H NMR (400 MHz, $CDCl_3$) of **2w**

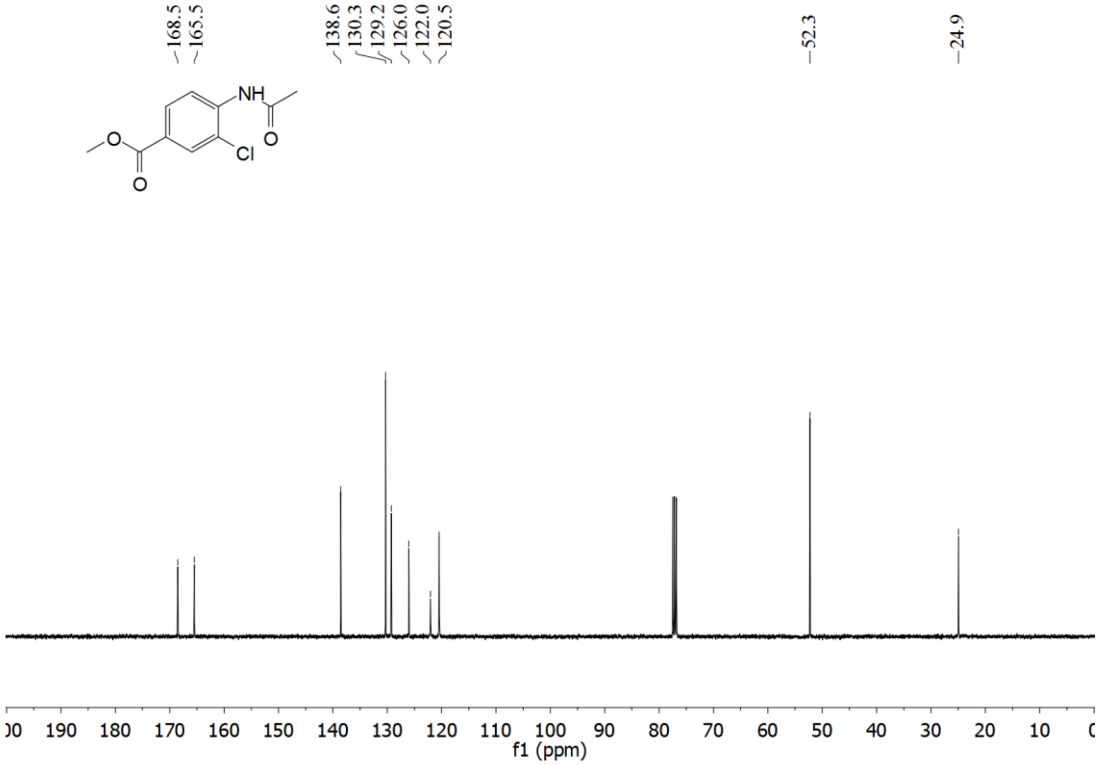

^{13}C NMR (101 MHz, $CDCl_3$) of **2w**

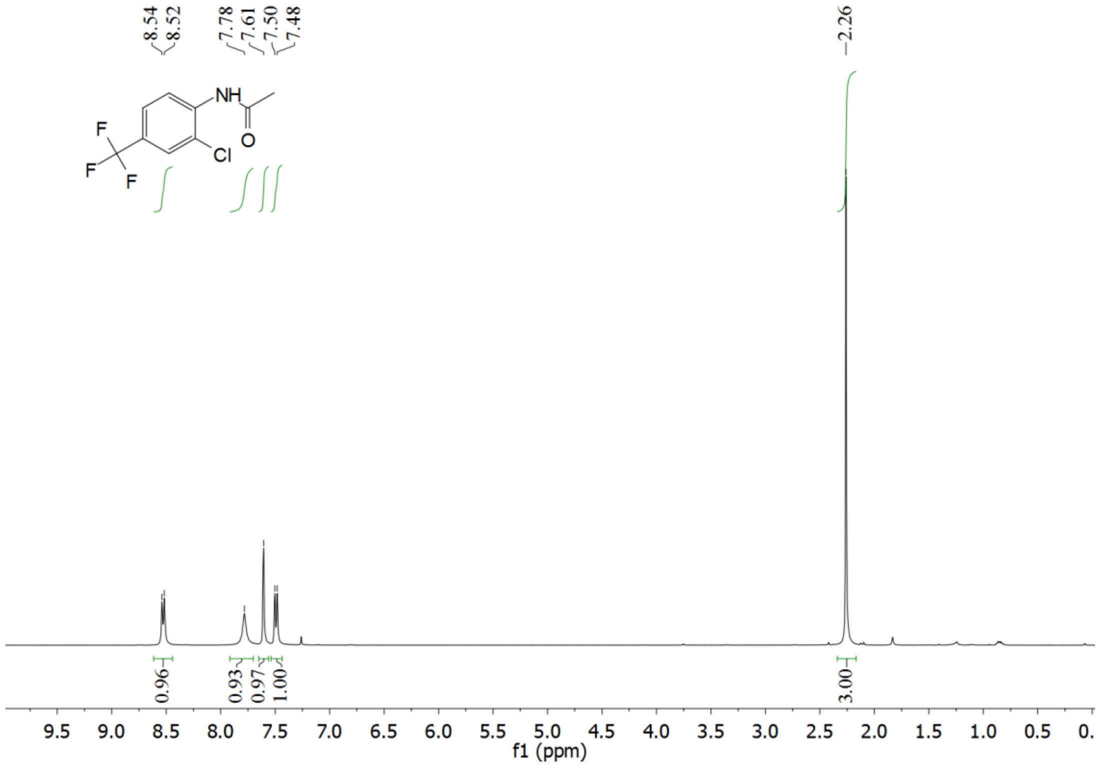

^{1}H NMR (400 MHz, $CDCl_3$) of **2x**

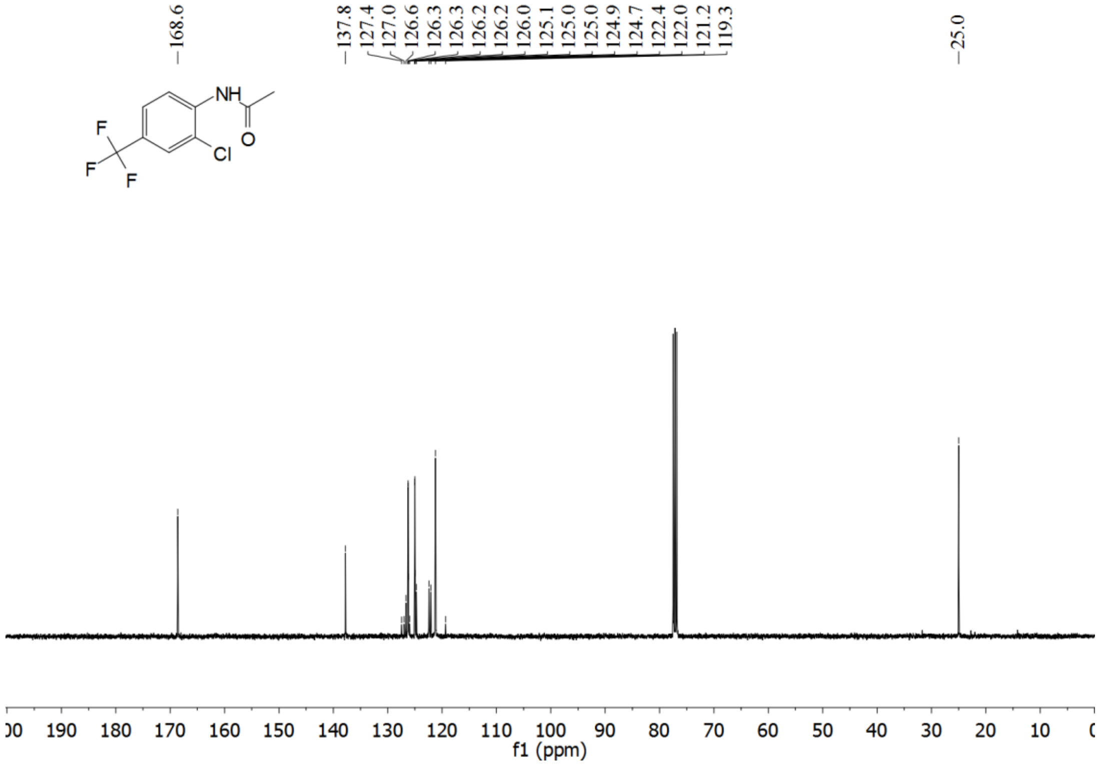

^{13}C NMR (101 MHz, $CDCl_3$) of **2x**

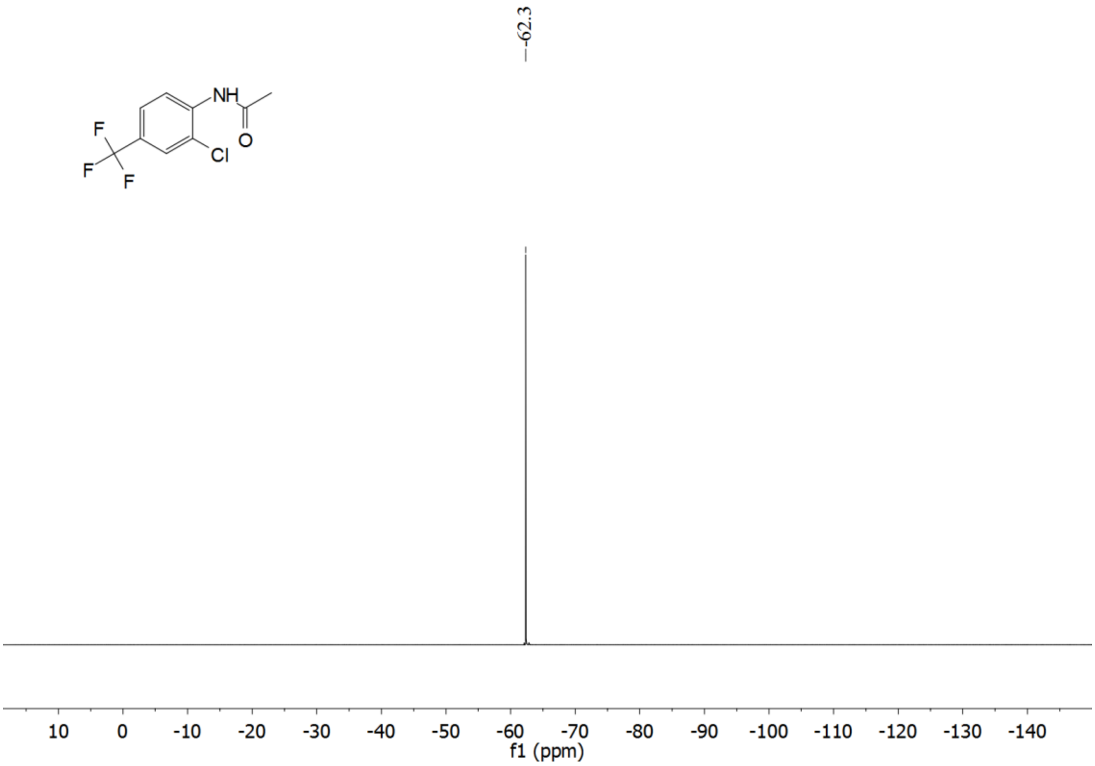

^{1}F NMR (376 MHz, $CDCl_3$) of **2x**

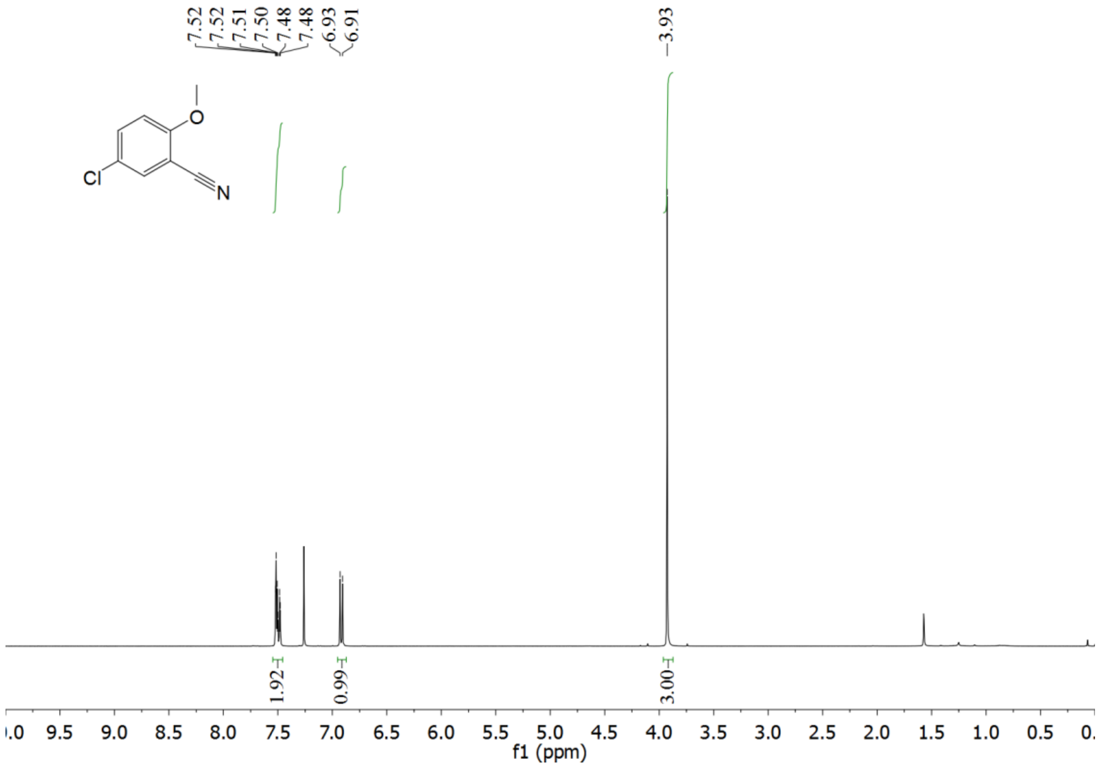

^{1}H NMR (400 MHz, $CDCl_3$) of **2y**

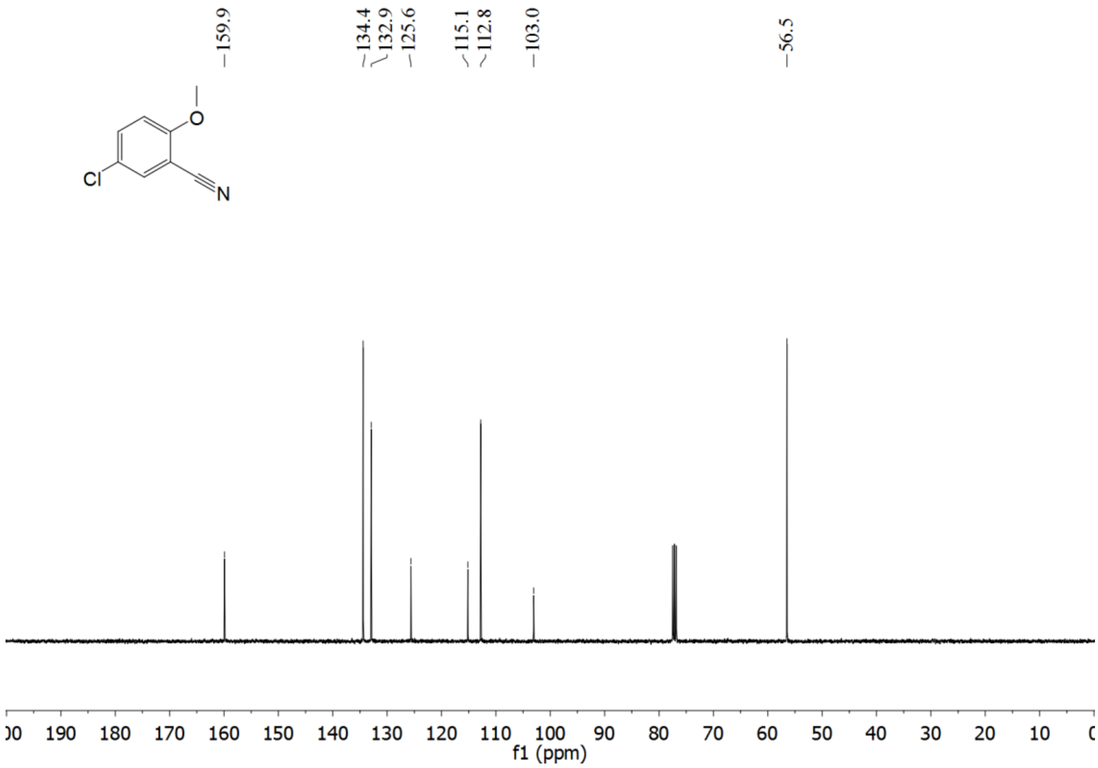

^{13}C NMR (101 MHz, $CDCl_3$) of **2y**

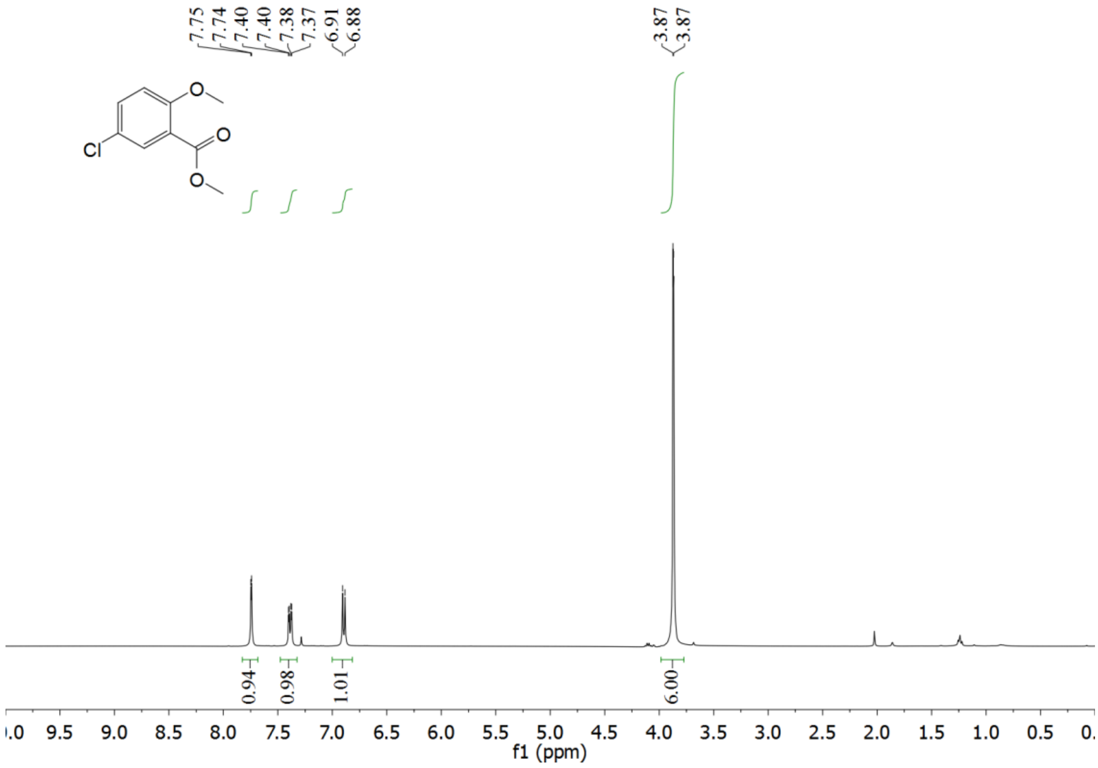

^{1}H NMR (400 MHz, $CDCl_3$) of **2z**

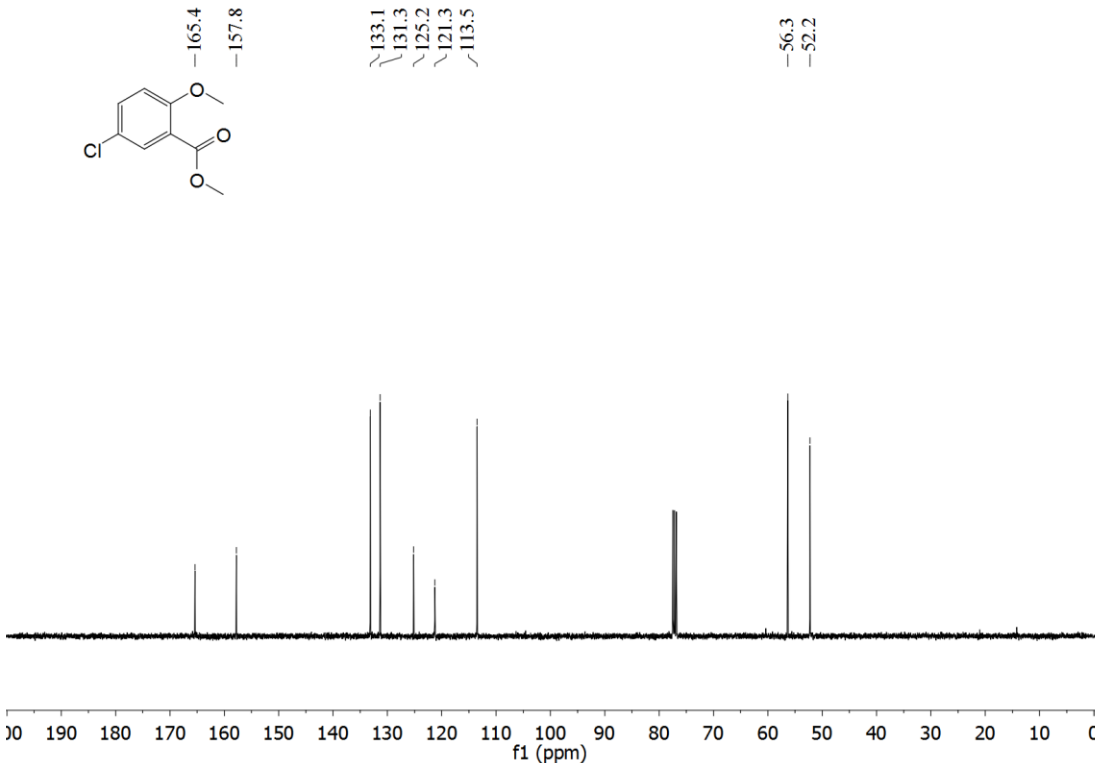

^{13}C NMR (101 MHz, $CDCl_3$) of **2z**

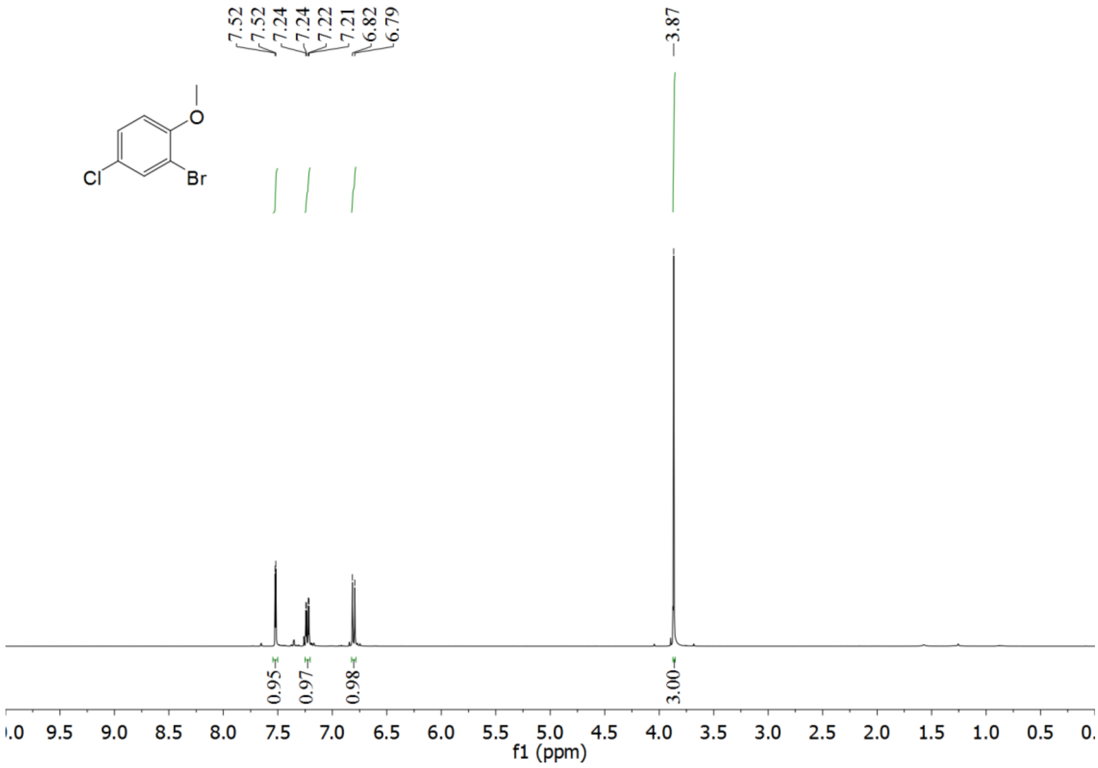

^{1}H NMR (400 MHz, $CDCl_3$) of **2aa**

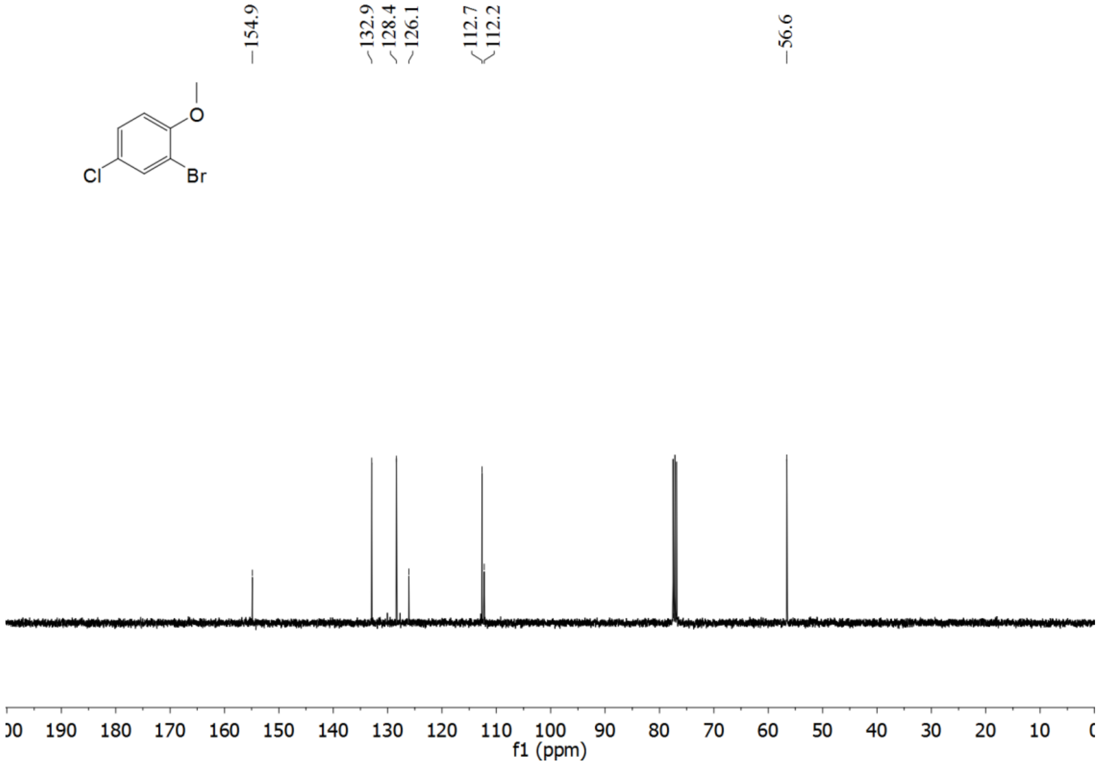

^{13}C NMR (101 MHz, $CDCl_3$) of **2aa**

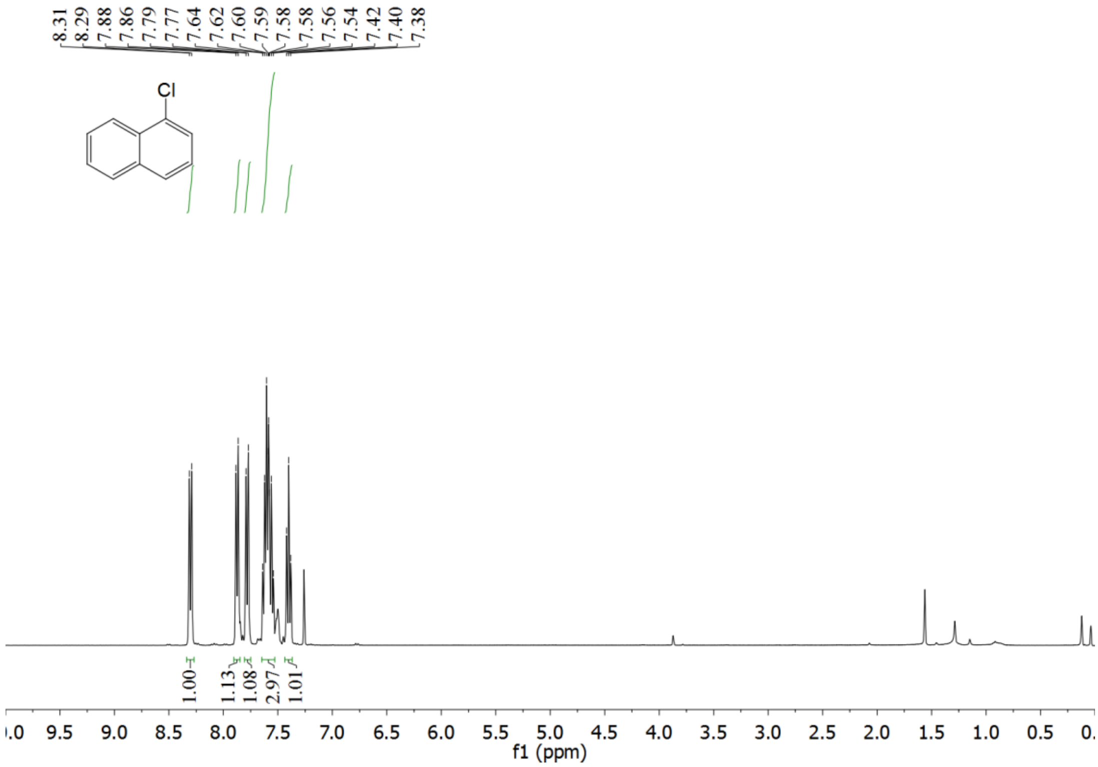

^{1}H NMR (400 MHz, $CDCl_3$) of **2ab**

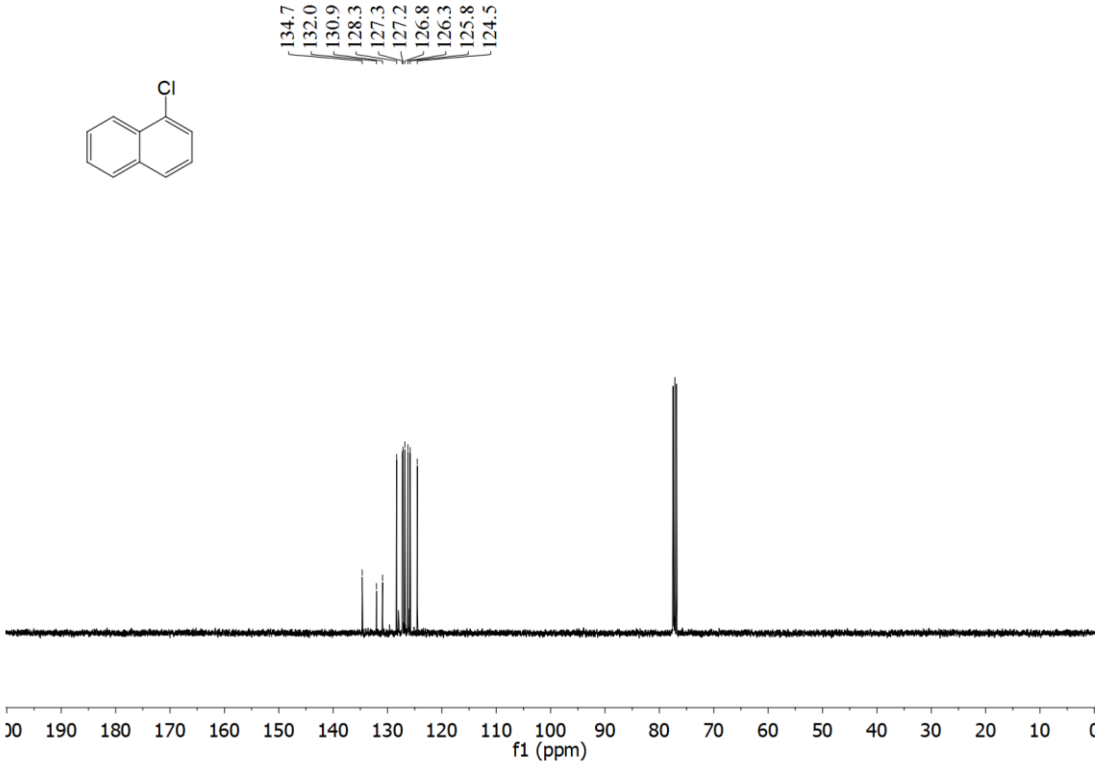

^{13}C NMR (101 MHz, $CDCl_3$) of **2ab**

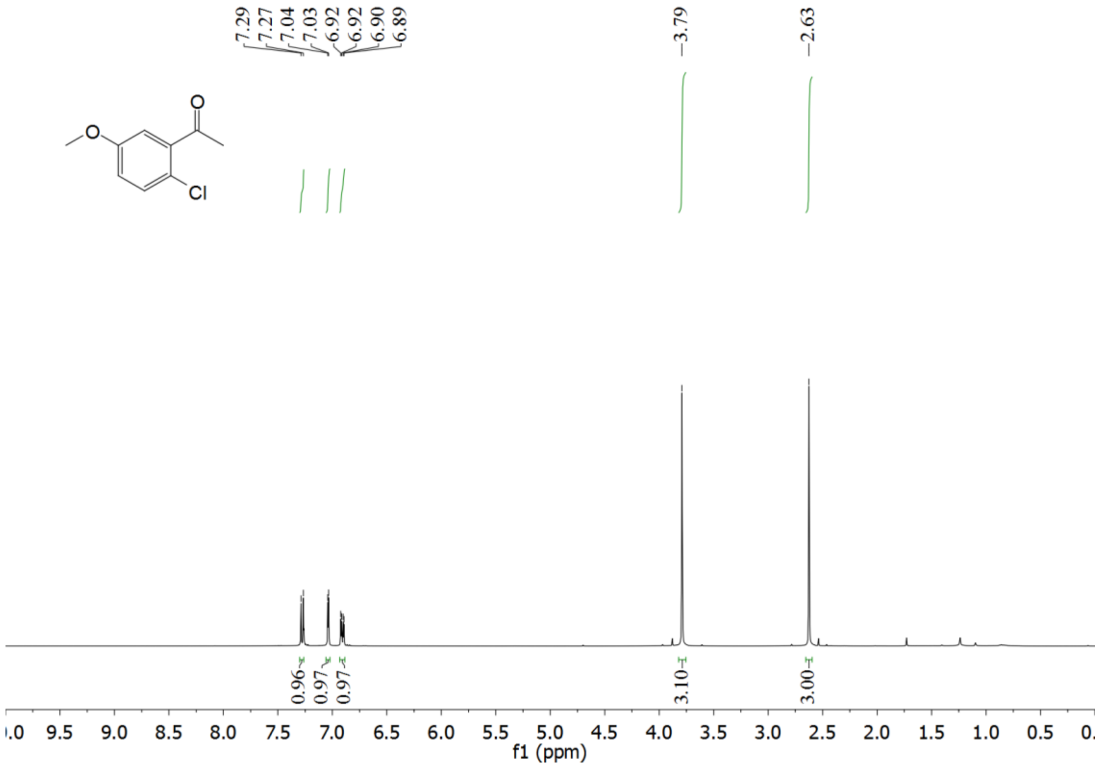

^{1}H NMR (400 MHz, $CDCl_3$) of **2ac**

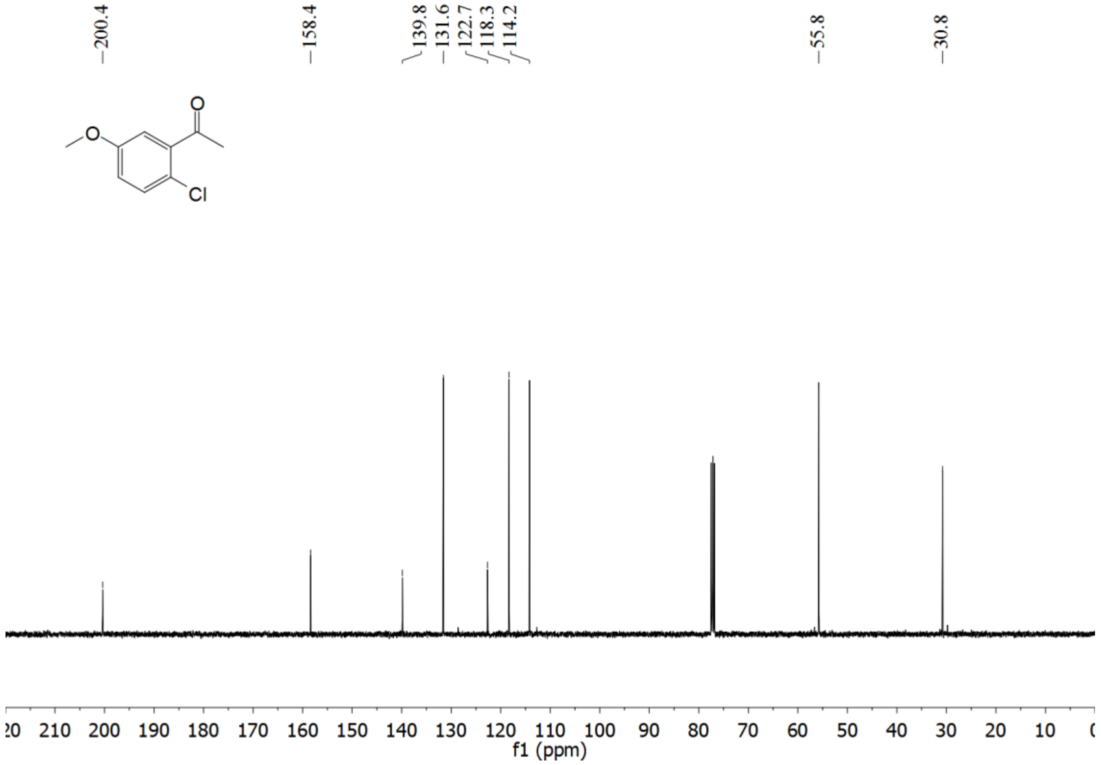

^{13}C NMR (101 MHz, $CDCl_3$) of **2ac**

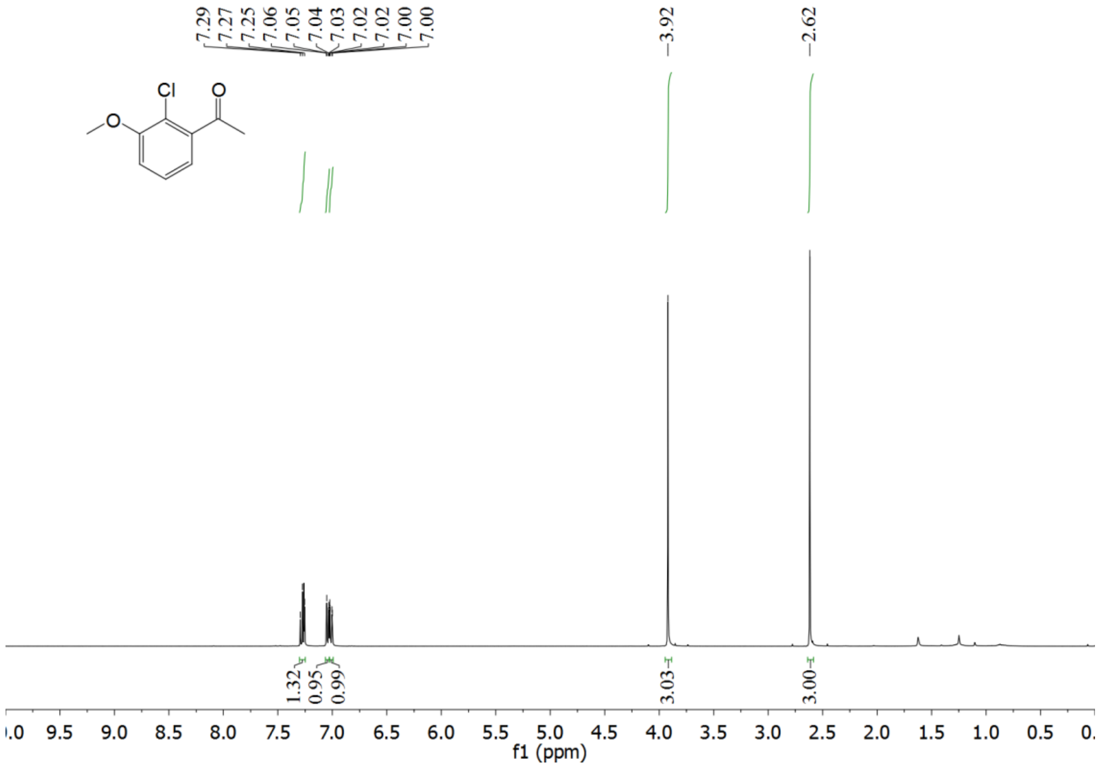

^{1}H NMR (400 MHz, $CDCl_3$) of **2ac'**

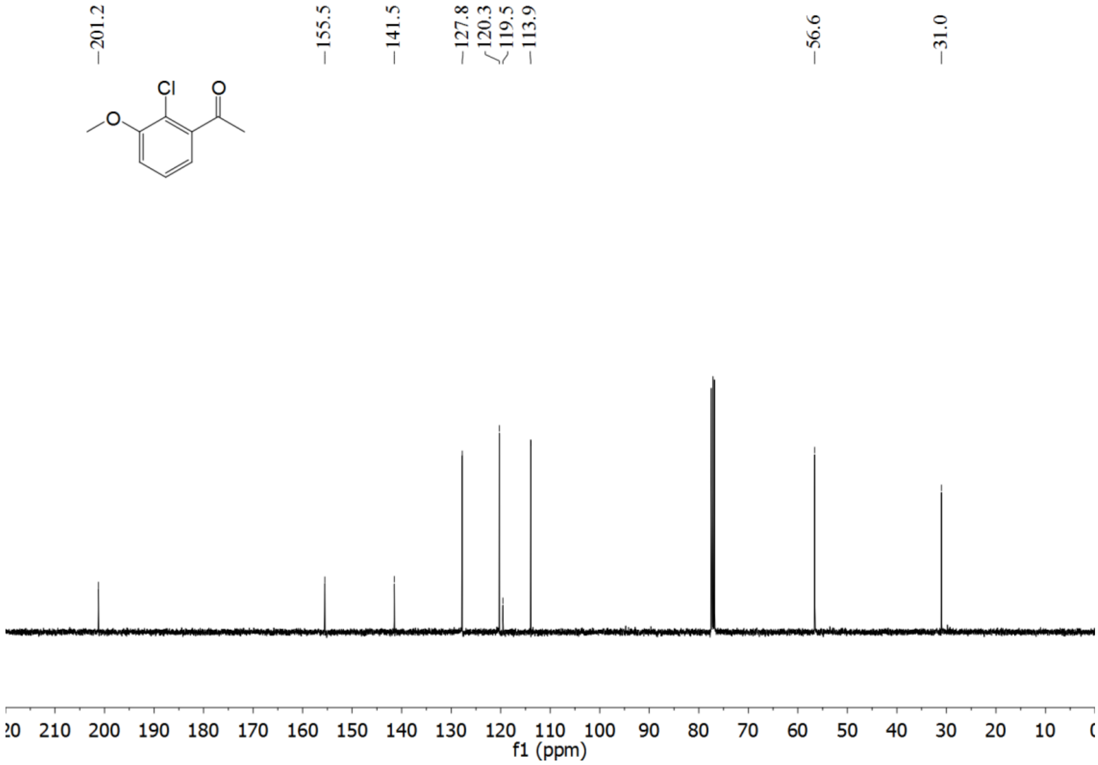

^{13}C NMR (101 MHz, $CDCl_3$) of **2ac'**

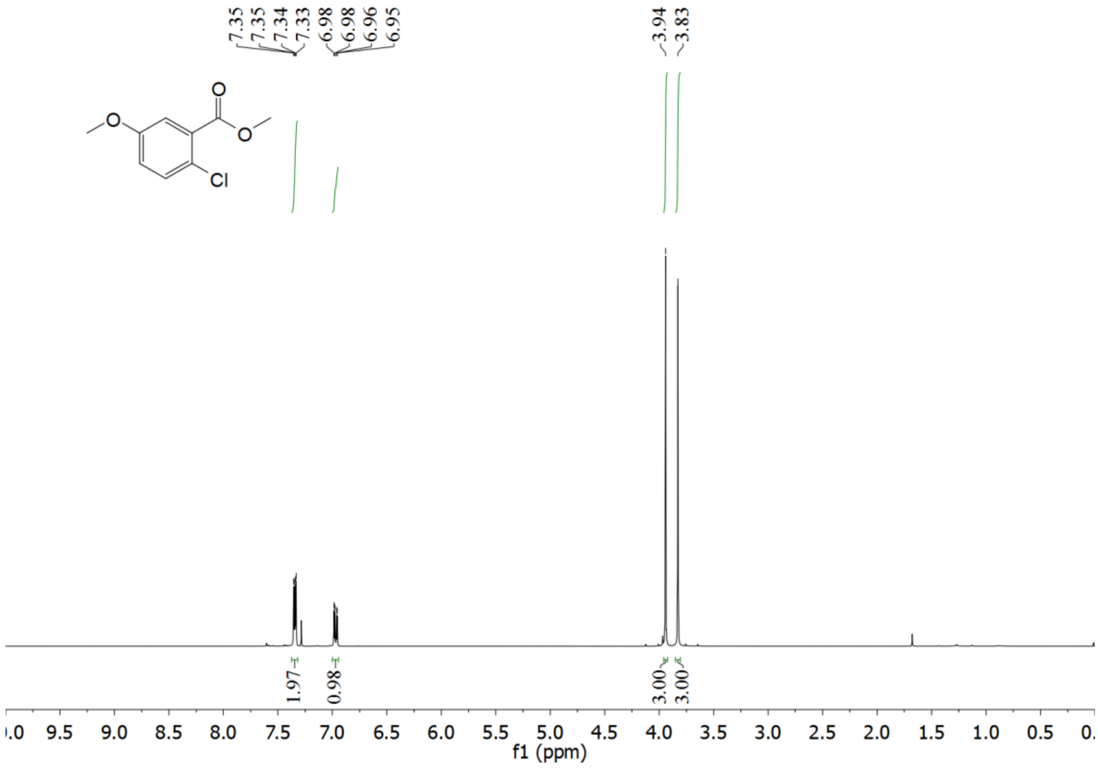

^{1}H NMR (400 MHz, $CDCl_3$) of **2ad**

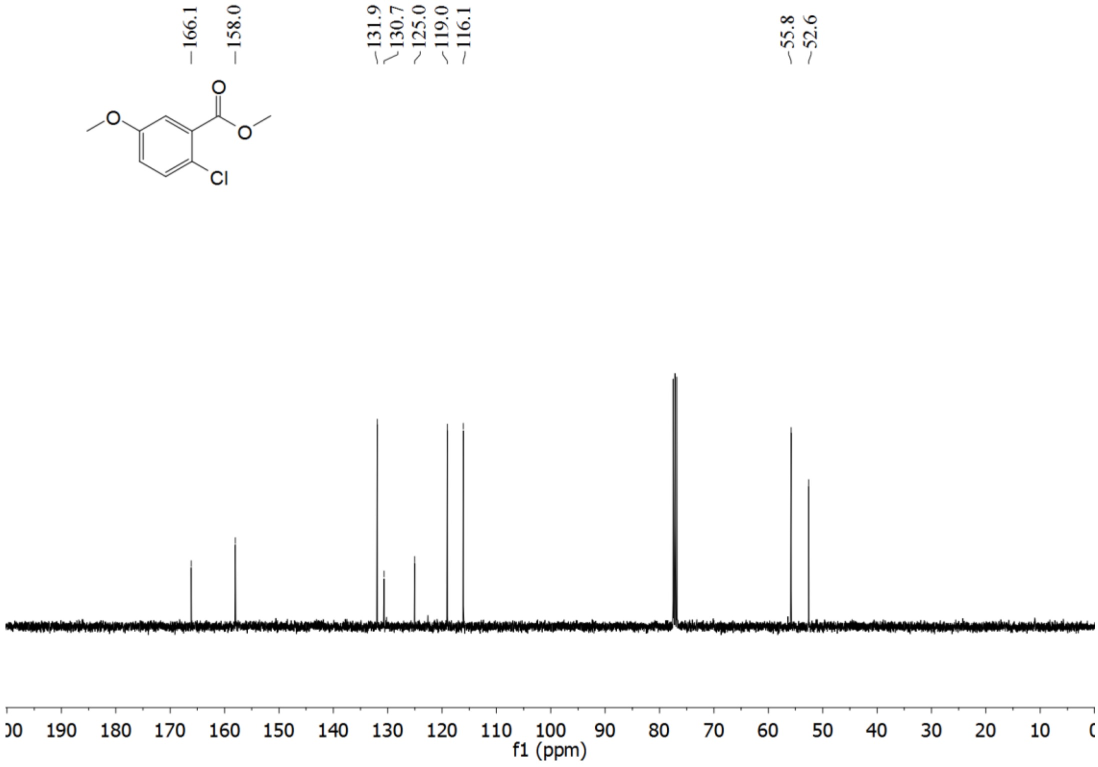

^{13}C NMR (101 MHz, $CDCl_3$) of **2ad**

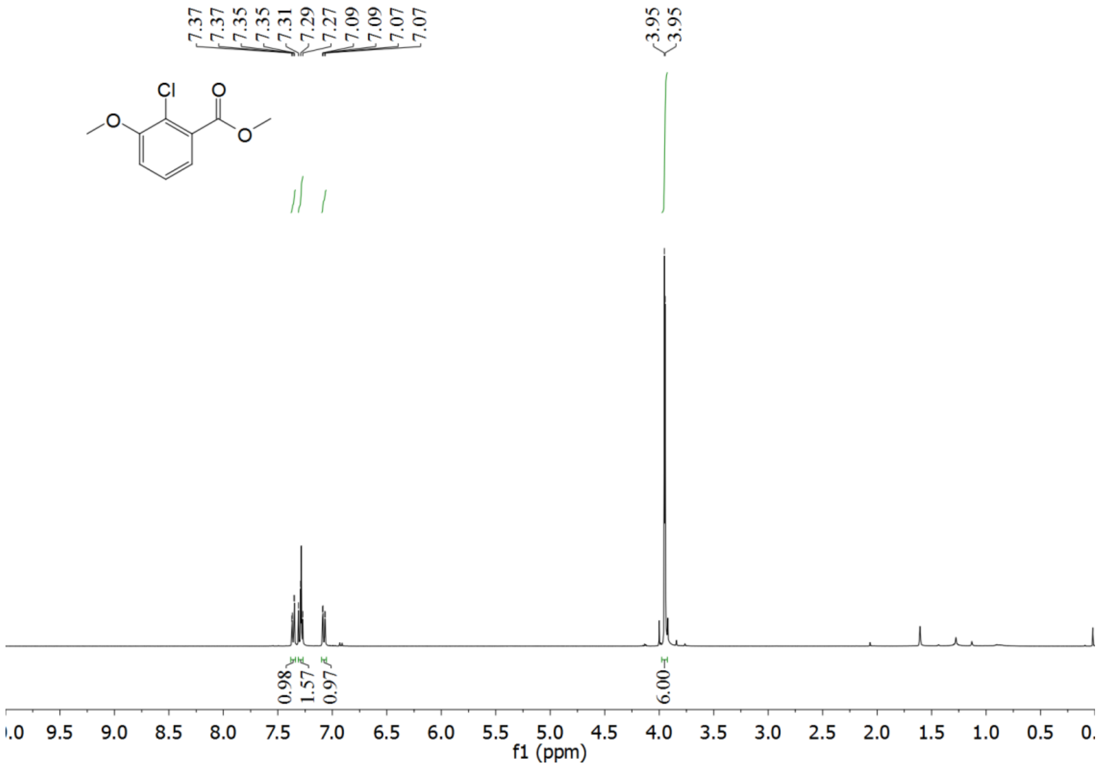

^{1}H NMR (400 MHz, $CDCl_3$) of **2ad’**

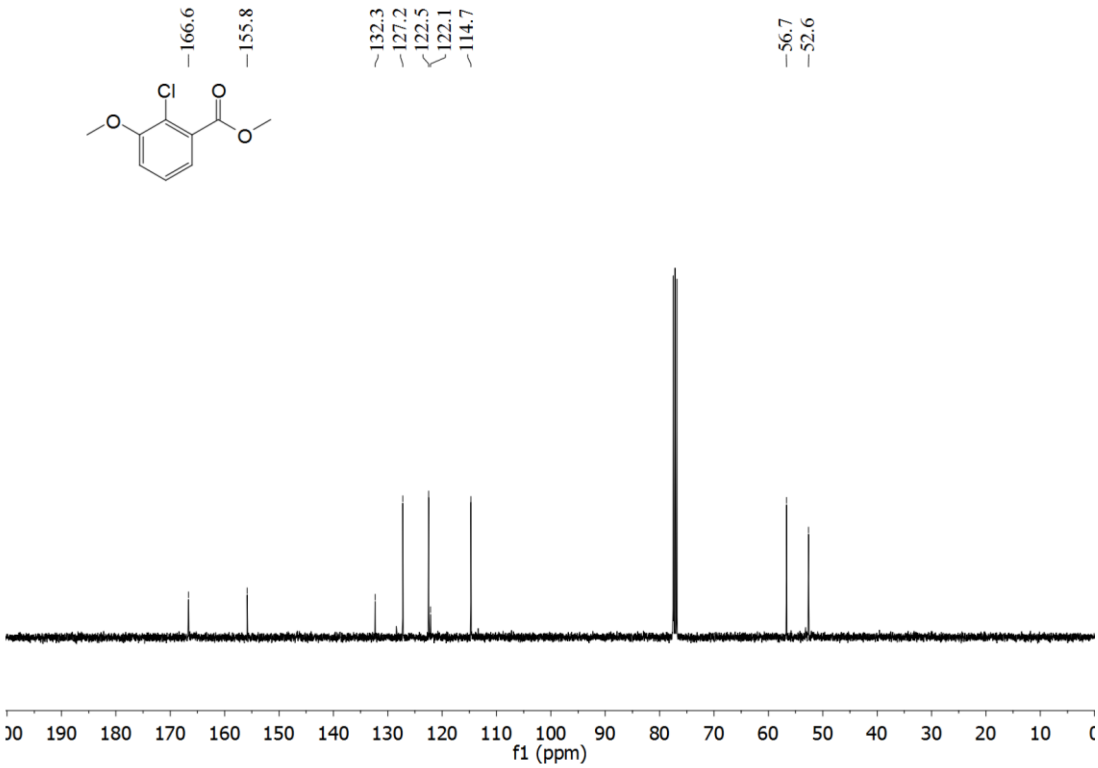

^{13}C NMR (101 MHz, $CDCl_3$) of **2ad’**

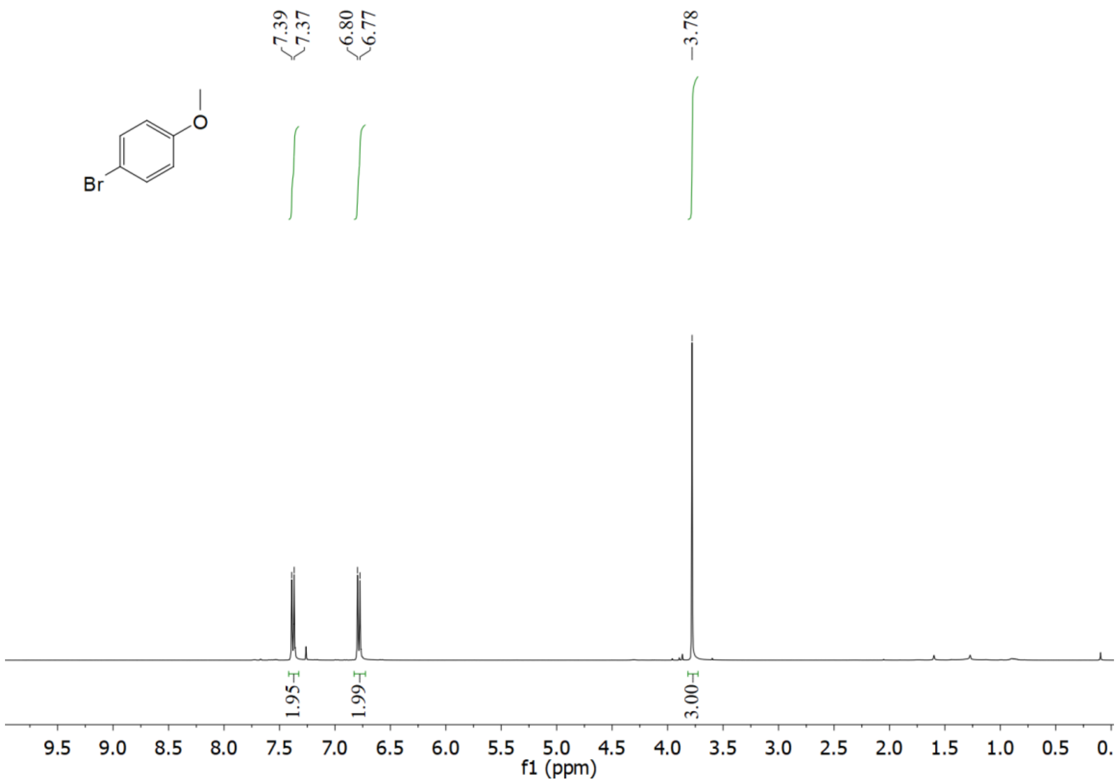

^{1}H NMR (400 MHz, $CDCl_3$) of **2af**

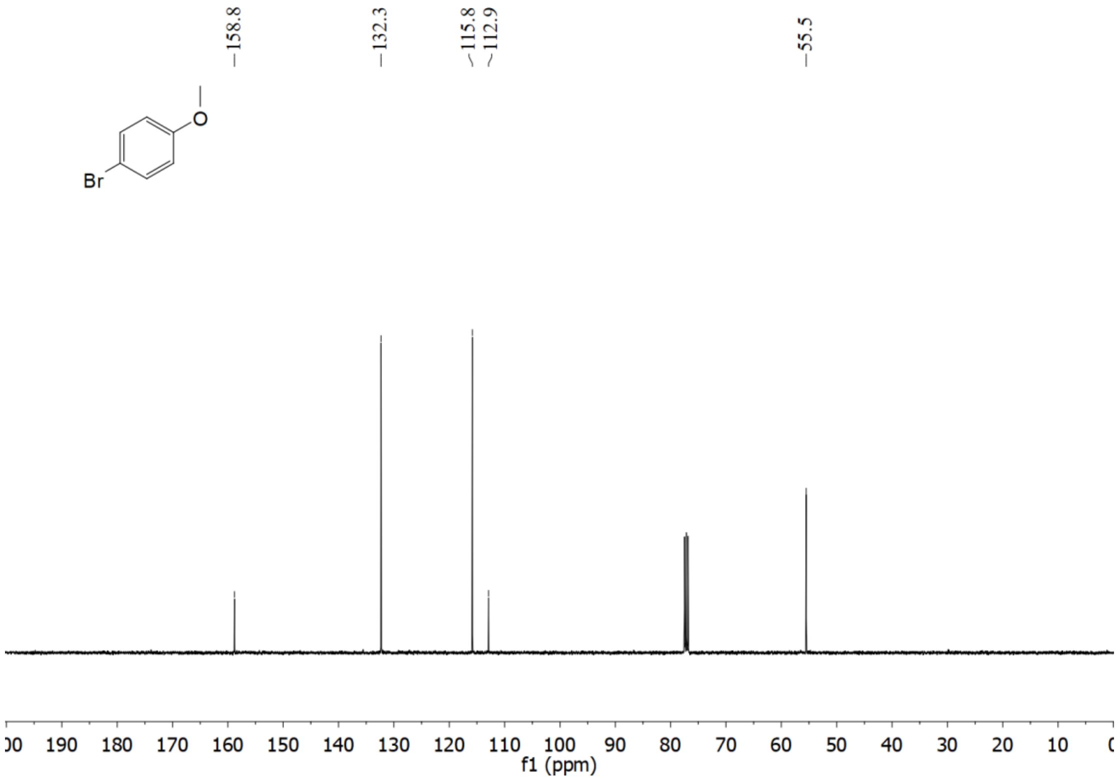

^{13}C NMR (101 MHz, $CDCl_3$) of **2af**

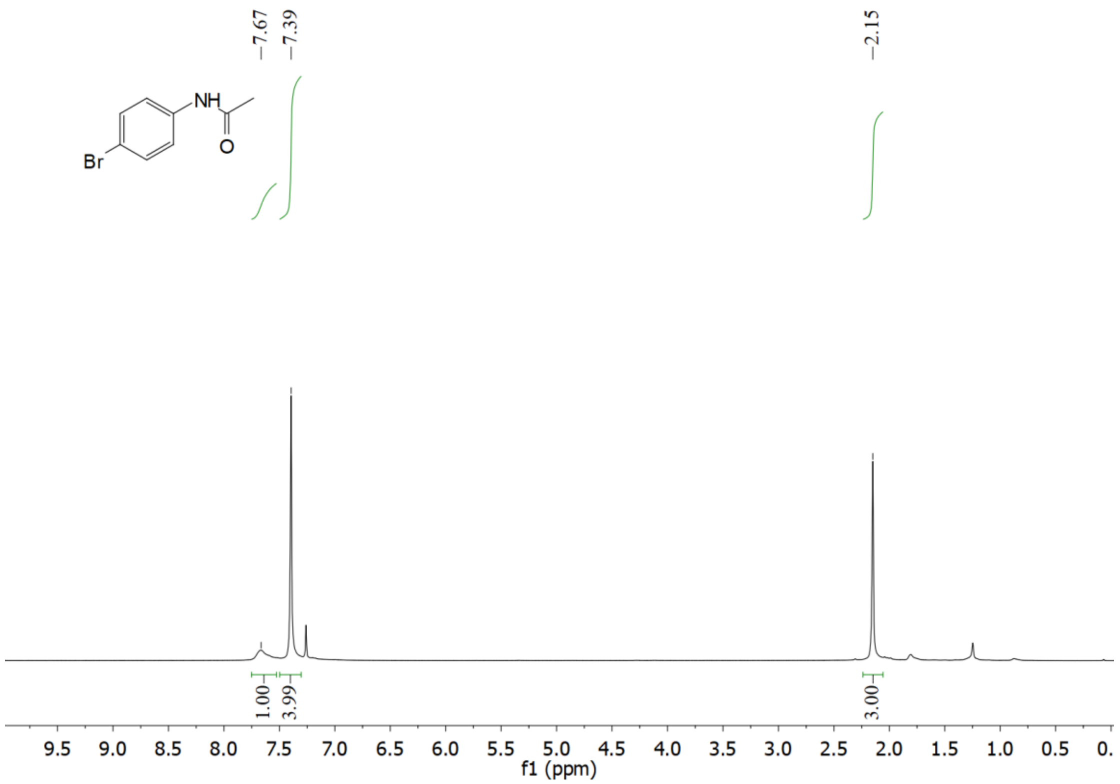

^{1}H NMR (400 MHz, $CDCl_3$) of **2ag**

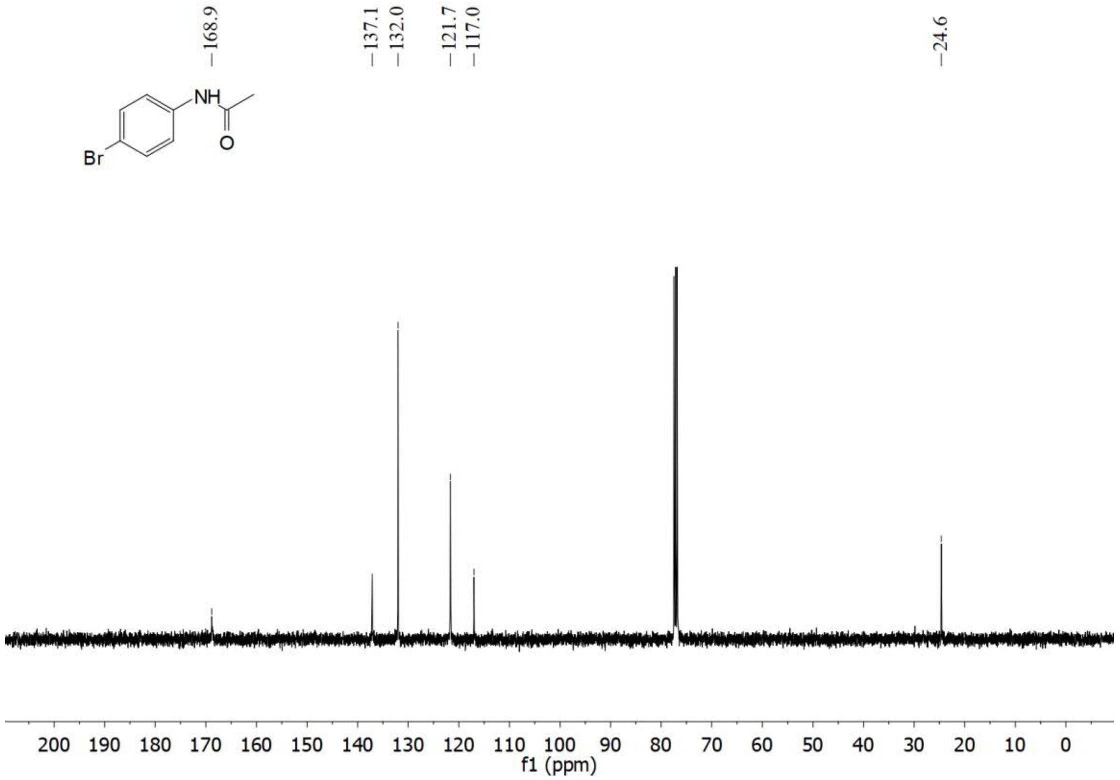

^{13}C NMR (101 MHz, $CDCl_3$) of **2ag**

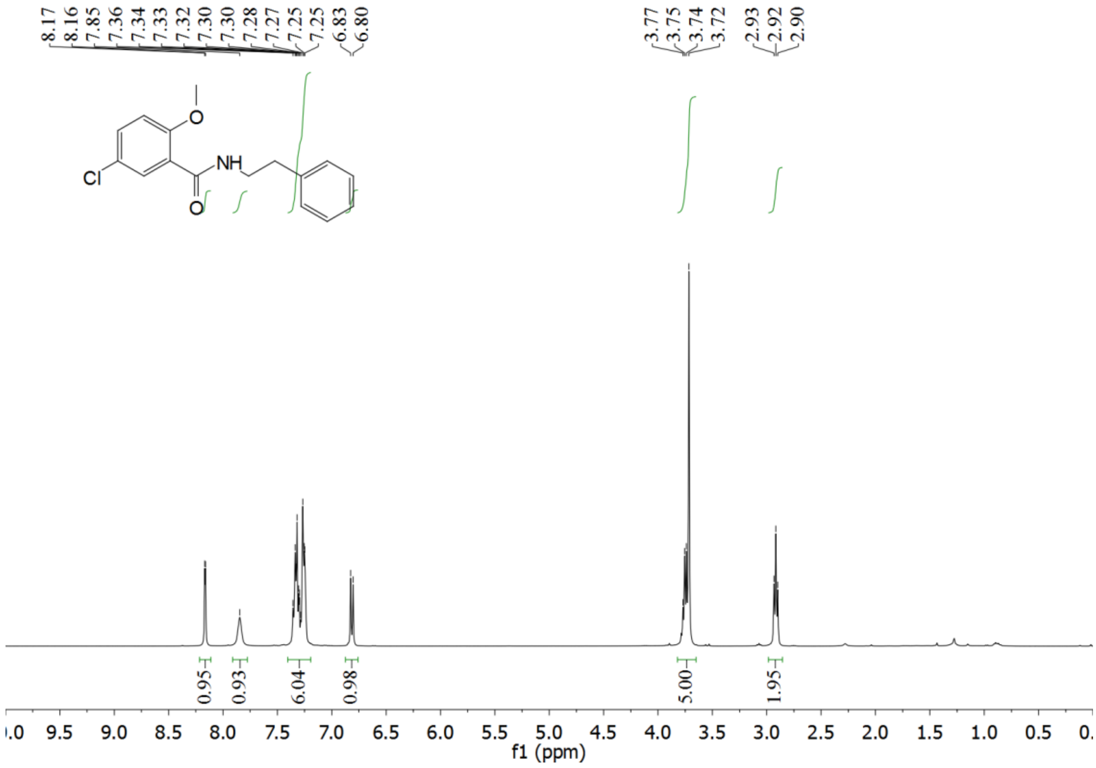

^{1}H NMR (400 MHz, $CDCl_3$) of **8**

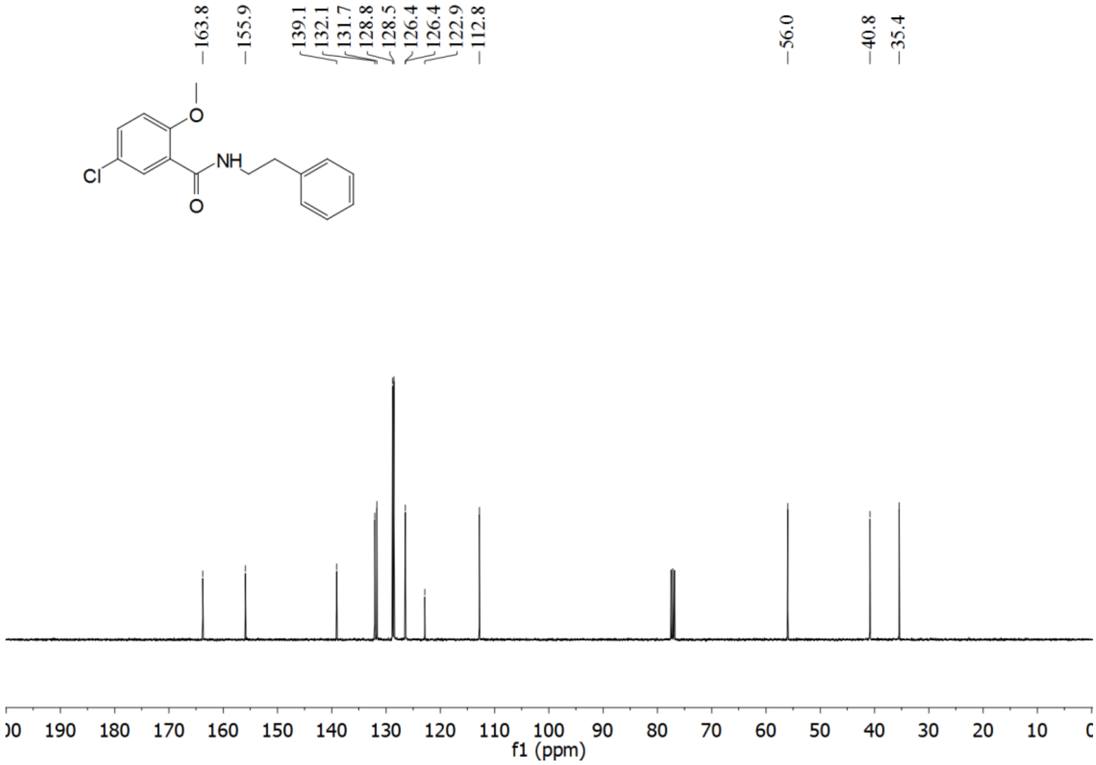

^{13}C NMR (101 MHz, $CDCl_3$) of **8**

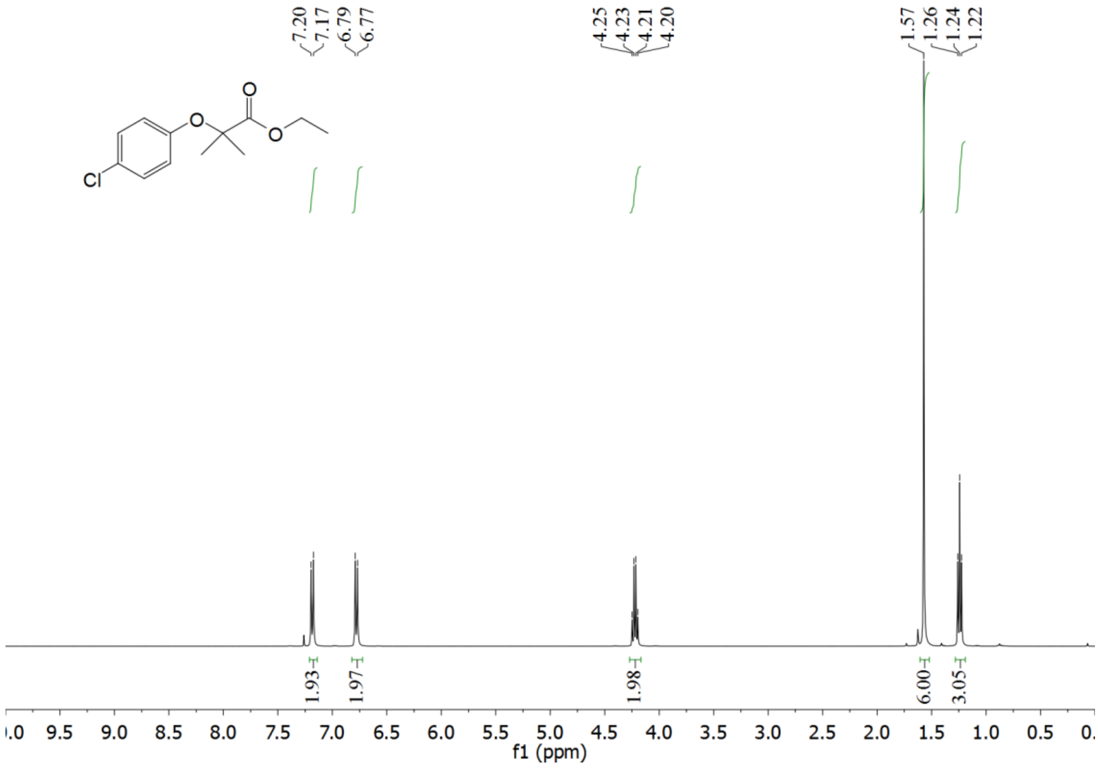

^{1}H NMR (400 MHz, $CDCl_3$) of **10a**

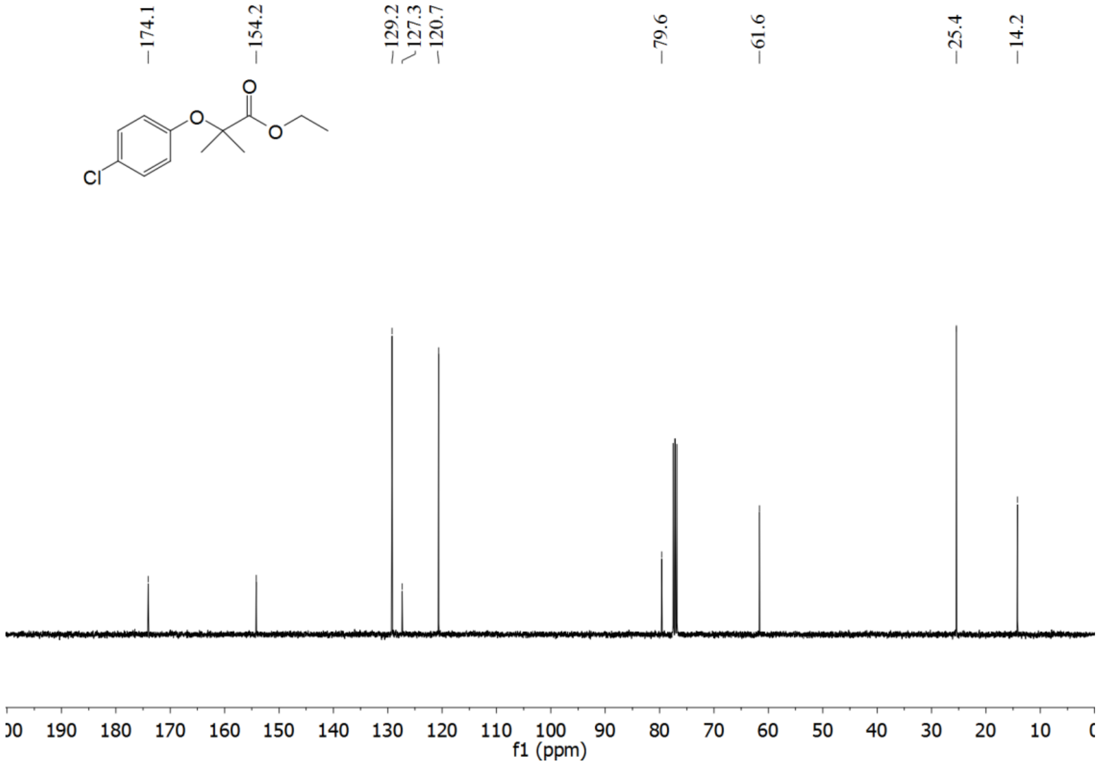

^{13}C NMR (101 MHz, $CDCl_3$) of **10a**

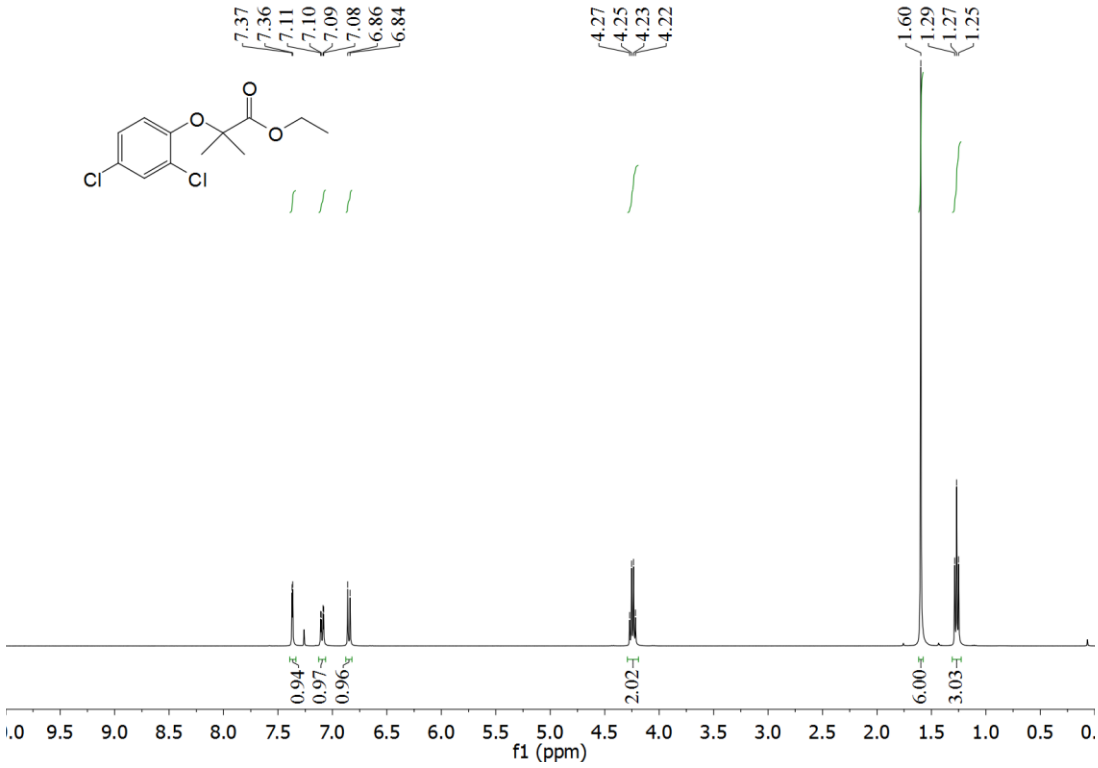

^{1}H NMR (400 MHz, $CDCl_3$) of **10a'**

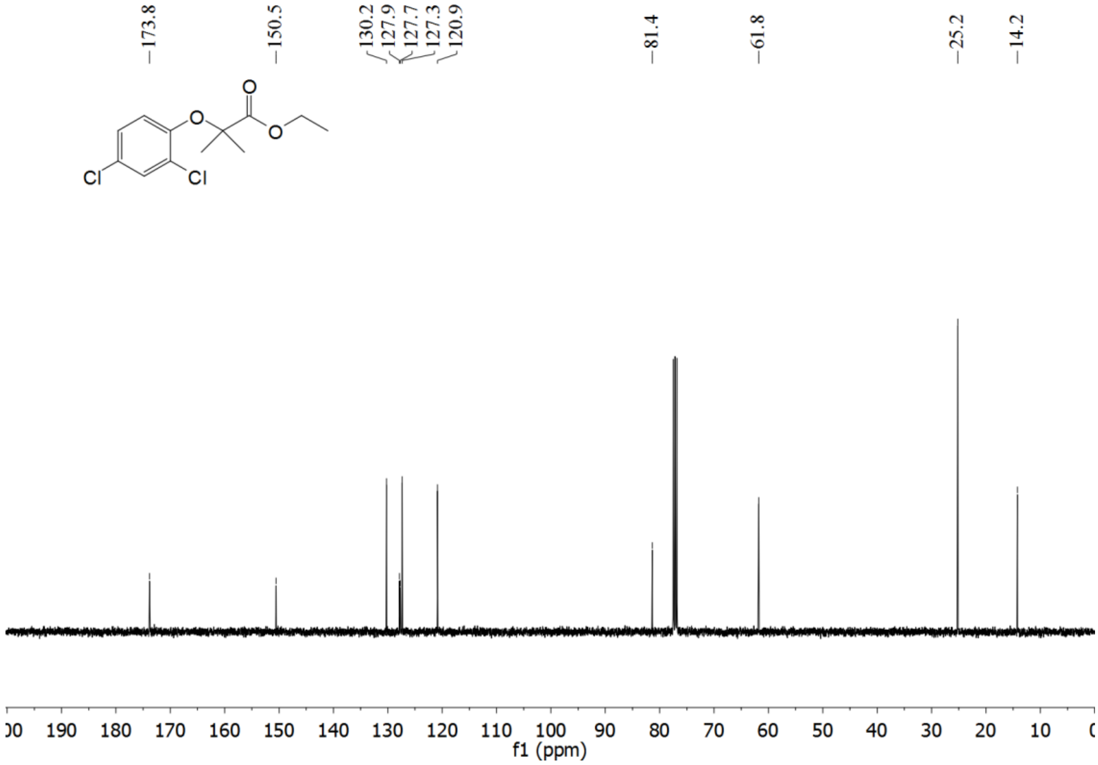

^{13}C NMR (101 MHz, $CDCl_3$) of **10a'**

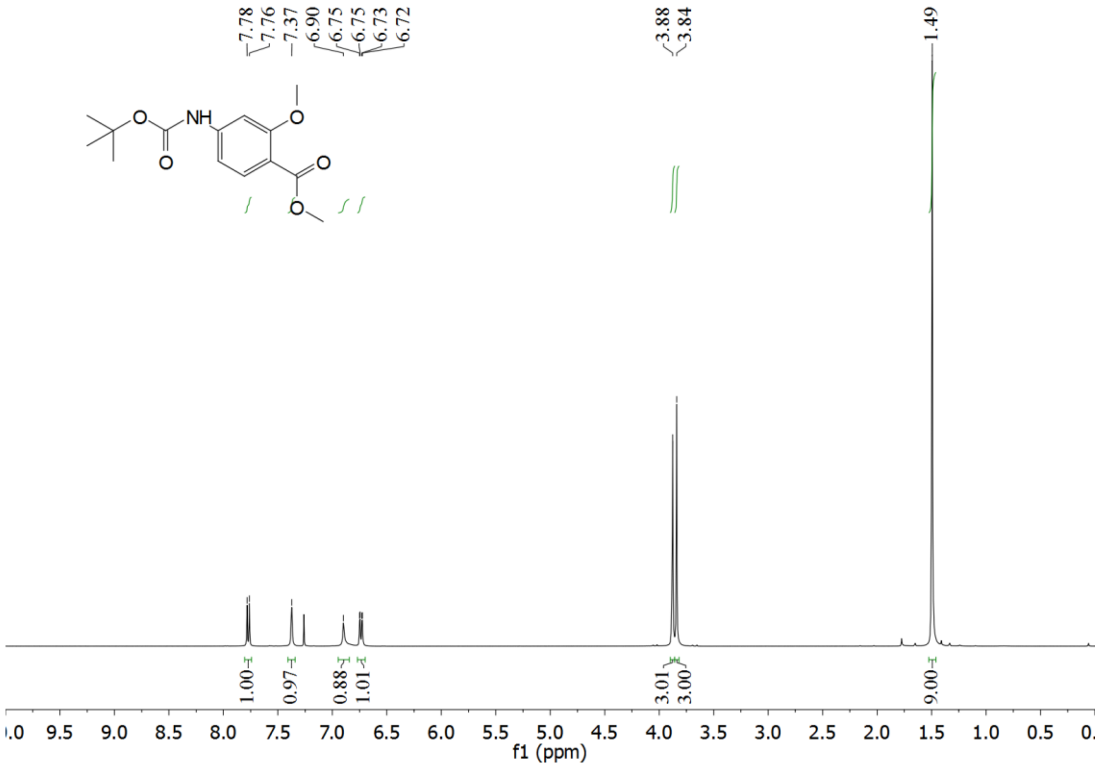

^{1}H NMR (400 MHz, $CDCl_3$) of **11**

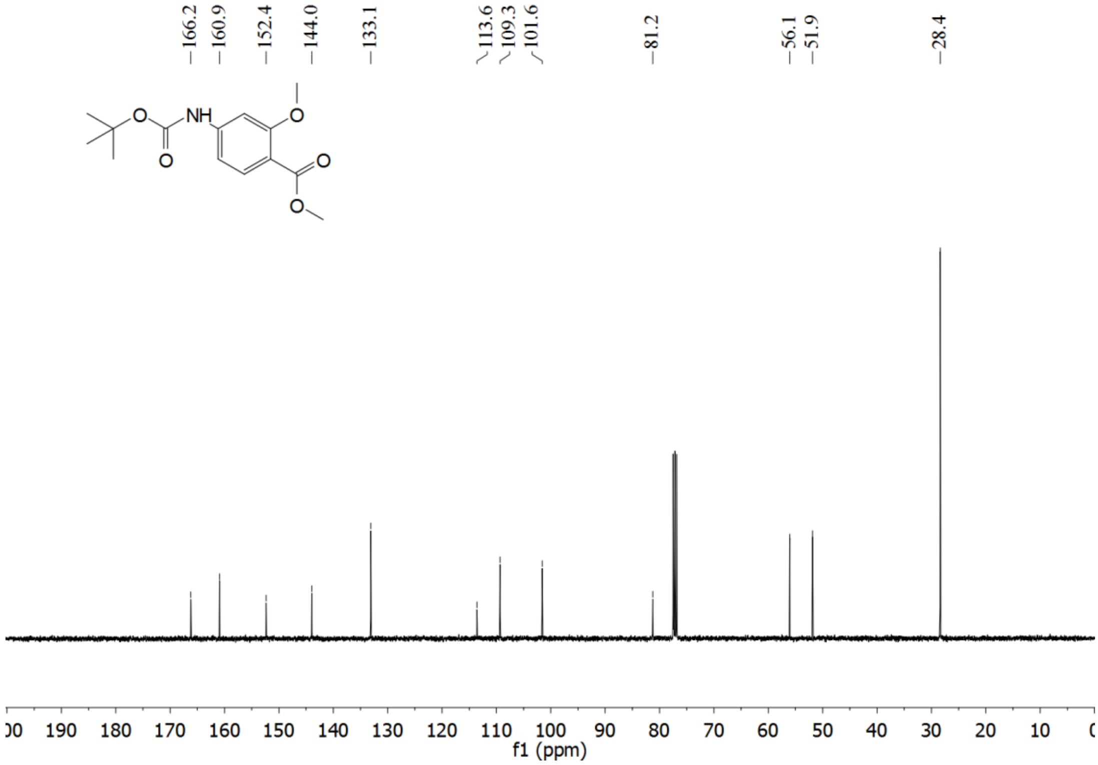

^{13}C NMR (101 MHz, $CDCl_3$) of **11**

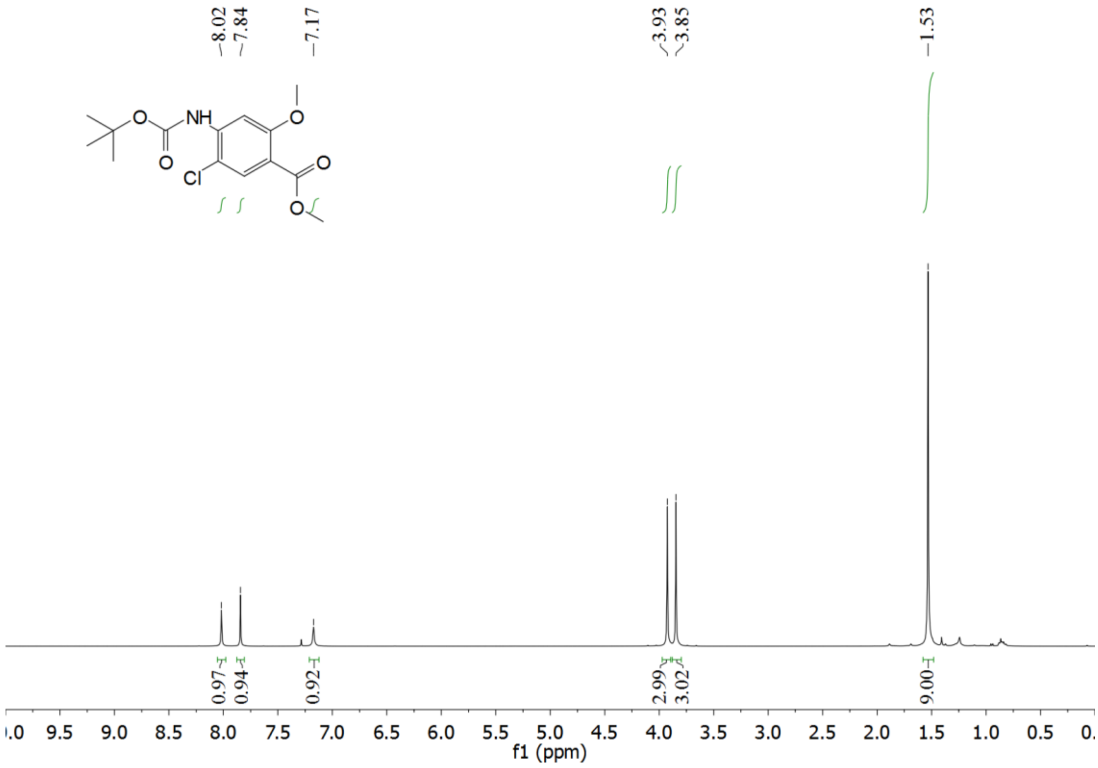

^{1}H NMR (400 MHz, $CDCl_3$) of **12a**

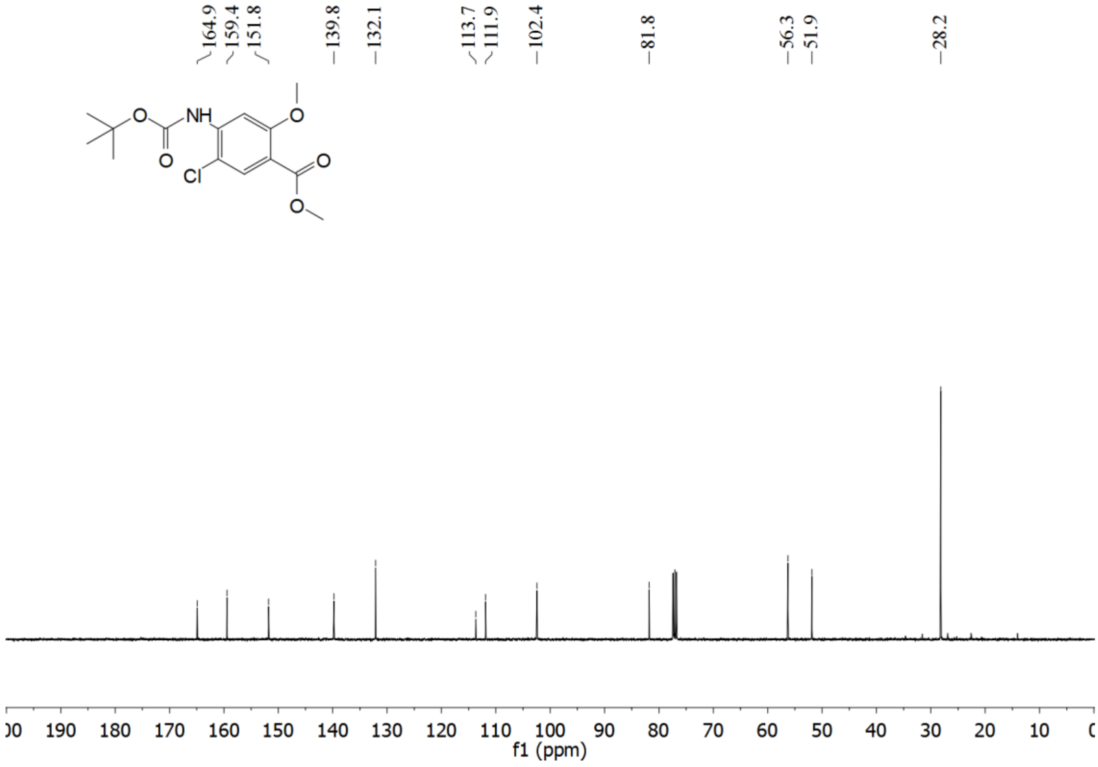

^{13}C NMR (101 MHz, $CDCl_3$) of **12a**

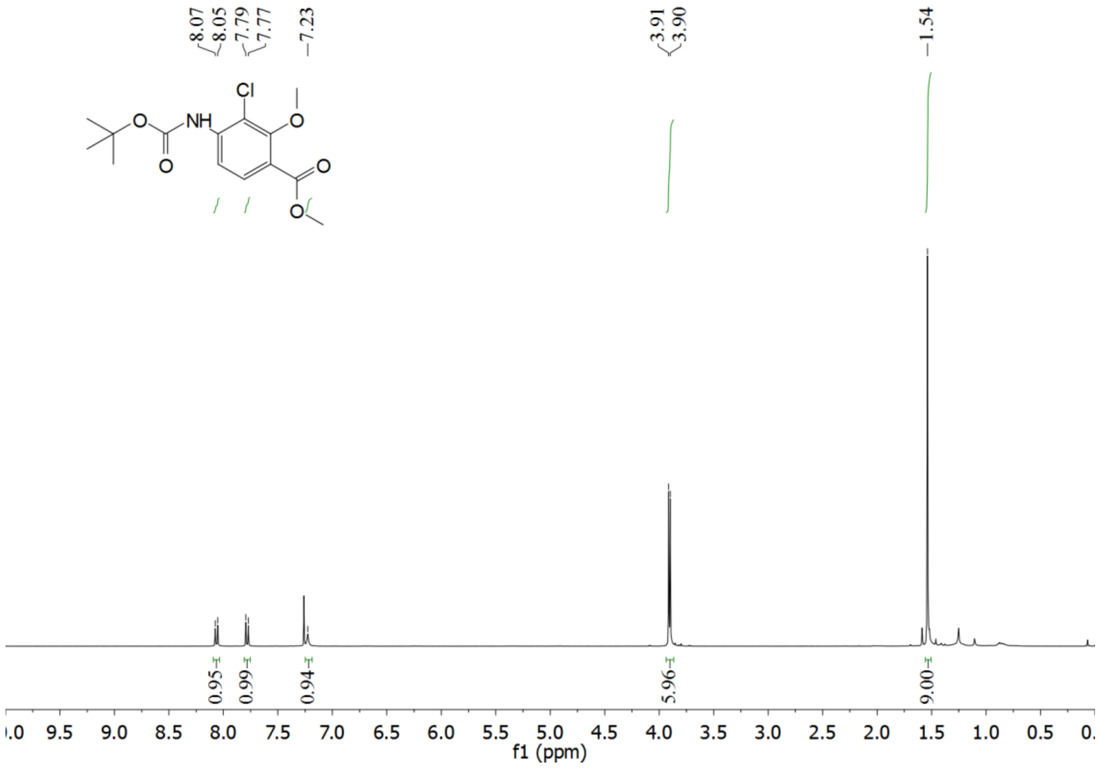

^{1}H NMR (400 MHz, $CDCl_3$) of **12a’**

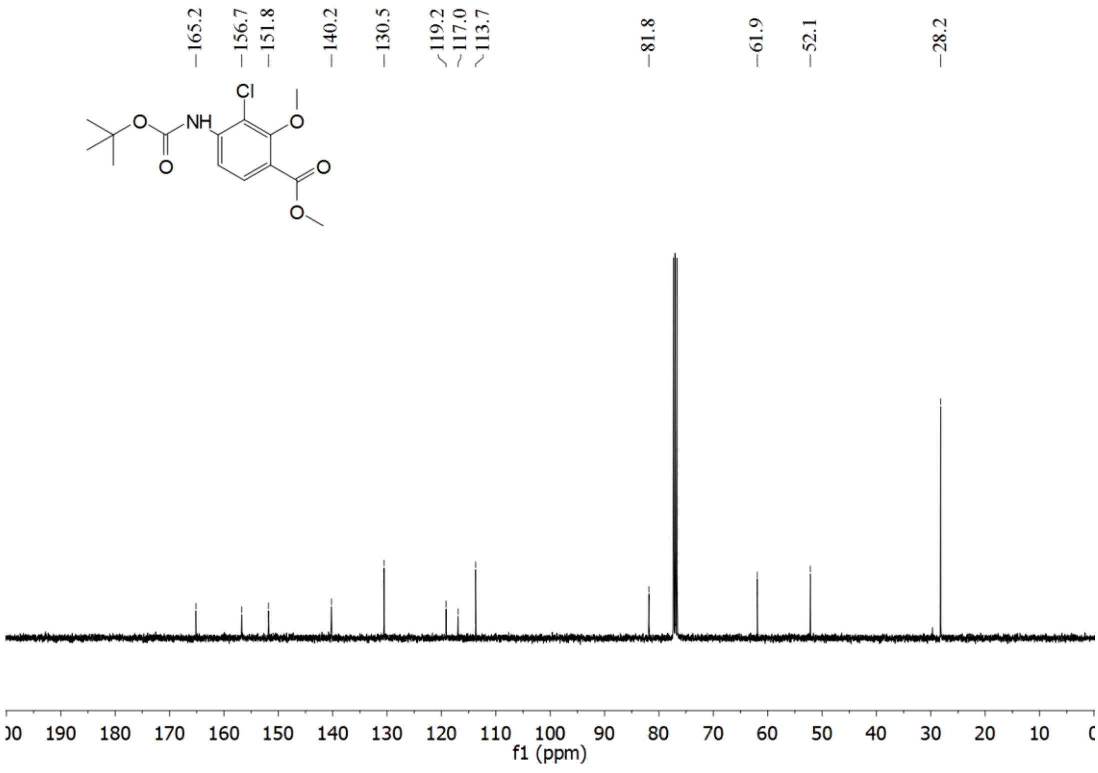

^{13}C NMR (101 MHz, $CDCl_3$) of **12a’**
